# Accurate Transcription Factor Activity Inference to Decipher Cell Identity from Single‐Cell Transcriptomic Data with MetaTF

**DOI:** 10.1002/advs.202410745

**Published:** 2025-05-21

**Authors:** Yongfei Hu, Yuanyuan Zhu, Guangjue Tang, Ming Shan, Puwen Tan, Ying Yi, Xiyuan Zhang, Man Liu, Xinyu Li, Le Wu, Jia Chen, Hailong Zheng, Yan Huang, Zhuan Li, Xiaobo Li, Dong Wang

**Affiliations:** ^1^ Department of Bioinformatics, Guangdong Province Key Laboratory of Molecular Tumor Pathology School of Basic Medical Sciences Southern Medical University Guangzhou 510515 China; ^2^ Dermatology Hospital Southern Medical University Guangzhou 510091 China; ^3^ Department of Pathology, School of Basic Medical Sciences Harbin Medical University Harbin 150081 China; ^4^ Department of Breast Surgery Harbin Medical University Cancer Hospital Harbin 150000 China; ^5^ Cancer Research Institute School of Basic Medical Sciences Southern Medical University Guangzhou 510515 China; ^6^ Key Laboratory of Functional Proteomics of Guangdong Province Department of Developmental Biology School of Basic Medical Sciences Southern Medical University Guangzhou 510060 China; ^7^ Department of Bioinformatics Fujian Key Laboratory of Medical Bioinformatics School of Medical Technology and Engineering Fujian Medical University Fuzhou 350122 China

**Keywords:** cell identity, scRNA‐seq, transcription factor activity, tumor immune microenvironment

## Abstract

Cellular heterogeneity within cancer tissues determines cancer progression and treatment response. Single‐cell RNA sequencing (scRNA‐seq) has provided a powerful approach for investigating the cellular heterogeneity of both cancer cells and stroma cells in the tumor microenvironment. However, the common practice to characterize cell identity based on the similarity of their gene expression profiles may not really indicate distinct cellular populations with unique roles. Generally, the cell identity and function are orchestrated by the expression of given specific genes tightly regulated by transcription factors (TFs). Therefore, deciphering TF activity is essential for gaining a better understanding of the uniqueness and functionality of each cell type. Herein, metaTF, a computational framework designed to infer TF activity in scRNA‐seq data, is introduced and existing methods are outperformed for estimating TF activity. It presents the improved effectiveness in characterizing cell identity during mouse hematopoietic stem cell development. Furthermore, metaTF provides a superior characterization of the functional identity of breast cancer epithelial cells, and identifies a novel subset of neural‐regulated T cells within the tumor immune microenvironment, which potentially activates *BCL6* in response to neural‐related signals. Overall, metaTF enables robust TF activity analysis from scRNA‐seq data, significantly enhancing the characterization of cell identity and function.

## Graphical abstract

MetaTF is a new computational framework that uses single‐cell RNA sequencing (scRNA‐seq) data to infer transcription factor activity with the aim to help in deciphering cells identity starting from a heterogeneous population, and apply it to characterize mouse hematopoietic stem cells and breast cancer epithelial cells, thus identifying a novel subset of neural‐regulated T cells within the tumor microenvironment.

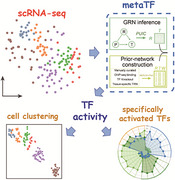

## 1 Introduction

Single‐cell RNA sequencing (scRNA‐seq) has become the state‐of‐the‐art approach for investigating the cellular heterogeneity and dynamics of various biological systems, ranging from cell development to disease progression.^[^ ^1^, ^2^, ^3^ ^]^ Leveraging the power of scRNA‐seq, researchers have successfully applied it for the identification of novel cell types and subtypes, the refinement of developmental lineage trajectories, and more.^[^ ^4^, ^5^, ^6^ ^]^ Regardless of what objective is pursued, a fundamental step consistently involves the establishment of cell identity present within a given tissue sample.^[^ ^7^, ^8^ ^]^ A common practice for identifying cell types is by clustering cells based on the similarity of their gene expression profiles.^[^ ^9^, ^10^ ^]^ However, transcriptional differences that separate cells into distinct groups may not necessarily indicate functionally distinct cell populations.^[^ ^11^, ^12^, ^13^ ^]^ Given the additional layers of complexity in cell identity, accurately defining cell identity remains a substantial challenge.

At the core of cell identity and function lies the intricate interplay of gene expression programs, which ultimately dictate the unique characteristics of each cell type. Central to this regulatory landscape are the core regulatory circuits, orchestrated by tightly regulated transcription factors (TFs).^[^ ^7^, ^14^ ^]^ Understanding TF activity is paramount for unraveling the essence and functionality of distinct cell types. While recent years have seen the emergence of several algorithms for analyzing TF activity in scRNA‐seq data, they often grapple with limitations affecting their accuracy or robustness.^[^ ^15^, ^16^, ^17^, ^18^ ^]^ For instance, the algorithms solely relying on TF expression levels struggle to faithfully capture their biological activity. Similarly, those scrutinizing both TFs and their direct target expression encounter challenges in identifying genuine targets due to the complexities of regulation. These methods rely heavily on experimentally measured or predicted TF binding information, such as motif enrichment, thereby limiting their scope to TFs with available data. Moreover, current TF binding site (TFBS) annotation assays, such as chromatin immunoprecipitation coupled with deep sequencing (ChIP‐seq), ChIP‐exo, and CUT&RUN, necessitate high‐affinity antibodies and large amounts of homogeneous cells, posing challenges for diverse tissues. Additionally, these assays assess only one TF at a time, hampering comprehensive TF binding analysis. Conversely, motif‐based predictions, while not require cell‐specific data, rely on predefined motifs available for only a subset of TFs, leading to potential false positives.

Recognizing these limitations, the compilation of TF–target interactions from diverse data sources to construct a comprehensive static TF–target interaction network, integrated it with dynamic gene regulatory networks (GRNs) from single cells, provide a better understanding of regulatory relationships within the sample's organization under specific conditions. Furthermore, a notable observation emerges; there exists an underutilization of TF activity in characterizing cell identity and function, especially in contexts like cell development and disease progression.^[^ ^19^, ^20^ ^]^ This underutilized potential underscores the urgent need for enhanced algorithms for inferring TF activity in scRNA‐seq data and their practical application.

In this context, we introduce metaTF (https://github.com/wanglabsmu/metaTF), a computational framework tailored for comprehensive TF activity analysis in scRNA‐seq data. MetaTF addresses the limitations of traditional methods by constructing comprehensive static prior networks through the integration of diverse data sources, and it does not require training on a specific scRNA‐seq dataset. It enhances TF regulation portrayal by amalgamating dynamic regulatory networks from scRNA‐seq data with static prior networks and optimizing existing algorithms to provide accurate and robust TF activity predictions and gene regulatory network analysis. Benchmarking across diverse types of scRNA‐seq data, including TF knockout, and TF overexpression data, demonstrates that metaTF consistently outperforms other widely used tools, affirming its remarkable accuracy and resilience in inferring TF activity from scRNA‐seq data.

Single‐cell Clustered Regularly Interspaced Short Palindromic Repeats (CRISPR) screening is revolutionizing the field of immuno‐oncology by enabling the precise mapping of GRNs and the identification of critical targets. Specifically, the knockout of TFs enables the determination of the perturbation effects in finely regulated biological processes, which in turn influences cellular functions. In the realm of CD8^+^ cytotoxic T‐cell differentiation,^[^ ^21^ ^]^ metaTF has not only proven its exceptional capability in the context of gene regulatory network analysis but has also displayed its prowess by showcasing its ability to predict the effects of TFs’ perturbations with remarkable accuracy. These underscore the enhanced capability of metaTF to effectively interpret novel forward genetic screens data, highlighting its advanced analytical prowess in decoding the complex genetic interactions. MetaTF provides an effective and applicable solution to elucidate perturbation function and biologic circuits by a model‐based quantitative analysis of single‐cell‐based CRISPR screening data. As an extension of its application, metaTF can quantify the relationships between various perturbations.

Its application to mouse hematopoietic stem cell (HSC) data^[^ ^22^ ^]^ illustrates how TF activity enhances the accurate identification of cell identity. Additionally, metaTF provides a deeper understanding of the functional identity of epithelial cells in breast cancer (BRCA), unveiling the presence of a novel neural‐regulated T‐cell subset within the tumor immune microenvironment. In summary, metaTF addresses the need for methods capable of integrated analysis of diverse data types by offering a systematic integration method that is broadly applicable. By leveraging various data sources and dimensions of TF biology, metaTF provides a more comprehensive and versatile solution for TF activity inference and gene regulatory network analysis. This integrated analysis is crucial for unraveling the complexities of gene regulation and holds immense potential for researchers across various domains.

## 2 Results

### 2.1 Overview of the MetaTF Framework

**Figure 1:**
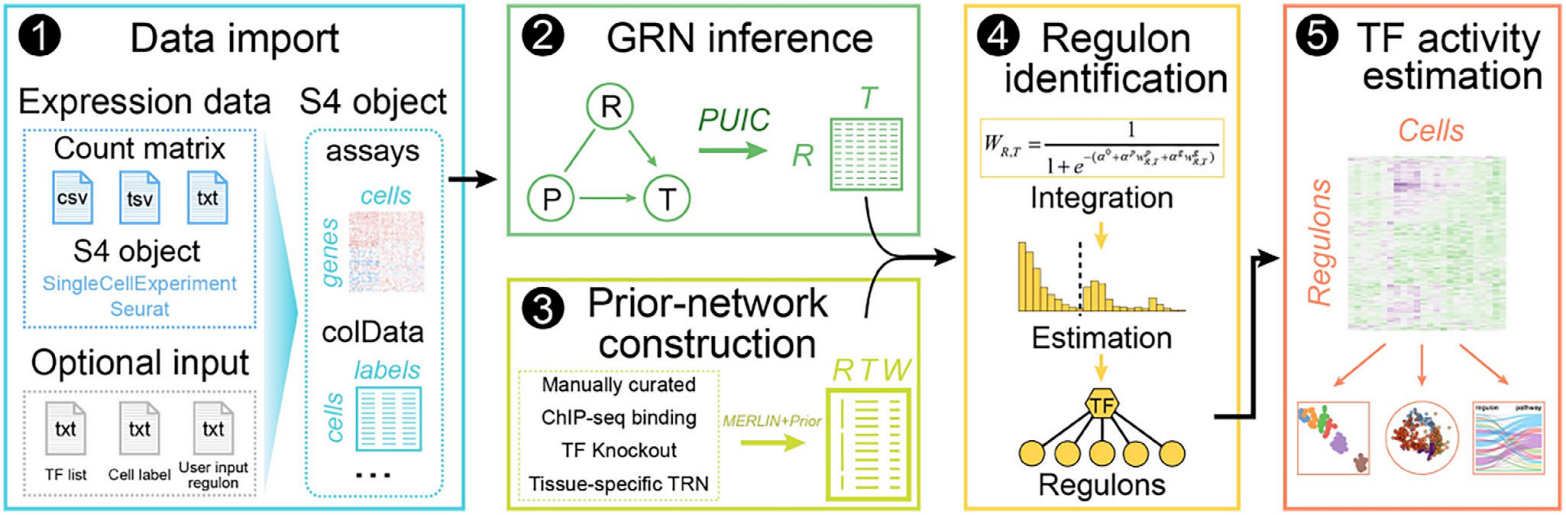
Overview of metaTF framework. MetaTF is built upon the S4 class SingleCellExperiment package and accepts various types of inputs, including count matrices and S4 objects generated from SingleCellExperiment or Seurat. Subsequent analyses include GRN inference, prior‐network construction, regulon identification, and TF activity estimation. Moreover, metaTF provides several additional functions, including cell type‐specific activated TF identification, regulon pathway enrichment analysis, and the construction of various visual aids, such as dot plots, Radviz plots, Sankey diagrams, and heatmaps.

MetaTF is an integrated framework for inferring TF activity from scRNA‐seq data. This workflow consists of five key components: data import, GRN inference, construction of a prior network, regulon (TF–targets) identification, and estimation of TF activity (**Figure** **1**). Specifically, metaTF supports flexible data input, including count matrices from droplet or full‐length protocols,^[^ ^23^ ^]^ or preprocessed data objects from software such as CellRanger,^[^ ^24^ ^]^ Seurat,^[^ ^25^ ^]^ and SingleCellExperiment.^[^ ^26^ ^]^ GRN inference utilizes percentage of unique information contribution (PUIC), an entropy‐based algorithm, to estimate the importance of regulator–target relationships by quantifying the unique informational contribution from the mutual information (MI) between each gene triplet (Figure 1; Experimental Section; Figure S1a, Supporting Information). Notably, integration with a weighted prior TF–target network constructed from literature‐curated, ChIP‐seq, TF knockout, and tissue‐specific interaction information further enhances robust regulon identification for each TF (Experimental Section; Figure S1b, Supporting Information). The Virtual Inference of Protein‐activity by Enriched Regulon analysis (VIPER)^[^ ^17^ ^]^ algorithm then estimates TF activity based on the enrichment score of the identified regulons. Implementation of metaTF as an R package enables leveraging the extensive SingleCellExperiment ecosystem for convenience and scalability. Meanwhile, parallel workflows allow comparisons of alternative methods for GRN inference (such as Gene Network Inference by Ensemble of Trees (GENIE3)^[^ ^27^ ^]^ and PCOR (R Package for a Fast Calculation to Semi‐partial Correlation Coefficients)^[^ ^28^ ^]^ and estimation of TF activity [such as single‐sample Gene Set Enrichment Analysis (ssGSEA)^[^ ^29^ ^]^ and AUCell^[^ ^18^ ^]^ (a tool to quantify the enrichment of gene sets in single cells)]. Downstream analyses include cell‐type‐specific activated TF identification, pathway enrichment, and the construction of visual aids, including Radviz plots,^[^ ^30^ ^]^ Sankey diagrams, and heatmaps (see online vignette). Optimization via vectorization and multithreading ensures user‐friendly and timely analysis.

### 2.2 Benchmarking Highlights MetaTF's Superior Performance for Inferring TF Activity

**Figure 2:**
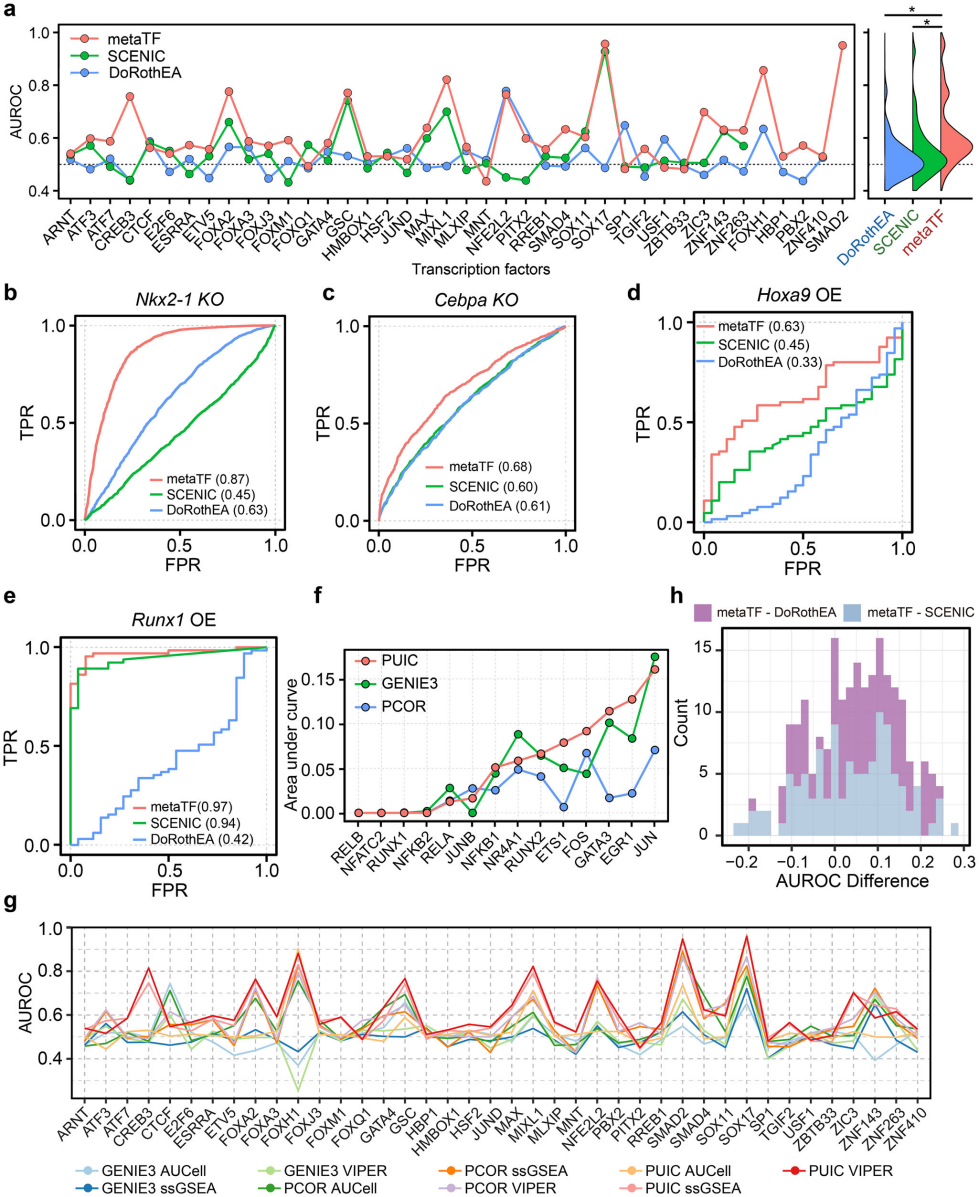
Assessment of metaTF using various types of scRNA‐seq data. a) Line plot on the left depicting the AUROC for 40 perturbated TFs, calculated with three methods including metaTF, SCENIC, and DoRothEA. The distribution curve on the right illustrates the AUROC score distributions for these three methods. The Kolmogorov–Smirnov (KS) test was used to determine the differences (*P < 0.05). b) Receiver operating characteristic (ROC) curves for *Nkx2‐1* activity estimation by metaTF, SCENIC, and DoRothEA. These estimations were conducted on *Nkx2‐1* conditional knockout (cKO) epithelial cells from Little et al.,^[^ ^33^ ^]^ with the AUROC values indicated in brackets. FPR represents the false‐positive rate, and TPR represents true‐positive rate. c) ROC curves for *Cebpa* activity estimation by the same three methods applied to *Cebpa* cKO myeloid progenitors from Paul et al.^[^ ^34^ ^]^ d) ROC curves of *Hoxa9* activity estimation by the same three methods applied to *Hoxa9* overexpression HECs from Guo et al.^[^ ^35^ ^]^ e) ROC curves of *Runx1* activity estimation by the same three methods applied to *Runx1* overexpression HECs from Guo et al. f) Line plot showing the AUC values for 14 perturbed TFs, calculated using GRNs inferred by PUIC, GENIE3, and PCOR in the CROP‐seq dataset. g) Line plot showing the AUROC values for 40 perturbed TFs, calculated using various combinations of GRN inference methods and TF activity enrichment methods within the metaTF framework. h) The histogram displays the distribution of differences in the AUROC values obtained from metaTF, DoRothEA, and SCENIC for each transcription factor, providing a visual summary of their comparative performance on data of single‐cell CRISPR screens from CD8^+^ cytotoxic T‐cell differentiation.

We systematically benchmarked metaTF against the state‐of‐the‐art algorithms Single‐cell Regulatory Network Inference and Clustering (SCENIC)^[^ ^18^ ^]^ and DoRothEA.^[^ ^15^ ^]^ SCENIC uses machine learning (GENIE3/GRNBoost) to infer GRNs and refines them via motif enrichment analysis, retaining only TF–target pairs with significant motif presence in the promoters of target genes. This approach reduces indirect interactions but is limited by the availability of predefined motifs and may yield false positives. DoRothEA relies on a precompiled TF–target interaction database, bypassing GRN inference from single‐cell data. Although efficient, it may include context–irrelevant interactions, leading to false positives. By using diverse scRNA‐seq datasets, metaTF showed its superior performance in several scenarios. In CRISPRi perturbation data targeting 40 TFs in human embryonic stem cells (ESCs),^[^ ^31^ ^]^ metaTF outperformed SCENIC and DoRothEA at inferring TF activities (**Figure** **2**a; Experimental Section; Figure S2a and Tables S1 and S2, Supporting Information). Since dropouts pose challenges for TF activity analysis, we evaluated their performance under varying dropout levels. Despite observing declining performance with increased dropout rates, it is noteworthy that metaTF maintains competitive performance compared to other tools (Experimental Section; Figure S2b, Supporting Information), demonstrating the robustness of metaTF with dropouts. This is further supported by results from low‐coverage 10× Genomics scRNA‐seq data^[^ ^32^ ^]^ (Experimental Section; Figure S3, Supporting Information). In primary cells with single TF knockout, metaTF achieved Area Under the Receiver Operating Characteristic Curve (AUROC) values of 0.87 and 0.68 for *Nkx2‐1* knockout droplet‐based scRNA‐seq data^[^ ^33^ ^]^ and *Cebpa* knockout MARS‐seq data,^[^ ^34^ ^]^ respectively, which were higher than those produced by the alternatives (Figure 2b,c; Experimental Section; Figure S4a–d and Table S3, Supporting Information). In overexpression data, metaTF again excelled with AUROC values of 0.63 and 0.97 separately for the *Hoxa9* and *Runx1* overexpression Smart‐seq2 data^[^ ^35^ ^]^ (Figure 2d,e; Experimental Section; Figure S4e,g and Tables S4 and S5, Supporting Information). These results affirm the remarkable accuracy and resilience of metaTF in conducting TF activity inference for a wide range of scRNA‐seq datasets. Specifically, when applied to CRISPR droplet sequencing (CROP‐seq) data from HEK293T cells with TF perturbations,^[^ ^36^ ^]^ PUIC outshines the other two methods (GENIE3 and PCOR) in more than half of the TFs, and maintains a faster computational speed (Figure 2f; Experimental Section; Figure S4h, Supporting Information). Furthermore, the integration of GRN inferred by PUIC and the prior TF–target network significantly contributed to the enhanced performance of metaTF (Experimental Section; Figure S4i,j, Supporting Information). Lastly, in terms of TF activity estimation, VIPER was proven to be superior to ssGSEA and AUCell (Experimental Section; Figure S4k and Table S6, Supporting Information). Besides, combinatorial analysis on CRISPRi data showed that the default PUIC and VIPER methods in metaTF yielded optimal performance (Figure 2g), achieving the highest AUROC values for 23 out of 40 TFs (Table S7, Supporting Information). Meanwhile, to elucidate the pivotal contributions of two key steps in inferring TF activity using metaTF, we recalculated the activities of 35 TFs by separately removing the steps involving PUIC for GRN calculation and the prior network. The findings revealed that, in comparison to PUIC alone, the inclusion of the prior network led to superior results for metaTF. Simultaneously, when excluding the PUIC calculation of the GRN step and retaining only the prior network, metaTF achieved results comparable to SCENIC (Figure S5a,b, Supporting Information). In summary, systematic benchmarking validates metaTF as a superior algorithm for scRNA‐seq TF activity inference.

Single‐cell CRISPR screening facilitates the mapping of gene regulatory networks, uncovering pivotal targets in immuno‐oncology. Specifically, the knockout of TFs enables the determination of the perturbation effects in finely regulated biological processes, which in turn influences cellular functions. In the realm of CD8^+^ cytotoxic T‐cell differentiation,^[^ ^21^ ^]^ single‐cell CRISPR screens with knockout of TFs provide a more effective method for interrogating the regulatory landscape of these cells. Our metaTF method demonstrated superior performance in analyzing these datasets with single TF knockout experiments, achieving an average AUROC of 0.69 across 109 TFs. Notably, metaTF outperformed established methods DoRothEA and SCENIC in 80 and 66 TFs, respectively (Figure 2h; Experimental Section; Figure S6a, Supporting Information). As an extension of its application, we initially identified eight distinct pairs of TFs that exhibited divergent activity trajectories following double knockout experiments, highlighting the intricate regulatory interdependencies within CD8^+^ T‐cell regulation (Figure S6b,c, Supporting Information). This comprehensive analysis unveiled the complex nature of TF interactions and their roles in the cellular response. These results not only validate metaTF's robustness in interpreting single‐cell CRISPR screen data but also provide insights into the intricate TF networks governing T‐cell fate decisions. We also validated similar conclusions using single‐cell CRISPR screening data from the cortical development of the mouse brain (Experimental Section; Figure S7, Supporting Information). By elucidating both individual TF activities and their functional interplay, our approach offers a framework for deciphering GRN complexity in various biological contexts, potentially informing targeted approaches in immuno‐oncology.

### 2.3 TF Activity Profiles Outperform Expression Profiles for Dissecting Heterogeneous Cell Populations

**Figure 3:**
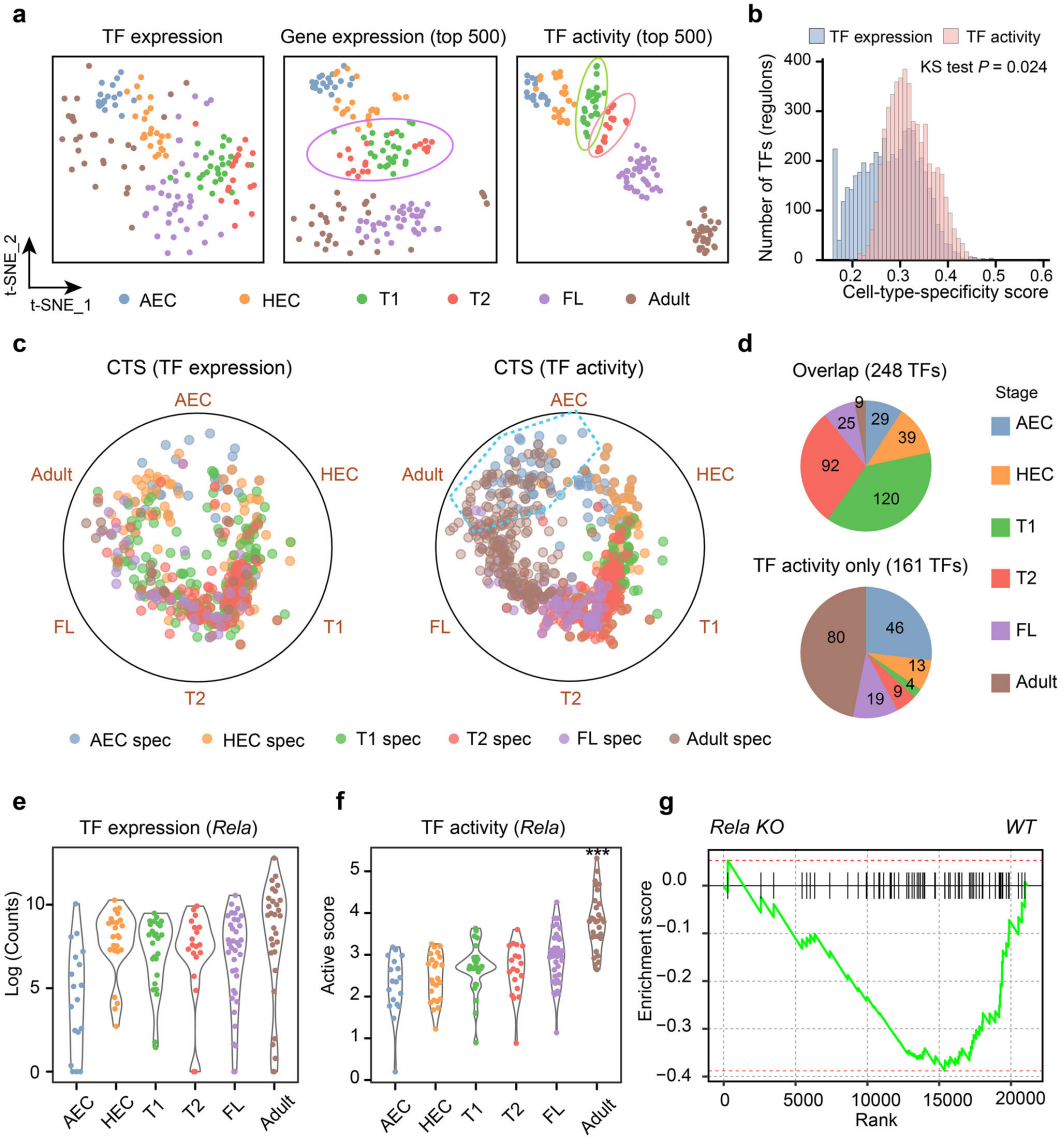
Comparison between the TF expression and TF activity profiles. a) t‐SNE plots visualizing cluster assignments of cells based on TF expression profile (left), highly variable gene expression profile (middle) and TF activity profile (right). The colors indicate the categorization into six sequential cell populations during HSC development: arterial endothelial cells (AEC), hemogenic endothelial cells (HEC), T1 (type 1 pre‐HSCs), T2 (type 2 pre‐HSCs), fetal liver HSCs (FL), and Adult (adult HSCs). b) Bar plot representing the distribution of TFs at both the expression and activity levels, categorized by cell‐type‐specific score. The Kolmogorov–Smirnov (KS) test was used to determine the differences. c) Radviz plot showing the distribution of CTS TFs at the expression (left) and activity (right) levels, with colors representing the six cell types. d) The pie chart on the top displaying the number of shared TFs identified at both expression and activity levels, while the pie chart on the bottom displaying the number of TFs exclusively identified at the activity level. e) Violin plot demonstrating the expression of *Rela* in the six sequential populations. f) Violin plot demonstrating the activity score of *Rela* in the six sequential populations. Significance levels are indicated as ***, with a *P*‐value of <0.001. g) GSEA showing the relative enrichment of target genes regulated by *Rela* between WT (wild‐type) and Rela KO (knockout) samples. The normalized enrichment score was used to quantify the enrichment level.

To explore the potential of TF activity in capturing cellular heterogeneity, we utilized six sequentially acquired cell populations during the development of mouse embryonic HSCs in our previous study,^[^ ^22^ ^]^ including inferred TF activity profile, TF expression profile, and highly variable gene expression profiles (Experimental Section). Dimensionality reduction analysis visualizes exceptional capability in effectively discerning diverse populations based on TF activity profiles while maintaining high level of internal consistency within each population (**Figure** **3**a; Figures S8a and S9a, Supporting Information). Nonetheless, cell clustering based on TF expression or even highly variable gene expression faced challenges, particularly in the CD45^−^ T1 and CD45^+^ T2 pre‐HSC populations, which were also observed in the results obtained from employing a classic random forest classification model to fit TF activity profiles and gene expression profiles (Experimental Section; Figure S9b, Supporting Information). This indicates that aggregating signals from regulons in TF activity potentially delivers more resilient information content versus expression alone. To further confirm this notion, we gauged the cell‐type‐specific action and within cell‐type variation of TFs by contrasting activity and expression profiles (Experimental Section). In line with our hypothesis, we observed that TFs exhibited relatively higher cell type specificity (CTS, Figure 3b; Table S8, Supporting Information) and lower within cell‐type variance (Figure S8a, Supporting Information) in their activity versus expression profiles. As an example, the *Hes* family bHLH transcription factor 1 (*Hes1*), known for its role in the maintenance of the HSC pool in the bone marrow under stress,^[^ ^37^ ^]^ emerged as an adult HSC‐specific TF in the activity profile, while *Hes1* expression was confined to a small subset of adult HSCs, exhibiting high within‐cell‐type variation (Figure S8b,c, Supporting Information).

Robust cell state characterization using metaTF enables the discovery of additional CTS determinants. We further identified 414 CTS TFs, with the highest number in the T1 and T2 pre‐HSC stages (Figure 3c; Figure S8d,e, Supporting Information). While 60% (248) of CTS TFs were identified in both the activity and expression profiles (Figure 3d, upper panel), the activity profile uncovered 161 additional unique CTS TFs undetected by the expression profile (Figure 3c,d, lower panel), accounting for 40% of the total. Nearly half (80/161) of these TFs were activated specifically in adult HSCs, like *Rela* (Figure 3e,f) and *Sp1* despite negligible expression differences (Figure S8f,g, Supporting Information). We confirmed their activation in HSCs using independent *Rela* knockout microarray data^[^ ^38^ ^]^ (Figure 3g) and *Sp1* knockout microarray data^[^ ^39^ ^]^ (Experimental Section; Figure S8h, Supporting Information).

Additionally, by analyzing scRNA‐seq data from healthy human bone marrow plasma cells mapped to the T2T‐CHM13 reference assembly,^[^ ^40^ ^]^ we validated clustering patterns of Immunoglobulin A (IgA)/ Immunoglobulin G (IgG) plasma cells using TF activity analysis, revealing subtype‐specific TF activation and supporting previous observations of tissue‐origin transcriptional retention in bone marrow plasma cells (Experimental Section; Figure S10, Supporting Information). In summary, the TF activity profile outperforms the expression profile in enabling a refined dissection of heterogeneous cell populations and identification of novel cell‐type‐specific regulators.

### 2.4 MetaTF Unveils the Heterogeneity of Human Breast Cancer Epithelial Cells

Cellular heterogeneity within cancer tissues determines both cancer progression and treatment response. Breast cancer stands out as the first malignancy to be recognized for its molecular‐level heterogeneity, leading to the development of tailored treatment strategies for different molecular subtypes. This progress has been further propelled by the recent availability and accumulation of a wealth of scRNA‐seq data, which includes both normal and malignant breast epithelial cells, as well as immune cells within the breast cancer microenvironment.^[^ ^41^, ^42^, ^43^, ^44^ ^]^ Therefore, we utilized metaTF to explore cell identity in breast cancer development and the immune microenvironment.

**Figure 4:**
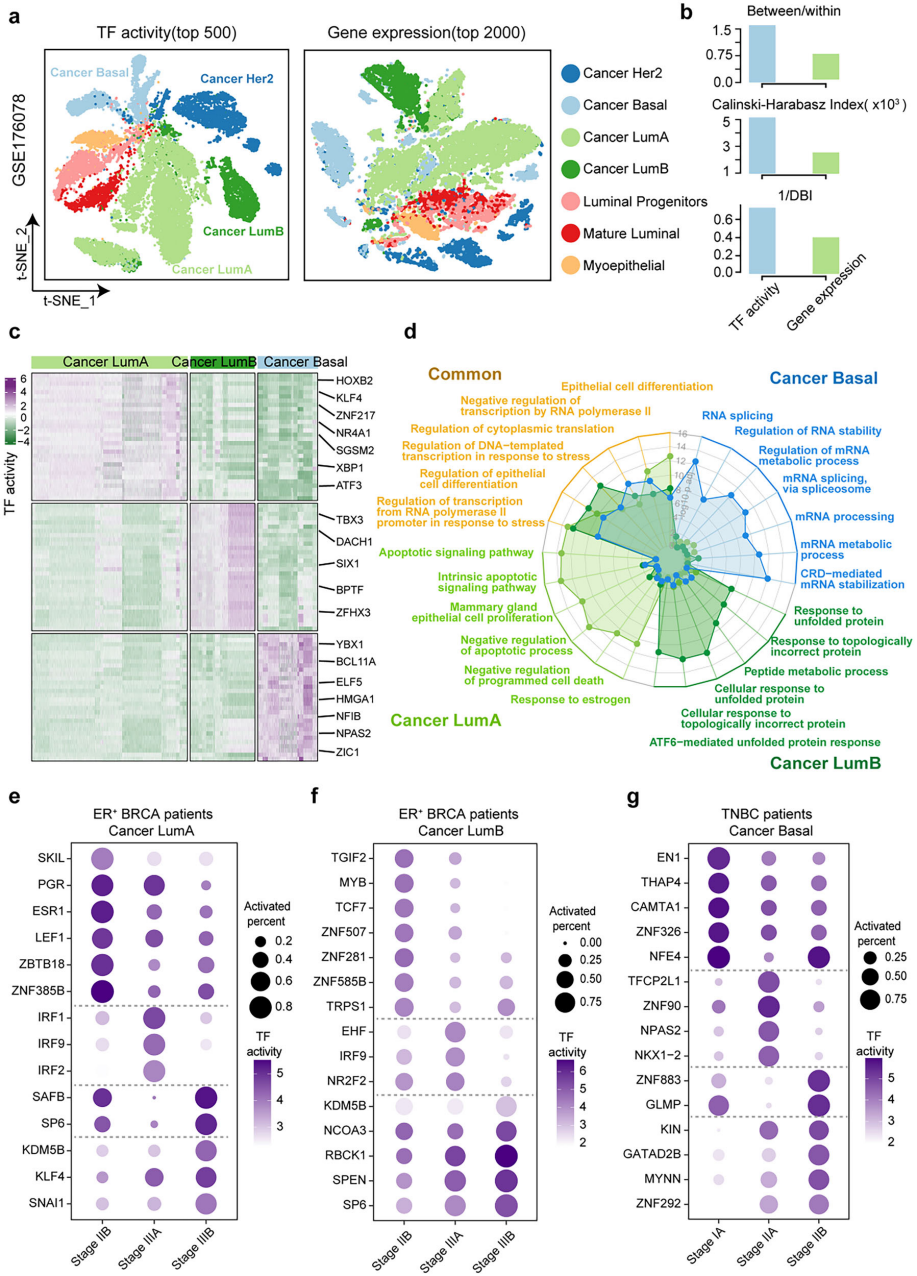
MetaTF results for the human breast cancer epithelial cells single‐cell RNA‐seq dataset. a) t‐SNE plots visualizing the cluster assignments of human breast cancer epithelial cells based on TF activity profile (left) and highly variable gene expression profile (right). Meanwhile, clustering based on gene expression revealed that 89 genes exhibited uniquely high expression specifically in Cancer Basal cells, which contributed to the improved separation of these cells from other cell types. b) Bar plot displaying the values of three evaluation indicators for the two clustering results. c) Heatmap plot displaying the activity scores of the top 30 specifically activated TFs in three distinct cancer epithelial cell populations. d) Radar chart showing Gene Ontology (GO) biological process enrichment results for specific activated TFs and their expressed targets in the three cancer epithelial cell populations. Common pathways are depicted in gray text, while cell type‐specific pathways are indicated in text colors corresponding to panel (a). Dot plots illustrating the degree of TF activation that significantly changed as the disease progressed (pathological tumor stage) in the three cancer epithelial cell populations: e) Cancer LumA, f) Cancer LumB, and g) Cancer Basal. BRCA, breast cancer. TNBC, triple‐negative breast cancer.

We reanalyzed an scRNA‐seq dataset of human breast cancer epithelial cells^[^ ^45^ ^]^ (GSE176078), maintaining the original classification of seven cell populations: Cancer Basal, Cancer Her2, Cancer LumA, Cancer LumB, Luminal Progenitors, Mature Lumial, and Myoepithelial (Table S9, Supporting Information). Four of these populations were composed of cancer cells: Cancer LumA (predominantly Estrogen Receptor (ER^+^) clinical subtype, 98.9%), Cancer LumB (mainly ER^+^ clinical subtype, 94.5%), Basal (primarily triple‐negative breast cancer (TNBC) clinical subtype, 96.8%), and Cancer Her2 (present across clinical subtypes, comprising 8.8% ER^+^, 36.1% HER2^+^, and 55.1% TNBC). Our analysis used highly variable TFs (top 500) and genes (top 2000), and found that TF activity outperformed gene expression in distinguishing cell populations, particularly evident in T‐distributed stochastic neighbor embedding (t‐SNE) visualization (**Figure** **4**a; Experimental Section). Assessment metrics consistently demonstrated that TF activity more accurately grouped and separated cell types^[^ ^46^ ^]^ (Figure 4b; Experimental Section; Table S10, Supporting Information). To delve into clinical subtype‐specific regulation, we focused on three cancer cell types (Cancer LumA, Cancer LumB, and Cancer Basal). Differential analysis identified 53, 58, and 65 subtype‐specific activated TFs in Cancer LumA, Cancer LumB, and Cancer Basal, respectively. Among these, there were known regulators such as *HOXB2* ^[^ ^47^ ^]^ and *KLF4* ^[^ ^48^ ^]^ in Cancer LumA, as well as *HMGA1* ^[^ ^49^ ^]^ in Cancer Basal (Figure 4c; Table S11, Supporting Information). Pathway analysis for TFs activated in specific clinical subtypes, along with their targets, revealed common properties associated with malignant proliferation, such as epithelial cell differentiation, regulation of transcription from RNA polymerase II promoter in response to stress, and regulation of cytoplasmic translation pathways. Each subtype also exhibits unique processes. For example, Cancer LumA displayed involvement in the apoptotic signaling pathway, negative regulation of programmed cell death, and response to estrogen.^[^ ^45^ ^]^ Cancer LumB showed an association with incorrect protein metabolic‐related pathways,^[^ ^45^ ^]^ while Cancer Basal exhibited enrichment in Messenger Ribonucleic Acid (mRNA) processing and RNA stability‐related pathways^[^ ^50^ ^]^ (Figure 4d; Table S12, Supporting Information). Furthermore, we identified 8, 22, and 24 Cancer Her2‐specific activated TFs in these three clinical subtypes, respectively (Figure S11a and Table S13, Supporting Information). These TFs were predominantly associated with glycolytic process, protein localization, and pre‐miRNA processing related pathways (Figure S11b and Table S14, Supporting Information). We validated these clinical subtype‐specific TFs using an expression‐based AUCell approach,^[^ ^18^ ^]^ which demonstrated strong consistency with the metaTF results (Figure S11c–e, Supporting Information). To ascertain the effectiveness of metaTF in identifying clinical subtype‐specific activated TFs, we obtained the BRCA RNA‐seq dataset from The Cancer Genome Atlas (TCGA)^[^ ^51^ ^]^ and clinical subtyping based on Berger et al.’s work.^[^ ^52^ ^]^ Subsequently, gene set variation analysis (GSVA)^[^ ^53^ ^]^ revealed significant differences in the activity of 15 out of 53 TFs in LumA, 24 out of 58 TFs in LumB, and 28 out of 65 TFs in Basal, compared to other clinical subtypes (Figure S12a,b,d,f and Table S15, Supporting Information). The gene set enrichment analysis (GSEA) results further confirmed the specificity of these TFs to cancer subtypes (Figure S12c,e,g, Supporting Information). To further validate the effectiveness of subtype‐specific activated TFs identified by metaTF, the potential roles of the top 15 Basal‐activated TFs in the proliferation, migration, and invasion of Basal subtype of breast cancer cells were analyzed. We knockdown the expression of these 15 TFs in two basal breast cancer cell lines (Hs578T and BT549 cells) (Figures S13a and S14a and Table S16, Supporting Information). Among these TFs, *SOX6*, *SOX15*, *CEBPG*, *KLF13*, *NFAT5*, and *SMAD1* were experimentally validated for their ability to affect the migration and invasion of breast cancer cells, the transwell assay results showed that knockdown of these TFs, respectively, significantly decreased the migration (Figures S13b and S14b, Supporting Information) and invasion (Figures S13c and S14c, Supporting Information) of both cancer cells. In addition, we also detected the effects of 15 TFs on the proliferation function of breast cancer cells, among which knockdown of *SOX6*, *SOX15*, or *CEBPG* significantly inhibited the proliferation of Hs578T and BT549 cells, respectively (Figures S13d and S14d, Supporting Information). Furthermore, the functional study demonstrated that these six TFs have no significant impact on the migration of T47D, a luminal A type of breast cancer cell line (Figure S15, Supporting Information). This result substantiates the effectiveness of the subtype‐specific activated transcription factors identified by metaTF. Additionally, to validate whether 6/15 is a remarkable percentage, we randomly selected 15 expressed TFs and performed migration assays in Basal‐like breast cancer cell lines, and showed that only two out of the randomly selected 15 TFs significantly inhibited the migration of BT549 cells (Figure S16a,b, Supporting Information). When incorporating the impact of these TFs on the proliferation of BT549 cells into consideration, none of these 15 randomly selected TFs significantly affect both proliferation and migration of BT549 cells (Figure S16c, Supporting Information). Biological replicate experiments have yielded results that are consistent with the previous conclusions (Figures S17 and S18, Supporting Information). These results suggest that activated TFs identified by metaTF somehow play critical roles in the progression of breast cancer. As cancer progresses and the patient's condition worsens, TF activities change with disease advancement. Analyzing activity dynamics across cancer stages unveiled dramatic changes in TF activity throughout disease progression (Figure 4e–g). For instance, *KDM5B* ^[^ ^54^ ^]^ and *SNAI1* ^[^ ^55^ ^]^ activities exhibited gradually increased in ER^+^ cells, aligning with their known roles in breast cancer metastasis. In contrast, *MYB* activity gradually declined, consistent with its suppressive role in breast cancer metastasis.^[^ ^56^ ^]^ To validate the repeatability of the findings in human breast cancer epithelial cells reported by metaTF, additional data^[^ ^57^ ^]^ (GSE180286) analysis was conducted (Experimental Section). Consistently, metaTF demonstrated superior capability in distinguishing cancer cells from normal cells. Furthermore, through a further analysis, 47, 63, and 57 subtype‐specific activated TFs were identified in Cancer LumA, Cancer LumB, and Cancer Basal, respectively. As depicted in Figure S19 (Supporting Information), there is substantial overlap between these TFs and the previous analysis results. Meanwhile, to demonstrate the broad applicability of metaTF, when applied metaTF to prostate cancer epithelial cells^[^ ^58^ ^]^ (GSE176031), the results also demonstrated that clustering based on TF activity outperformed clustering based on gene expression. Likewise, random forest classification yielded similar outcomes (Experimental Section; Figure S20, Supporting Information). In summary, analyzing TF activity has provided novel insights into the heterogeneity of breast cancer epithelial cells, enhancing our understanding of the regulatory mechanisms driving cancer initiation and progression.

### 2.5 Discovering New Neural‐Regulated T‐Cell Subsets in the Breast Tumor Microenvironment through MetaTF

**Figure 5:**
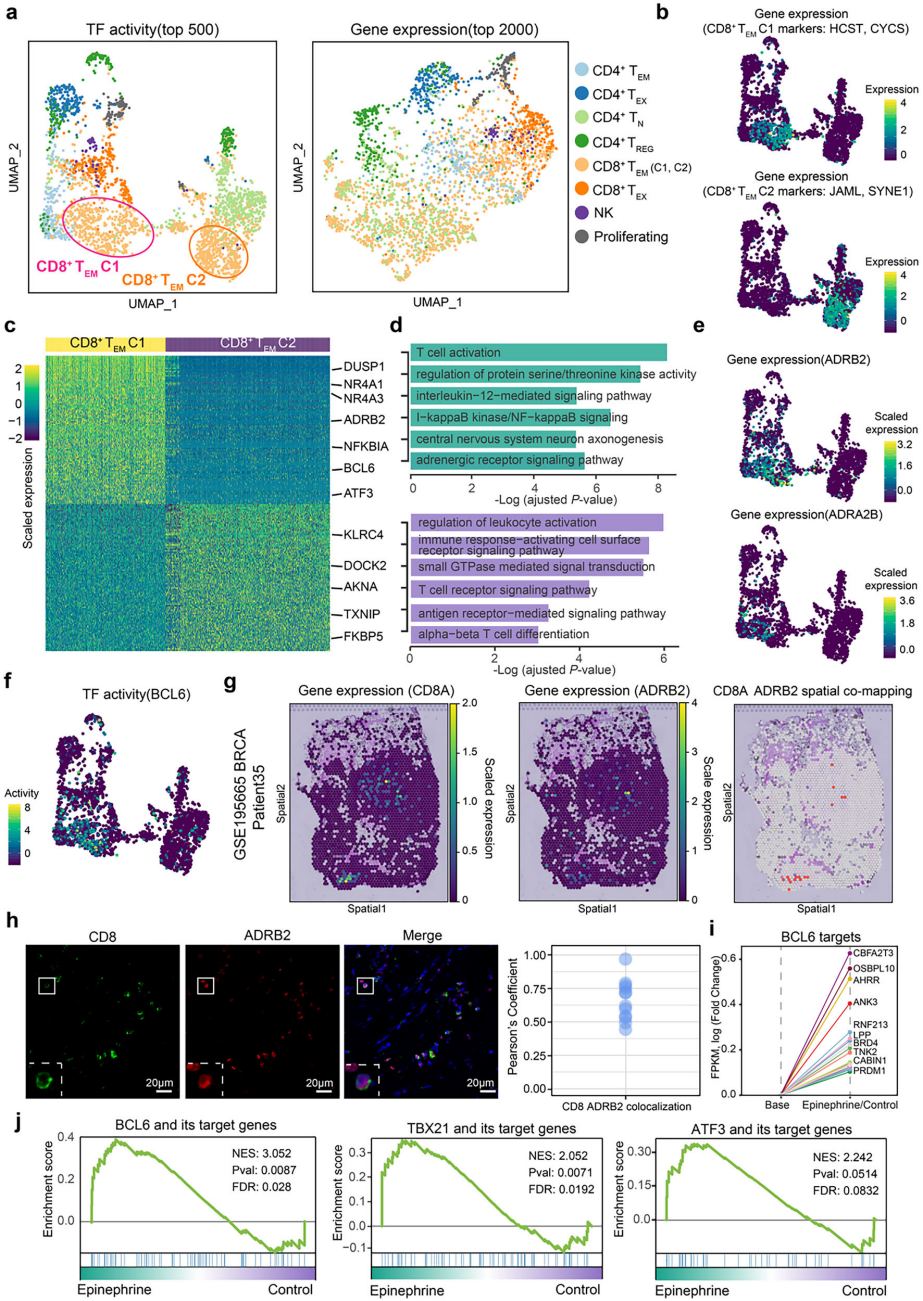
metaTF reveals TF activity heterogeneity in human breast cancer T cells. a) UMAP plots visualizing cluster assignments of human breast cancer T cells based on TF activity profile (left) and highly variable gene expression profile (right). Cells are color‐coded to represent different human breast cancer T‐cell populations, including T_EM_ (effector/memory T cells), T_EX_ (experienced T cells), T_N_ (naive T cells), T_REG_ (regulatory T cells), natural killer (NK) cells), and Proliferating (proliferating T cells). b) UMAP plot of CD8^+^ T_EM_ C1 and C2 marker genes expression. c) Heatmap plot showing the expression of the top 50 differentially expressed genes between CD8^+^ T_EM_ C1 and C2 cell populations. d) GO biological process enrichment results for the differentially expressed genes between CD8^+^ T_EM_ C1 (upper panel) and C2 (lower panel) cell populations respectively. e) Dot plot showing the significant expression of β_2_‐adrenergic receptor (*ADRB2*) and adrenoceptor alpha 2B (*ADRA2B*) primarily in the CD8^+^ T_EM_ C1 cell population. f) Dot plot indicating that *BCL6* was specifically activated in the CD8^+^ T_EM_ C1 cell population. g) Spatial co‐mapping of *CD8* and *ADRB2* gene expression by spatial transcriptomics of a sample from a breast cancer patient recorded in a public dataset (GSE195665, patient35, right panel). The left and middle panels depict expression levels in fresh frozen sections. h) Expression of CD8 and ADRB2 in breast cancer tissue, and the colocalization (Pearson's coefficient) between CD8 and ADRB2 is shown, *n* = 12. i) Upregulated targets of *BCL6* in the epinephrine treatment group compared to the control group from in vitro cytological experiments. j) In vitro cytological experiments confirmed that candidate TFs and their targets are highly expressed in the epinephrine‐treated group through GSEA prerank analysis.

The intricate interplay between the immune system and cancer has become a prominent focus in cancer biology research. Immune cells possess the ability to recognize and eliminate nascent tumor cells during immune surveillance. Paradoxically, they can also act as supporters of tumor growth and progression through immunoediting. Excitingly, clinical advancements in immunosuppressive therapies and cellular immunotherapies have demonstrated promise in targeting these dual actions of immune cells within the tumor microenvironment.^[^ ^59^, ^60^, ^61^ ^]^ Nonetheless, our understanding of the underlying transcriptional mechanisms governing pro‐ and antitumor immunity remains incomplete.^[^ ^62^ ^]^ To shed light on the transcriptional regulation of T‐cell states within the tumor microenvironment, we applied metaTF to analyze two scRNA‐seq concerning breast cancer datasets.^[^ ^63^ ^]^ Initial clustering based on highly variable (top 2000) gene expression profile indicated that there was no clear separation of T‐cell subpopulations (**Figure** **5**a, right). However, when using TF activity (top 500) profile, a pronounced separation of T‐cell subpopulations was presented, particularly in the case of CD8^+^ T‐effector memory (T_EM_) cells, which were divided into two distinct clusters, denoted as CD8^+^ T_EM_ C1 and C2 (Figure 5a, left). The classification accuracy for the newly identified CD8^+^ T_EM_ C1 and C2 was confirmed through the expression of their respective marker genes (Figure 5b). Differential analysis revealed 268 genes that were upregulated in CD8^+^ T_EM_ C1 and 153 genes in C2 (Figure 5c; Table S17, Supporting Information). C1 displayed higher expression of activation/proliferation markers, like *NFKBIA* ^[^ ^64^ ^]^ as well as genes related to CD8^+^ T‐cell response to acute infection, memory generation, and activation stress.^[^ ^65^, ^66^ ^]^ In contrast, C2 displayed elevated expression of *DOCK2*, which has the potential to impair the immune function of CD8^+^ T cells.^[^ ^67^ ^]^ Intriguingly, pathway analysis revealed that C1 was enriched in functions associated with T‐cell activation, regulation of protein serine/threonine kinase activity, interleukin‐12‐mediated signaling, and notably, neuronal/synaptic processes such as axonogenesis (Figure 5d, upper panel; Table S18, Supporting Information). Given emerging evidence indicating the influence of nerves within the tumor microenvironment on cancer progression,^[^ ^68^, ^69^ ^]^ we first confirmed the protein level of nerve fibers marker (antineurofilament heavy,^[^ ^70^ ^]^ NF) in breast cancer tissue (Figure S21d, Supporting Information), subsequently examined the neuroreceptor expression level in scRNA‐seq data and discovered that C1 specifically expressed adrenergic receptors (ARs) *ADRB2* and *ADRA2B* (Figure 5e). By analyzing publicly available spatial transcriptomics data, we confirmed co‐expression of *CD8A* and *ADRB2* within breast tumors^[^ ^41^, ^71^ ^]^ (Figure 5g; Figure S22, Supporting Information). Validation through immunofluorescence in breast cancer patient samples confirmed the expression of ADRB2 on tumor‐infiltrating T cells (Figure 5h; Figure S21i and Table S19, Supporting Information). To further validate these findings, we isolated tumor‐infiltrating lymphoid T cells (CD8^+^ cells) from breast cancer patients using flow cytometry (Figure S21e,f, Supporting Information). Subsequently, we treated these T cells with epinephrine^[^ ^72^ ^]^ (an adrenergic receptor agonist, 0.1 ng mL^−1^) to activate neurotransmitter receptors and assessed downstream TF expression and activity through RNA sequencing (Figure S21g, Supporting Information). After performing GSEA analysis, the results showed that *BCL6*, *TBX21*, and *ATF3*, along with their respective downstream targets, exhibited significant activation in the epinephrine treatment group (Figure 5i,j; Figure S21h and Table S20, Supporting Information). These results are consistent with the findings of metaTF activity analysis in CD8^+^ T_EM_ C1 (Figure 5f; Figure S21a–c, Supporting Information). This suggests that CD8^+^ T_EM_ C1 cells respond to neuro‐related signals by activating these transcription factors and their downstream targets. To examine the consistency of our findings in other breast cancer dataset, we reanalyzed an scRNA‐seq dataset of human breast cancer T cells^[^ ^73^ ^]^ (GSE110686), maintaining the original classification of 11 cell populations. Similarly, we employed metaTF to dissect the heterogeneity of these 11 cell clusters, and the results aligned with previous findings. CD8^+^ T_EM_ cells were stratified into two distinct clusters, with one subset specifically expressing adrenergic receptor ADRB2, while the other subset did not (Figure S23a, Supporting Information). Differential TF activation analysis revealed that BCL6 exhibited specificity in activation within ADRB2^+^ CD8^+^ T_EM_ cells, consistent with earlier conclusions. However, slightly divergent from previous findings, *TBX21* and *ATF3* were not exclusively activated within ADRB2^+^ CD8^+^ T_EM_ cells (Figure S23b,c, Supporting Information). In summary, our analysis confirms the existence of a previously unrecognized subset of CD8^+^ T_EM_ cells with specialized functionality, characterized by the expression of neural receptors.

## 3 Conclusion

We introduce here metaTF, a computational framework designed for analyzing TF activity from scRNA‐seq data. MetaTF offers a robust assessment of TF activities, independent of experimental methods, by integrating prior TF–target networks and expression‐based gene regulatory networks. The novelties of metaTF encompass several key aspects. First, metaTF starts by building comprehensive prior networks that compile TF–target interactions from various sources, including curated databases, ChIP‐seq analyses, and experimental perturbations (e.g., TF knockout/knockdown studies). This approach incorporates both strongly and weakly supported TF–target relationships, mitigates the biases associated with relying on a single data modality. By integrating diverse dimensions of TF biology, such as chromatin context and regulatory dynamics, metaTF provides a more holistic view of TF–target interactions that surpasses conventional methodologies dependent solely on structural motifs or DNA‐binding domains. Second, one of the critical advancements of metaTF is its ability to integrate and analyze multiple types of data, a capability that previous algorithms lacked. MetaTF employs a biologically driven network integration technique to merge the dynamic regulatory networks derived from scRNA‐seq data with the static prior networks. This integration enhances the accuracy of TF regulation portrayal by incorporating real‐time, cell‐specific regulatory interactions. This comprehensive integration approach enables metaTF to capture both canonical and cell‐specific regulatory events, providing deeper insights into gene regulatory networks. Moreover, by analyzing data from CD8^+^ cytotoxic T‐cell differentiation, metaTF has not only demonstrated exceptional performance in analyzing gene regulatory networks but has also shown its ability to predict the effects of TF perturbations with remarkable accuracy, emphasizing that it provides an effective and applicable solution for quantitative analysis of single‐cell‐based CRISPR screening data. Certainly, limitations include reduced performance with higher dropout rates and a focus on inferring activated rather than repressed regulatory edges. Potential solutions involve assigning greater weight to prior knowledge and utilizing methods like PCOR or scLink, which support the calculation of negative edges. Although metaTF demonstrated superior performance over SCENIC and DoRothEA in identifying perturbation‐affected target genes for most TFs, discrepancies arose in regulon composition, particularly for TFs like *FOXQ1*, where nondifferentially expressed targets (e.g., *RBBP5*,^[^ ^74^ ^]^ *SOX12*,^[^ ^75^ ^]^ and *SIRT1* ^[^ ^76^ ^]^) reduced metaTF prediction accuracy (Figure 2a; Figure S24, Supporting Information). These targets likely reflect regulatory complexities, such as epigenetic modifications or super enhancers, highlighting the necessity for metaTF to incorporate multiomics data to achieve more robust inference of TF activity. Additionally, we are actively compiling and curating similar TF regulatory networks for other model organisms, such as Drosophila^[^ ^77^ ^]^ and zebra fish,^[^ ^78^ ^]^ to expand the application of metaTF.

In our study, we used 10× Genomics scRNA‐seq data from melanoma cultures to evaluate how low coverage affects metaTF performance. We simulated synthetic dropout in cells treated with *SOX10* knockdown or nontargeting Small Interfering Ribonucleic Acid (siRNA). As dropout rates increased, both the number of detected genes and metaTF performance decreased. When the median number of detected genes per cell fell below 2500, metaTF's predictive performance markedly declined, and its accuracy was around 50% when this number dropped below 2000. This highlights the challenges of low‐coverage data and suggests that imputation methods like MAGIC^[^ ^79^ ^]^ and DeepImpute^[^ ^80^, ^81^ ^]^ could be useful for improving data quality and metaTF performance in such scenarios.

Cancer represents an intricate and exceedingly heterogeneous class of diseases, and this heterogeneity presents itself in various manifestations. First, patients with the same type of cancer often exhibit varying prognostic outcomes and responses to treatment. Second, within the tumor microenvironment of an individual cancer patient, both the composition and biological behaviors of cancer cells and stromal cells undergo dynamic changes as the disease progresses. TFs play pivotal roles in orchestrating how cancer cells respond to changes in their microenvironment. Notably, the solid tumor microenvironment is usually characterized by hypoxia and cancer‐associated inflammation, as elucidated in previous studies.^[^ ^82^, ^83^, ^84^ ^]^ Hypoxia‐induced factor 1α (HIF1α) is a key TF that mediates cancer cells' adaptation to hypoxia stimuli. Interestingly, it is noted that the mRNA level of HIF1α remains largely unchanged during hypoxia compared to normoxia. In contrast, HIF1α protein is hydroxylated and degraded under normoxia, but rapidly accumulates and forms a heterodimer with HIF1β to regulate gene expression in response to the hypoxic microenvironment.^[^ ^85^ ^]^ When stimulated by inflammatory mediators or cytokines secreted by immune cells in the tumor microenvironment, the inflammation‐associated TF Nuclear Factor kappa‐B (NF‐κB) in cancer cells is activated, leading to the upregulation of genes that regulate cancer proliferation and metastasis. However, the activation of NF‐κB occurs through its translocation from the cytoplasm to the nucleus, rather than an increase in its mRNA or protein levels.^[^ ^86^ ^]^ These observations underscore the importance of investigating TF activities to understand the mechanisms driving cancer progression. In this study, we utilized metaTF to discern subtype‐specific activated TFs within various molecular subtypes of breast cancer. Our findings unveiled a plethora of subtype‐specific TFs with heightened activities in breast cancer. Delving deeper into these hitherto overlooked TFs will not only shed light on the regulatory mechanisms underlying diverse clinical subtypes and stages of breast cancer but also provide promising targets for therapeutic interventions in the management of breast cancer.

Additionally, it is noteworthy that in addition to the inherent heterogeneity observed in cancer cells, immune cells infiltrating the tumor microenvironment also exhibit dynamic and diverse characteristics. These tumor‐infiltrated immune cells can lose their antitumor efficacy, and, in some cases, even adopt procancer functions within the tumor microenvironment.^[^ ^86^, ^87^, ^88^, ^89^ ^]^ In light of these considerations, we propose that delineating cell identities based on TF activity profiles offers distinct advantages over conventional strategies that rely on gene expression profiles when investigating the functional roles of immune cells. Therefore, utilizing metaTF can facilitate the discovery of new subpopulations, essential subtype‐specific regulators, and TF‐to‐TF regulatory circuitry. In this study, metaTF uncovered a previously unrecognized subset of CD8^+^ T_EM_ cells characterized by the expression of neural receptors within the microenvironment of breast cancer. These cells exhibit distinct transcriptional profiles and respond to neuro‐related signals, suggesting a potential role for neural signaling in modulating T‐cell function within breast cancer. Certainly, the exact mechanistic details of how these TFs influence immune responses and tumor behavior are not yet fully elucidated. Further investigations into the downstream effects and signaling pathways involved in CD8^+^ T_EM_ C1 cells would provide a deeper understanding of the mechanisms at play. Moreover, exploring clinical correlations and outcomes related to the presence and function of CD8^+^ T_EM_ C1 cells in breast cancer patients can enhance the clinical relevance of these findings. In addition, longitudinal studies tracking the dynamics of CD8^+^ T_EM_ C1 cells over time during cancer progression and in response to treatments would provide insights into their role in disease progression and therapy resistance.

In this study, metaTF uncovered a previously unrecognized subset of CD8^+^ T_EM_ cells characterized by the expression of neural receptors within the microenvironment of breast cancer. These cells exhibit distinct transcriptional profiles and respond to neuro‐related signals, suggesting a potential role for neural signaling in modulating T‐cell function within breast cancer. Catecholamines bind to adrenergic receptors on immune cells, including T cells, initiating complex and diverse signaling pathways. Continuous stimulation of nor‐adrenaline in the tumor microenvironment triggers the canonical pathway of β2‐ARs expressed in T lymphocytes.^[^ ^90^ ^]^ β2‐AR triggers a cascade involving G protein dissociation, Cyclic Adenosine Monophosphate (cAMP) production, and Protein Kinase A (PKA) activation, which subsequently modulates key signaling molecules like Lck and ZAP70. This ultimately impacts T‐cell activation, proliferation, and function.^[^ ^91^ ^]^ On the other hand, β2‐AR upregulates the expression of checkpoint receptor PD‐1, which further inhibits CD28‐mediated T‐cell activation signaling.^[^ ^92^ ^]^ Additionally, the adrenergic system can modulate T‐cell apoptosis in a PKA‐independent manner by engaging the Src family tyrosine kinase Lck.^[^ ^93^ ^]^ These studies suggest that β2‐AR activation in T cells generally suppresses their immunosurveillance functions. Our study using metaTF on scRNA‐seq breast cancer datasets found that CD8^+^ T_EM_ cells could be divided into two clusters based on TF activity, with the ADRB2^+^ CD8^+^ T_EM_ subset showing higher expression of activation and proliferation markers and genes related to immune responses, and validation experiments demonstrated that ADRB2^+^ CD8^+^ T_EM_ cells respond to neuro‐related signals by activating key transcription factors such as *BCL6*, *TBX21*, and *ATF3*, along with their downstream targets. Considering that *BCL6* has been recognized to play important roles in generation and proliferation of CD8^+^ memory T cells,^[^ ^94^ ^]^ *TBX21* may be necessary for the terminal differentiation of CD8^+^ T cells,^[^ ^95^ ^]^ and *ATF3* may promote the infiltration of functional CD8^+^ T cells,^[^ ^96^ ^]^ thus we believed that we identified a novel subtype of CD8^+^ T cells and activation of β2‐AR signaling by epinephrine may activate the cytotoxic activity of this type of CD8^+^ T cells. Certainly, the exact mechanistic details of how these TFs influence immune responses and tumor behavior are not yet fully elucidated. Further investigations into the downstream effects and signaling pathways involved in CD8^+^ T_EM_ C1 cells would provide a deeper understanding of the mechanisms at play.

In summary, metaTF provides an integrated framework to reconstruct GRNs and analyze TF activity from scRNA‐seq data. This framework will aid biologists in elucidating the transcriptional regulation of cell identities, states, and functions. Continued advancements in network inference and activity analysis methods will further strengthen the interpretation of single‐cell genomics data.

## 4 Experimental Section

### The MetaTF Workflow

The metaTF workflow consists of five main steps: data preprocessing, GRN inference, network integration, regulon identification, and TF activity estimation. The standard protocol is as follows:

1. *Data Import*: The raw count matrix from multiple sources was summarized into a SingleCellExperiment object, subsequently, log2 normalization and filtration were performed to remove genes expressed in fewer than five cells usually.
2. *GRN Inference*: PUIC, an entropy‐based method, was used to infer regulator–target relationships by quantifying the unique contribution from the mutual information between each gene triplet.^[^ ^97^ ^]^ Suppose a target gene T and a vector of regulator genes are given. Then, the relationships between *R* and *T* can be estimated with MI. However, one target gene can be simultaneously regulated by multiple regulators, indicating that the MI between every two *R* and *T* pairs may be dependent. Therefore, it was needed to decompose the partial information that was uniquely contributed by the indicated regulator. Here, the non‐negative decomposition of multivariate information (NDMI) algorithm^[^ ^98^ ^]^ was utilized to decompose the MI into unique (Unq), redundant (Rdn), and synergistic (Syn) information. Specifically, the simplest form of NDMI with only three variables was considered: one target gene T, and two regulator genes {*R*_1_, *R*_2_}. The MI *I*(*T*;  *R*_1_, *R*_2_) can be decomposed as
(1) $$\begin{array}{c} I\left(T;\;R_{1},R_{2}\right)=\mathrm{Unq}\left(T;\;R_{1}\right)+\mathrm{Unq}\left(T;\;R_{2}\right)+\mathrm{Rdn}\left(T;\;R_{1},R_{2}\right)\\ +\mathrm{Syn}\left(T;\;R_{1},R_{2}\right) \end{array}$$

where the MI between *T* and *R*_1_ is

(2) $$I\left(T;\;R_{1}\right)=\mathrm{Unq}\left(T;\;R_{1}\right)+\mathrm{Rdn}\left(T;\;R_{1},R_{2}\right)$$

And the same with *R*_2_

(3) $$I\left(T;\;R_{2}\right)=\mathrm{Unq}\left(T;\;R_{2}\right)+\mathrm{Rdn}\left(T;\;R_{1},R_{2}\right)$$

Here, a gene regulatory network was considered with *n* genes. Given a pair of genes *R* and *T*, there are *n* − 2 possible partners $R_{2}=P=\left\{P_{1},P_{2},\text{…},P_{n-2}\right\}$ involved in the triple gene pairs. Since the MI between *R* and *T* is unaffected by any choice of a third partner $P=\{P_{1},P_{2},\text{…},P_{n-2}\}$, the unique information Unq(*T*;  *R*) could accurately measure the individual contribution of *R* to *T*. Therefore, the total PUIC of all *n* − 2 triple gene pairs was used to estimate the importance of a regulator *R* to a target *T* as

(4) $$\mathrm{PUC}\left(R;\;T\right)=\sum_{i=1}^{n-2}\frac{\mathrm{Un}q_{p_{i}}\left(R;\;T\right)}{I\left(R;\;T\right)}$$

To calculate the unique information between regulator *R* and target *T* within each triple gene pair, the redundancy should first be measured by specific information *I*_spec_(*T*  =  *t*;  *R*) as

(5) $$I_{\mathrm{spec}}\left(T\;=\;t;\;R\right)\;=\;\sum_{r\in R}p\left(r|t\right)\left[\log\frac{1}{p\left(t\right)}-\log\frac{1}{p\left(t|r\right)}\right]$$

where $T=\{t_{1},t_{2},\text{…},t_{k}\}$ and $R=\{r_{1},r_{2},\text{…},r_{k}\}$ are nonempty subsets of target *T* and regulator *R*, respectively. In the context of gene expression data, *t* and *r* represent the discrete bins in which a gene's expression value fell in. In this research, the number of bins (*k*) of each gene was set as the nearest integer of the square root of the cell number. Then, the redundancy information of the triple gene pair was calculated with one regulator *R*, one partner *P*, and one target *T* by comparing the specific information from each bin within {*R*, *P*} about each bin *t* of target *T* as

(6) $$\mathrm{Rdn}\left(T;\;\left\{R,P\right\}\right)=\sum_{i\in T}p\left(t\right)\min_{\left\{R,P\right\}}I_{\mathrm{spe}}\left(T=t;\left\{R,P\right\}\right)$$

Next, the MI between regulator *R* and target *T* was be calculated as

(7) $$I\left(R;\;T\right)=H\left(R\right)+H\left(T\right)-H\left(R,T\right)$$

*H*(*R*) or *H*(*T*) represents the entropy that quantifies the uncertainty of the gene expression level as

(8) $$H\left(R\right)=-\sum_{r\in R}p\left(r\right)\log\;p\left(r\right)$$

To calculate the frequency *r* in each bin within {*R*}, the gene expression was first dispersed into $k=\sqrt{m}$ bins according to the range of expression levels, while *m* represents the total number of cells and *r* represents the number of cells falling into each bin.

It was noticed that the MI was symmetric but the redundancy was asymmetric, so the PUC score was an asymmetric measure that was suitable for transcriptional regulation since this is a directional process. However, for a pair of genes that exist both in the regulator and target genesets, the final score was calculated as the average PUC in both directions, as follows

(9) $$\mathrm{PU}C_{R,T}=\left\{\begin{array}{c} \frac{\mathrm{PUC}\left(R;T\right)+\mathrm{PUC}\left(T;R\right)}{2},R\in T\\ PUC\left(R;T\right),R\notin \;T \end{array}\right.$$

The final PUC score for each gene pair was further normalized based on its average cumulative distribution in the regulator and its target set as

(10) $$W_{R,T}=\frac{F_{R}\left(\mathrm{PU}C_{R,T}\right)+F_{T}\left(\mathrm{PU}C_{R,T}\right)}{2}$$

where the *F_R_* (PUC_*R*,*T*_) is the cumulative distribution of all PUC scores involving the regulator *R*, *F_T_* (PUC_*R*,*T*_) is the cumulative distribution of all PUC scores involving the target *T*, and *W*_*R*,*T*_ represents the weighted regulatory relationships between regulators and targets. The weighted GRN was constructed for subsequent analysis. Here, classic information‐theoretic approaches use the on‐off binary approximation, which is appropriate for scRNA‐seq data given that it mostly yields data about transcript presence or absence, on–off binary approximation‐based methods^[^ ^99^, ^100^ ^]^ were also incorporated into the single‐cell GRN inference framework.

1. *Prior‐Network Construction*: The TF–target relationships inferred from PUIC algorithm are only based on gene expression, they might include many false‐positive targets. To reduce the effect of the false‐positive connections, a prior TF–target network (knowledge‐based information) was constructed. TFs were obtained from the HumanTFs website^[^ ^101^ ^]^ (http://humantfs.ccbr.utoronto.ca/) and constructed the human prior TF–target networks based on four main resources: manually curated datasets, ChIP‐seq binding datasets, TF knockout experiment, and tissue‐specific transcriptional regulatory networks (TRNs). 1) The manually curated TF–target relationships were collected from the following databases and literature: TRRUST,^[^ ^102^ ^]^ INTACT,^[^ ^103^ ^]^ TRANSFAC,^[^ ^104^ ^]^ FANTOM4,^[^ ^105^ ^]^ TFe,^[^ ^106^ ^]^ MSIGDB,^[^ ^107^ ^]^ PAZAR,^[^ ^108^ ^]^ TFactS,^[^ ^109^ ^]^ HTRIDB,^[^ ^110^ ^]^ TRED,^[^ ^111^ ^]^ TRRD,^[^ ^112^ ^]^ and Neph2012.^[^ ^113^ ^]^ 2) The ChIP‐seq associated TF–target networks were collected from two ChIP‐seq related databases (ChEA^[^ ^114^ ^]^ and ENCODE^[^ ^115^ ^]^) and two motif‐based databases (HOCOMOCO^[^ ^116^ ^]^ and JASPAR^[^ ^117^ ^]^) by predicting the TF binding sites. 3) The TF knockout/knockdown information was downloaded from the KnockTF^[^ ^112^ ^]^ database. First, the datasets that knockout/knockdown TFs were significantly downregulated (*P* < 0.05 and log‐transformed fold changes (logFC) < −1) compared to the control group were only retained. Then, the differentially expressed genes (*P* < 0.05 and |logFC| > 1) were regarded as targets for the knockout/knockdown TF, and the weight of interactions was calculated as log_10_ (*P*). 4) Tissue‐specific and blood‐specific TF‐target networks were constructed based on 32 tissue datasets^[^ ^118^ ^]^ and six blood datasets (https://zenodo.org/record/8355476), respectively.
2. *Regulon Identification*: First, the prior TF–target networks and the weighted GRN were integrated using the following equation
(11) $$W_{R,T}=\frac{1}{1+e^{-\left(a^{0}+\sum_{i=1}^{n}a^{i}w_{R,T}^{i}\right)}}$$

1. *TF Activity Estimation*: The above filtered regulons were used to calculate the relative enrichment score (VIPER^[^ ^17^ ^]^ by default) against the log2‐normalized expression data and integrate the TF–target networks. VIPER is a statistical framework that was developed to estimate protein activity from gene expression data using enriched regulon analysis performed by the algorithm aREA. It requires information about interactions between a protein and its transcriptional targets and the likelihood of their interaction. Here, the log2 normalized expression data and integrated TF target network were inputted to VIPER to generate the final TF activity matrix. Moreover, several functions were added to the metaTF package for statistical analysis and visualization, such as cell‐type‐specific activated TF identification, regulon pathway enrichment analysis, and data visual aids (dot plot, Radviz plot, Sankey diagram, and heatmap).

where *n* represents the number of sources, *a^i^* controls the importance of the source *i* ^th^, *a* ^0^ is a constant parameter, and $w_{R,T}^{i}$ denotes the weight of the TF–target in source *i* ^th^. Moreover, the mouse prior TF–target networks in the current research were transformed from the human networks using orthologous genes. Then, a normal distribution was fitted to the weights of targets for each TF in the integrated TF–target network, and the truncation value *q* = 0.01 was used to infer the threshold for these targets. For each TF, only targets with weights greater than the threshold were retained as a gene list and inputted together with the previously inferred GRN into the fgseaMultilevel method (fgsea, https://github.com/ctlab/fgsea/) to do preranked gene set enrichment analysis. The leading edge of each enrichment result was considered as positively regulated targets for each TF. Finally, TFs and their positively regulated targets were identified as regulons.

### Data Processing for Prior‐Network Construction

The files “trrust_rawdata.human.tsv” and “edge.GoldStd_TF.tbl.txt” were downloaded from TRRUST and FANTOM4, respectively. From INTACT, a curated dataset, specifically focusing on human protein–DNA interactions, was acquired. From TFe, “Targets” and “interaction” were taken as input content and download relevant data. From TFactS, the “Catalogues.xls” file, which only preserves the interaction pairs of human species and contains relevant literature, was downloaded. From HTRIDB, only the results detected by small and medium‐sized technologies such as chromatin immunoprecipitation and linker chromatin immunoprecipitation were retained. For TRED, the BiPAX file was downloaded from RegNetwork and relevant TF interaction information was extracted. For TRRD, it was ultimately extracted from the “Catalogues.xls” file of TFactS. From MSIGDB, the regulatory target gene set collection was downloaded. For Neph2012, it was downloaded directly from its R package. From TRANSFAC, the transcription factor regulatory gene sets were downloaded. For PAZAR, it was downloaded from the ORegAnno database. Finally, all the interaction relationships together without redundancy were integrated.

The integration of the prior associations between transcription factors and target genes from the aforementioned sources involved the utilization of MI scores for weighting. These scores, which were normalized weighted counts, take into account independent interaction evidence and relevant experimental methods to quantify the relationships accurately

(12) $$\mathrm{Weigh}t_{\mathrm{MI}}=\frac{K_{p}\times S_{p}\left(n\right)+K_{m}\times S_{m}\left(cv\right)+K_{t}\times S_{t}\left(cv\right)}{K_{p}+K_{m}+K_{t}}$$

Here, the weights in three dimensions, namely number of publications, experimental detection method, and interaction type, are denoted as *K*_p_,*K*_m,_ and *K*_t_ respectively. Experimental methods are typically classified into three groups: small and mid‐scale techniques such as chromatin immunoprecipitation, concatenate chromatin immunoprecipitation, Cytosine‐phosphate‐Guanine (CpG) chromatin immunoprecipitation, DNA affinity chromatography, DNA affinity precipitation assay, DNase I footprinting, electrophoretic mobility shift assay, southwestern blotting, streptavidin chromatin immunoprecipitation, surface plasmon resonance, yeast one‐hybrid assay; high‐throughput techniques like ChIP‐chip or ChIP‐seq, and unknown. Interaction types include physical interaction, direct interaction, interaction, activation, inhibition, and unknown.

The merged ChIP‐seq binding peaks were obtained from ReMap. Notably, only regulatory proteins excluding transcription factors were considered in this analysis. Using the bedtools closest function, each binding site was matched with the nearest transcription start site (TSS) for every TF. In cases where genes had multiple transcripts, the closest binding site–transcript pair was selected. This enabled the assignment of each binding site to a gene, allowing for the possibility of a gene having no or multiple binding sites for a given TF. Furthermore, a score ranging from 0 to 1 was assigned to each binding site–gene pair based on the distance between the binding site and the TSS

(13) $$\mathrm{ChIp}\left(\mathrm{TF}-G\right)=\sum_{i}e^{-\frac{d_{i}}{k\times D_{\mathrm{TF}}}}$$

Here *D*_TF_ stands for the median distance separating the TSS and binding sites associated with all TFs, whereas *d_i_* refers to the distance between the binding site within a TF and the TSS of the gene g. The score attributed to a TF target is the sum of the weights assigned to all binding sites of that TF along with the TSS of the target.

The analysis involves scanning TFBS in the promoter sequences of each transcript using the human genome reference sequence (GRCh38 version), encompassing 1000 bp upstream (5′ end) and 200 bp downstream (3′ end). Position Weight Matrix (PWMs were sourced from the HOCOMOCO and JASPAR repositories. Employing the Find Individual Motif Occurrences (FIMO) tool in the Multiple EM for Motif Elicitation (MEME) software suite (MEME's FIMO) motif discovery tool with default parameters, putative TFBS were identified in the promoters, focusing on FIMO predictions with a *P*‐value of <0.0001 and removing duplicate matches. It was then proceeded with the annotation of conservation and epigenetic regulatory properties related to TFBS. Essential phase Cons and phyloP scores were sourced from the CellBase database for this purpose. The final weight calculation involved multiplying the phasCons score by the phyloP score (with −log(*P*) value)) while taking the square root into account.

### Performance of GRN Inference Methods

To evaluate the performance of GRN inference methods on single‐cell data, CROP‐seq data of HEK293T cells were downloaded from the Gene Expression Omnibus (GEO, GSE92872^[^ ^36^ ^]^). CROP‐seq enables pooled CRISPR knockout screening of several TFs simultaneously. Ideally, it was considered that the targets of the TFs were activated in wild‐type (WT) cells but not in perturbed cells. To ensure successful perturbations in the CROP‐seq experiments, samples were retained only when targeted TF was significantly downregulated in perturbed cells. Significant differential expression between stimulated and unstimulated cells was determined using a two‐tailed *T‐*test, and only the samples where the target TF was significantly downregulated (*P* < 0.05 and logFC < −1) were retained. A total of 1143 cells including 14 target TFs were retained for further evaluation (Figure S4f, Supporting Information). Next, genes expressed in <10 cells were filtered out. Three different methods, PUIC, GENIE3,^[^ ^27^ ^]^ and PCOR,^[^ ^28^ ^]^ were used for GRN inference. For GENIE3, the above 14 target TFs were only used as input regulators considering the computational time cost. To evaluate these three methods, the targets of each TF were first pre‐ranked according to their edge weights, and then the area under the recovery curve (AUC) was calculated using independent gene sets from DoRothEA^[^ ^15^ ^]^ (ABCDE) as the ground truth. The AUC value of each TF reflects the enrichment of its targets in inferred GRNs. Additionally, to evaluate computational time consumption, expression data were simulated by subsampling different numbers of genes (100, 200, 400, 600, 800, and 1000) within the 1143 cells. The running time was then calculated for GRN inference steps using each simulated expression matrix. The results showed that PUIC outperformed the two other methods in over half of the TFs (Figure 2f). Meanwhile, PUIC is much more efficient than GENIE3 (Figure S4h, Supporting Information).

### Performance of the Integrated TF–Target Network

To evaluate the performance of regulons from different sources, regulons were collected from the above resources in multiple ways: downloaded from DoRothEA (DoRothEA regulons), generated from RcisTarget (RcisTarget regulons), prior‐network and integrated TF‐target network from prior network and the weighted GRN. For DoRothEA regulons, only human regulons named “dorothea_hs” were used. RcisTarget regulons were obtained using the simplified SCENIC workflow described above. To generate GRN‐based regulons, GRNs were first inferred from single‐cell CRISPRi data using the PUIC method with all of the genes as input, and then ARACNE^[^ ^119^ ^]^ was used to remove the weakest connections within the GRNs to generate regulons. Integrated regulons were generated by combining the prior pan‐tissue network and GRNs inferred from single‐cell CRISPRi data of human ESCs.^[^ ^31^ ^]^ Only 40 TFs were retained, corresponding to TFs perturbed in the CRISPRi experiment. TF activities for the above TFs were estimated using the standard metaTF pipeline. Performance was evaluated by calculating AUC using the TF activities estimated as described above. Paired sample *T‐*tests were performed on the AUC values to determine the difference between any two groups of regulons (Figure S4j, Supporting Information). The results showed that the integrated regulons performed better (an average AUROC of 0.62) than the regulons from other sources (Figure S4i, Supporting Information). It was also noticed that the regulons directly downloaded from the database without considering the expression data only performed slightly better than random levels (an average AUROC of 0.52).

### Performance of MetaTF

To evaluate the performance of metaTF on TF activity estimation, the activity scores were transformed into a binary setup based on the perturbation type of the experiment (knockout/overexpression). For knockout experiments, cells with targeted TF knockout were labeled as the negative class, while control cells were labeled as the positive class. For overexpression experiments, the class labels were reversed. The receiver operating characteristic (ROC) and AUC analyses were then performed using the R package ROCR^[^ ^120^ ^]^ (version 1.0‐11). Using the positive and negative classes, the true positive rate (TPR) and false‐positive rate (FPR) were calculated at different thresholds of TF activity to generate the ROC curves (Figure S1c, Supporting Information). Three commonly used single‐sample activity estimation methods including VIPER, ssGSEA, and AUCell, were benchmarked using the same integrated regulons. It was found that both VIPER and ssGSEA showed significantly higher performance than AUCell (Figure S4k, Supporting Information).

### Implementation of Simplified SCENIC Workflow

To efficiently infer the GRNs using the SCENIC^[^ ^18^ ^]^ workflow, a protocol that contains the main SCENIC steps was implemented. In the simplified SCENIC workflow, GENIE3 (version 1.14.0) was first used for GRN reconstruction with potential TFs. Next, the targets of each TF were preranked based on their edge weights in the GRNs, and the top‐ranked (e.g., 5%) targets of each TF were selected as putative regulons. The putative regulons were further trimmed using the R package RcisTarget^[^ ^121^ ^]^ (version 1.12.0) combined with *cis*‐regulatory DNA‐motif information downloaded from https://resources.aertslab.org/cistarget/. By default, the motif database and TF annotation used were from version 9 of cistarget (https://resources.aertslab.org/cistarget/, hg38 and mm10, with a distance 500 bp Up 100 Dw for humans and mice, respectively). All enriched genes were retained only if the TF of the putative regulon was identified as either high or low confidence. Then, the enriched targets from different motifs of the same TF were merged as the final regulons. The TF activity was estimated using the R package AUCell (version 1.14.0) with the final regulons against the log2‐normalized expression data. Finally, the above steps were summarized and implemented as a function named “runSCENIC” in the R package metaTF.

### Preprocessing of Single‐Cell CRISPRi Data

To evaluate the performance of TF regulon analysis methods, scRNA‐seq‐based CRISPRi screening data on human ESCs were retrieved from GEO (GSE127202^[^ ^31^ ^]^). Samples with fewer than ten cells and cells with multiple guide RNAs were removed. A *T‐*test was then performed between knockout and control samples, leaving only samples with significant downregulation (*P* < 0.05 and logFC < −1; Figure S2a, Supporting Information). After preprocessing, an expression matrix of 4325 cells containing 40 targeted TFs across 106 samples was retained for further analysis. The overall performance of metaTF was compared with SCENIC and DoRothEA on CRISPRi data with 40 target TFs. MetaTF identified all the target TFs and performed best in nearly all TFs (Figure 2a). To elucidate the pivotal contributions of two key steps in inferring TF activity using metaTF, the activities of 35 TFs were recalculated by separately removing the steps involving PUIC for GRN calculation and the prior network. The findings revealed that, compared with PUIC alone, the inclusion of the prior network resulted in 32 TFs achieving higher AUROC values, indicating that the inclusion of the prior network enables metaTF to achieve better performance. Simultaneously, when excluding the PUIC calculation of the GRN step and retaining only the prior network, metaTF achieved results comparable to SCENIC (Figure S5a,b, Supporting Information). In Figure 2a, *SOX17* has AUROC values much higher than other factors. The estimation of *SOX17* activity using metaTF and SCENIC indicated that the knockout of *SOX17* led to a significant decrease in the expression of its target genes, including *GATA4*, *GATA6*, *FOXA2*, and *SOX7* (Figure S5c, Supporting Information). The downregulation of these TFs subsequently affects the expression of other downstream genes, leading to substantial changes at the transcriptional level. These changes are particularly beneficial for accurately estimating *SOX17* activity, thereby enhancing the precision of *SOX17* activity estimation.

Consistent with previous analysis of human single‐cell CRISPR screening data, the prior knowledge‐based network was replaced with a mouse TF‐target network to assess the activity of these four TFs in perturbed cells and control cells from mouse brain cortical development^[^ ^122^ ^]^ using three methods: metaTF, SCENIC, and DoRothEA. The AUROC values were calculated for each method (Figure S7, Supporting Information). The results showed that metaTF achieved the highest AUROC values for *Foxg1*, *Nr2f1*, and *Tcf4*, indicating that the TF regulons identified by metaTF in mouse can reflect TF activity with a relatively high degree of accuracy.

### TF Activity Inference in Bone Marrow Plasma Cells with MetaTF and T2T‐CHM13 Alignment

The T2T‐CHM13 reference genome, a significant improvement over GRCh38,^[^ ^123^ ^]^ was used to analyze scRNA‐seq data from healthy human bone marrow plasma cells.^[^ ^40^ ^]^ Sequencing reads were aligned to T2T‐CHM13 to generate a gene expression matrix, which was filtered based on original study criteria. TF activities were inferred using metaTF with a blood‐associated regulatory network as the prior. Highly variable genes and TFs (top 500 by coefficient of variation (CV)) were selected for Uniform Manifold Approximation and Projection (UMAP) clustering. Gene expression clustering confirmed IgA and IgG plasma cell populations, though isotype segregation was less distinct (Figure S10a, Supporting Information). TF activity analysis revealed three major clusters (Figure S10b, Supporting Information), with specific TFs showing elevated activity (Figure S10c, Supporting Information). These findings support the hypothesis that bone marrow plasma cells retain tissue‐of‐origin transcriptional programs, reflecting their diverse tissue origins.

### Preprocessing of Single‐Cell CRISPR Data with Double‐Knockout TFs In CD8^+^ Cytotoxic T‐Cell Differentiation

As described by Zhou et al.^21^, they re‐engineered a dual‐guide, direct‐capture lentiviral single guide RNA (sgRNA) vector to generate a modified Ametrine‐expressing retroviral vector that effectively transduced primary CD8^+^ T cells. The dataset was downloaded from single‐cell CRISPR screens pertaining to the differentiation of CD8^+^ cytotoxic T cells. Data from cells were retained with only one type of sgRNA, those with two types of sgRNAs, as well as data from control cells. sgRNA with fewer than ten cells were removed. A *T*‐test was then performed between knockout and control samples, leaving only samples with significant downregulation (*P* < 0.05 and logFC < −1). After preprocessing, an expression matrix of 53 774 cells containing 109 single‐targeted and 426 double‐targeted TFs was retained for further analysis. The overall performance of metaTF with DoRothEA on CRISPRi data was compared with 109 single‐targeted TFs. For double‐targeted TFs, the target TFs metaTF activity scores were compared with the control cells.

### Evaluating the Robustness of TF Activity Estimation Methods through Synthetic Dropout Simulation

Single‐cell CRISPRi screening data were used to evaluate the robustness of TF activity estimation methods through synthetic dropout simulation. The expression matrix was randomly zeroed out at various dropout ratios (5%, 10%, 20%, and 30% of total values) to create sparser matrices. For each simulated expression dataset, TF activities were inferred using metaTF, SCENIC, and DoRothEA. Specifically, metaTF integrated the prior‐based network with the GRN‐based network using the “integraNets” method with parameters “max_target_num = 2000” and “maxInter = 10.” For SCENIC, the “runSCENIC” method was used with default parameters. For DoRothEA, the “run_viper” method with built‐in data from the DoRothEA package was used as the default parameter. Three gene activity inference methods (VIPER, ssGSEA, and AUCell) were assessed using the same regulons identified by metaTF. For each method and dropout level, the AUC value of each TF was calculated.

The impact of low coverage on metaTF performance was further assessed using 10× Genomics scRNA‐seq data from melanoma cultures (GSE134432^[^ ^32^ ^]^). Synthetic dropout was simulated to evaluate metaTF's robustness under varying dropout levels. Single melanoma sample MM074 cells were treated with *SOX10* perturbation (knockdown group: MM074_SOX10_72 h) or nontargeting siRNA (control group: MM074_NTC; Figure S3a–c, Supporting Information). *SOX10* activity was predicted in knockdown cells after applying random zeroing at dropout ratios from 5% to 50% (Figure S3d, Supporting Information). MetaTF was used to infer *SOX10* activities, integrating prior‐based and GRN‐based networks via “integraNets” with parameters “max_target_num = 2000” and “maxInter = 10.” As expected, with increasing dropout rates, the number of detected genes progressively decreases, and the performance of metaTF also declines gradually (Figure S3e, Supporting Information). When the median number of detected genes per cell falls below 2500, there is a significant decline in the predictive performance of metaTF. Specifically, when the median number of detected genes drops below 2000, the accuracy of metaTF's predictions hovers around 50%. At this point, imputation methods (e.g., MAGIC,^[^ ^79^ ^]^ DeepImpute^[^ ^80^ ^]^) can be employed to enhance data quality.

### Evaluating the Performance of MetaTF Using In Vivo *Nkx2‐1* Knockout Data

Droplet‐based scRNA‐seq data consisting of lung cells from *Nkx2‐1* ^CKO/CKO^; *Aqp5* ^Cre^ *^/+^* mutant mice and littermate controls were downloaded from GEO (GSE129584^[^ ^33^ ^]^). Since the *Nkx2‐1* conditional knockout (cKO) primarily affected alveolar type 1 (AT1) cells, the analysis exclusively focused on epithelial cells, in which *Nkx2‐1* plays a key role. t‐SNE analysis on mutant and control cells was first performed using the R package scater,^[^ ^124^ ^]^ clustering them into 28 clusters (Figure S4a, Supporting Information). Clusters with over 50% of cells expressing *Cdh1*, an epithelial marker gene, were further retained (Figure S4b, Supporting Information). This resulted in 2085 control and 2484 *Nkx2‐1* ^CKO/CKO^; *Aqp5* ^Cre^ *^/+^* mutant epithelial cells. The t‐SNE results showed that *Nkx2‐1* was only expressed in WT cells, indicating a successful knockout of *Nkx2‐1* in AT1 cells (Figure S4c, Supporting Information). For the benchmarking process, TF activity matrices for these epithelial cells were calculated using standard metaTF, simplified SCENIC, and standard DoRothEA workflows. The ROC and AUC values for each method were calculated as previously described.

### Evaluating the Performance of MetaTF Using In Vivo *Cebpa* Knockout Data

Massively parallel single‐cell RNA sequencing (MARS‐seq) data consisting of myeloid progenitors from *Cebpa* conditional knockout (*Cebpa* ^flox/flox^ *Mx1‐Cre*) and matching control (*Cebpa* ^flox/flox^) mice were downloaded from GEO (GSE72857^[^ ^34^ ^]^). After quality control, a total of 1152 control and 2304 *Cebpa* mutant cells were retained for analysis (Figure S4d, Supporting Information). An analogous benchmarking process to the *Nkx2‐1* knockout data was employed, with the distinction being that the prior GRNs were based on “myeloid_leukocyte”‐specific data. The results showed that metaTF still performed better (AUROC of 0.68) than other tools (Figure 2b).

### Evaluating the Performance of MetaTF Using TF Overexpression Data

Single‐cell Smart‐seq2 data from induced hemogenic endothelial cells (iHECs) with exogenous *Runx1* and *Hoxa9* overexpressions were downloaded from GEO (GSE128738^[^ ^35^ ^]^). After quality control, a total of 26 embryonic HECs and 65 iHECs were retained for analysis. An analogous benchmarking process to the *Nkx2‐1* knockout data was employed, with the distinction being that the prior GRNs were based on blood‐associated data, and cells with TF overexpression were considered as the positive class when computing ROC and AUC values.

### Pathway Enrichment Analysis of Regulons

To identify the biological pathways associated with each regulon, pathway enrichment analysis was performed using the Jaccard test.^[^ ^125^ ^]^ This test evaluated the similarity and statistical significance between the regulon gene set and pathway gene lists based on their shared genes. Notably, the initial enriched pathway terms for each regulon contained redundancies. To address this issue, Jaccard similarity coefficients between all pairs of enriched terms were calculated and then clustered into nonredundant groups using unsupervised hierarchical clustering. The enriched term with the smallest *P*‐value in each group was selected to represent that group. By default, the Reactome pathway database^[^ ^126^ ^]^ (version 77) covering 16 species was used for enrichment analysis. Only terms containing 10–2000 genes were included. This was allowed for inferring key biological pathways linked to each regulon in a nonredundant manner.

### Analysis of Single‐Cell RNA Sequencing Data during HSC Development

The raw single‐cell Smart‐seq2 data from six sequential cell populations (of note, E12 and E14 FL HSCs were combined) during HSC development were sourced from the GEO datasets (GSE153653 and GSE67120) derived from the recently published study.^[^ ^22^, ^127^ ^]^ Initially, adaptor and low‐quality base trimming were performed through trimmomatic^[^ ^128^ ^]^ (version 0.36) using the parameters “LEADING:5 TRAILING:5 SLIDINGWINDOW:4:15 HEADCROP:20 CROP:101 MINLEN:101.” Subsequently, gene expression was quantified as counts using Salmon^[^ ^129^ ^]^ (version 1.3.0) with clean data derived from trimmomatic output, where reads were mapped to the mouse GENCODE^[^ ^130^ ^]^ transcriptome (mm10) and gene annotation (vM25, primarily assembled). Log normalization was performed using the “logNormCounts” function from the R package scater. TF activities were estimated with metaTF, using blood‐associated networks as prior network. The CV of all genes and TFs was then calculated using normalized counts or activity scores, and the top 500 highly variable genes/TFs were retained for t‐SNE analysis. To confirm the efficacy of TF activity profiles in more effectively classifying distinct cell types compared to gene expression profiles, a classic machine learning approach, random forest classification model, was employed on mouse embryonic HSCs cell populations. Separate models were fitted using TF activity profiles and gene expression profiles, and models were trained in Python package scikit‐learn with “RandomForestClassifier” method. Cell‐type specificity of TFs was determined at both the expression and activity levels using the “findAllCTSRegulons” method in the R package metaTF. TFs with adjusted *P* < 0.01 and cell‐type‐specific score (CSS) > 3 were considered significant cell‐type‐specific regulators. The “RadvizEnrichPlot” function in the R package metaTF was used to visualize cell‐type‐specific TFs, and the R package UpSetR^[^ ^30^ ^]^ (version 1.4.0) was used to generate Venn diagrams.

*Rela* loss impairs HSC function and promotes hematopoietic stem and progenitor cell (HSPC) cycling. To confirm that the target genes of *Rela* are indeed activated in HSCs, the enrichment of these targets was estimated using an independent dataset with *Rela* knockout (TF knockout/knockdown data from mouse bone marrow HSCs,^[^ ^38^ ^]^ GSE45755). Ideally, *Rela* knockout would lead to the downregulation of its target genes, so GSEA was performed using *Rela*’s target genes on logFCs calculated from *Rela* knockout and control samples. The R package fgsea was used to calculate the normalized enrichment score and adjusted *P*‐values. The enrichment plot showed that these target genes were significantly enriched in control samples (Figure 3g), indicating that the target genes of *Rela* identified with metaTF can be confirmed in other independent datasets. The same confirmation was also performed on another TF, *Sp1*, using microarray data with *Sp1* knockout^[^ ^39^ ^]^ (GSE52497).

### Analysis of Breast Cancer Epithelial Cell Single‐Cell RNA Sequencing Data

Breast cancer patient scRNA‐seq data from the GEO (GSE176087) were analyzed.^[^ ^131^ ^]^ Gene expression was log normalized, and cells with fewer than 500 expressed genes were filtered out, resulting in 13 062 genes across 25 605 epithelial cells. The Seurat v3.0.0 method was employed in R (v3.5.0) for data normalization, dimensionality reduction, and clustering. Specifically, to recluster epithelial lineages, cell signatures were utilized for breast epithelial subsets described by Lim et al.^[^ ^132^ ^]^ as input features. Subsequently, the FindIntegrationAnchors step was utilized to identify anchors, followed by alignment with 30 dimensions (IntegrateData step) within Seurat. The final clustering results were visualized using t‐SNE. TF activities were estimated using metaTF with stranded pipeline except that tissue‐associated networks were used as prior networks. The CV of each gene/TF was then calculated using normalized counts or activity scores, and only the top 500 highly variable TFs or genes were retained for dimensionality reduction analysis (t‐SNE). The cell‐type specificity of TFs at the expression and activity levels was estimated using the “findAllCTSRegulons” method in the metaTF package. TFs with adjusted *P* < 0.05, CSS > 0.3, and percent > 0.1 for each stage were considered significant. Three evaluation indicators (Between/within, Calinski–Harabasz index, 1/Davies–Bouldin index (1/DBI)) for clustering results were calculated to show that using TF activities can more closely cluster cell populations and separate different types of cells as much as possible. For gene set activity analysis in scRNA‐seq data, the AUCell method from the AUCell Bioconductor package was used to calculate AUCell scores.

The raw single‐cell count matrix data were downloaded from GEO (GSE180286). Then, all cells expressing <300 genes were removed, and >20% mitochondrial counts. Genes expressed in fewer than three cells were likewise removed. The Seurat default parameters were used unless stated otherwise. For the clustering of all cell types, 2000 variable genes were identified, and principal component analysis (PCA) was applied to the dataset to reduce dimensionality after regressing the percentage of mitochondrial genes. Data integration was performed using the Seurat functions FindIntegrationAnchors and IntegrateData. Clustering was conducted using the FindClusters function with 30 PCA components and a resolution parameter set to 1.2 and visualized using UMAP with the RunUMAP function. Cell types were annotated based on canonical cell‐type markers. Cells annotated as epithelial cells in each sample were extracted into a subset and separately re‐clustered, using the methods described above and the following parameters: *n*_features_ = 2000, *n*pcs = 20, and resolution = 0.5. Malignant epithelial cells were identified based on genome‐wide copy number profiles computed from the gene expression Unique Molecular Identifier (UMI) matrix using the Bayesian segmentation approach, CopyKAT^[^ ^133^ ^]^ (v1.0.5). Then, malignant epithelial cell types were annotated with scSubtype. Normal epithelial cell types were annotated based on canonical cell‐type markers. TF activities were estimated using metaTF with a stranded pipeline, except that tissue‐associated networks were used as prior networks.

### Validation on TCGA BRCA RNA‐Seq Dataset

The RNA‐seq data from breast cancer patients were downloaded from the TCGA database (https://portal.gdc.cancer.gov/), and subtyping was performed based on Berger et al.’s work^[^ ^52^ ^]^ (subtype PAM50). This dataset includes 575 LumA BRCA clinical subtype samples, 211 LumB BRCA clinical subtype samples, 198 Basal BRCA clinical subtype samples, 82 Her2 BRCA clinical subtype samples, 40 Normal‐like BRCA clinical subtype samples, 113 BRCA tumor adjacent normal tissue samples. Subsequently, the genesets of cancer cell‐specific activated regulons which were identified by metaTF in BRCA epithelial cell scRNAseq data were used as input for GSVA.^[^ ^53^ ^]^ GSVA scores were calculated, and differentially activated regulons were determined using the limma R package with the parameter “adjusted *P*‐value of <0.01” (one versus the rest).

### Analysis of Prostate Cancer Epithelial Cell Single‐Cell RNA Sequencing Data

Human prostate cancer single‐cell RNA‐seq data (GSE176031), excluding samples related to prostate epithelial organoids, were downloaded and epithelial cells were extracted while retaining the original cell type annotations. Subsequently, clustering analysis was performed on the five epithelial cell populations using metaTF and Seurat. In brief, gene expression was log‐normalized, and cells with fewer than 500 expressed genes were filtered out, resulting in 14 546 epithelial cells. TF activities were estimated using metaTF with a stranded pipeline, except that tissue‐associated networks were used as prior networks. The CV of each gene/TF was then calculated using normalized counts or activity scores, and only the top 500 highly variable TFs or genes were retained for dimensionality reduction analysis (UMAP). To validate that TF activity can more effectively distinguish different epithelial cell populations, all epithelial cell TF activity profiles, gene expression profiles, and cell type annotations were inputted into a random forest classification model. After model training (using the RandomForestClassifier method from the scikit – learn Python package and specifying ‘max_depth = 5′ as one of the parameters), precision and recall values were calculated for each cell population.

### Total RNA Extraction and Quantitative Real‐Time PCR

Total RNA was extracted from tissues or cells with Trizol reagent (Invitrogen, United States). RNA was reverse‐transcribed into Complementary DNA (cDNA) using reverse Transcriptase M‐MLV (Takara, Dalian, China). The relative expression of genes was detected with Hieff Quantitative Polymerase Chain Reaction (qPCR) SYBR Green Master Mix (#11201ES08 Yeasen Biotechnology, Shanghai, China).

### Cell Cultures

The human TNBC cell lines BT549 and Hs578T were cultured in Dulbecco's Modified Eagle Medium (DMEM) containing 10% fetal bovine serum (Procell Life Science & Technology, China.) at 37 °C in a 5% CO_2_ humidified atmosphere.

### Transfection of siRNAs

The siRNAs targeting 15 TFs and negative control were synthesized by Sangon Biotech (Shanghai, China). The sequences of these siRNAs are included in Table S16 (Supporting Information). The transfection was mediated by jetPRIME transfection reagent (Polyplus‐transfection, France) according to the manufacturer's protocol.

### Cell Migration and Invasion Assay

Transwell chambers (6.5 mm insert, 24‐well plate, 8.0 µm polycarbonate membrane, Corning Incorporated, Corning, NY, USA) with or without Matrigel were used to measure the invasion and migration ability of breast cancer cells in vitro. Briefly, BT549 and HS578T cells transfected with siRNAs were seeded in the upper chamber of transwell at the density of 5 × 10^4^ cells (for the migration assay) or 8 × 10^4^ cells (for the invasion assay) in serum‐free DMEM medium, and DMEM containing 10% fetal bovine serum was added to the lower chamber. After culturing for 24 h (for the migration assay) or 48 h (for the invasion assay), the upper chamber cells were wiped by using cotton swabs, and fixed with crystal violet. The number of cells across the filter was counted by microscopic imaging system.

### Cell Proliferation Assay

Breast cancer cells (BT549 and Hs578T) were plated in 96‐well plates at a density of 5000 cells per well. At 0, 24, 48, and 72 h, 20 µL of 3‐(4,5‐Dimethylthiazol‐2‐yl)‐2,5‐diphenyltetrazolium bromide (MTT) reagent (BS186, Biosharp) was added to each well and mixed thoroughly. Following incubation for 4 h at 37 °C, Dimethyl Sulfoxide (DMSO) was mixed with the cell–MTT suspension for 10 s using a plate shaker, the conversion of substrate to chromogenic product by live cells was detected using a microplate reader to analyze cell viability.

### Analysis of Breast Cancer Lymphoid Cell Single‐Cell RNA Sequencing Data

Two breast cancer patient scRNA‐seq datasets from the 10× Genomics (https://www.10xgenomics.com/resources/datasets) were analyzed. The data were integrated in R using Seurat v3.1.1 with the following steps: i) identified 2000 integration anchors using Seurat::FindIntegrationAnchors, ii) integrated the objects with Seurat::IntegrateData, iii) scaled the data and regressed out the nCount_RNA using all genes, and iv) calculated the top 30 PCs for dimensional reduction. TF activities were estimated with metaTF using tissue‐associated networks as prior network. The CV values for all gene/TFs were calculated using normalized counts or activity scores, and then the top 2000 highly variable genes or the top 500 highly variable TFs were retained for UMAP analysis. Differentially expressed genes between CD8^+^ T_EM_ C1 and C2 populations were identified using Seurat::FindMarkers function with the parameters “min.pct = 0.3, log.fold.change = 0.3.” To examine the consistency of the findings in other breast cancer dataset, an scRNA‐seq dataset of human breast cancer T cells (GSE110686) was reanalyzed, and the same processing steps were performed.

For the epinephrine RNA‐seq data, reads were aligned to the human reference genome (Gencode^[^ ^130^ ^]^ v43) with HISAT2^[^ ^134^ ^]^ v2.0.52. Gene counts were derived with featureCounts^[^ ^135^ ^]^ v2.0.3. Differential expression analysis was performed using edgeR^[^ ^136^ ^]^ with the parameters dispersion = 0.4 and *P* < 0.05. Figures were generated in the R package ggplot2 (https://github.com/tidyverse/ggplot2).

### The Extraction of Tumor‐Infiltrating Lymphocytes

Tumor tissue samples were obtained by surgical excision and dissociated into single‐cell suspensions for 3 h using 1 mg mL^−1^ collagenase IV. Density gradient centrifugation was performed using lymphocyte separation medium to separate lymphocytes from other cell types. Isolated lymphocytes were washed with Phosphate Buffered Saline (PBS) to remove residual cell debris and surface staining was performed with Fluorescein Isothiocyanate (FITC) anti‐human CD8 antibody (1:20 dilution, OKT8, Biolegend) at 4 °C in the dark for 30 min. After staining, cells were resuspended with PBS and filtrated through a mesh filter (40 µm), and then CD8^+^ T lymphocytes were specifically isolated by BD FACSAria II cell sorter. Tumor‐infiltrating T cells were cultured in CST AIM V Medium (Gibco, Thermo Fisher Scientific) supplemented with 10% FBS (Gibco, Thermo Fisher Scientific) and Human Recombinant IL‐2 (500 units mL^−1^, #78220, STEMCELL Technologies) for 15 days. The cells were treated with epinephrine (0.1 ng mL^−1^, Sigma‐Aldrich) for 24 h followed by RNA sequencing.^[^ ^71^ ^]^

### Immunofluorescence Staining and Confocal Laser Scanning Microscopy

Human breast cancer and tumor‐infiltrating T cells were fixed with 4% cool paraformaldehyde for 10 min and blocked with 1% Bovine Serum Albumin (BSA) for 30 min. After washing in PBS, breast cancer tissues were stained with NF‐H Polyclonal antibody (1:100 dilution, 21471‐1‐AP, Proteintech) at 4 °C overnight in the dark, followed by FITC goat aAtirabbit IgG (H+L) (1:100 dilution, ZF‐0311, ZSGB‐BIO) and tumor‐infiltrating T cells were stained with anti‐ADRB2 antibody (1:100 dilution, AF6117, Affinity) at 4 °C overnight in the dark, followed by Rhodamine (TRITC) goat antiRabbit IgG (H+L) (1:100 dilution, AS040, ABclonal) at room temperature in the dark for 1 h. After three washes in PBS, the samples were stained with anti‐CD8 Mouse mAb (FITC Conjugate) (1:400 dilution, 55397S, Cell Signaling Technology) at room temperature in the dark for 3 h, and then nuclei were stained with 4',6‐Diamidino‐2‐Phenylindole (DAPI) (D3571, Invitrogen) for 5 min in the dark. Finally, the samples were sealed with sealing agent.

The expressions of ADRB2 and CD8 in breast cancer tissues were detected by polychromatic immunofluorescence staining. Polychromatic immunofluorescence staining was performed by using the three‐color multiple fluorescent immunohistochemical staining kit (HYDS0023, Guduo Technology, China) based on the tyramide signal amplification (TSA) technique following the manufacturer's manual. Briefly, deparaffinize and rehydrate breast cancer tissue sections, and then quench endogenous enzyme activity by incubating 30 min in 0.3% hydrogen peroxide and block nonspecific binding sites with goat serum for 10 min. The sections were incubated with primary antibodies against CD8 (AF5126, Affinity) at 4 °C overnight and incubated with secondary antibody labeled with Horseradish Peroxidase (HRP) provided with the kit for 50 min, followed by GD‐520 fluorescent dye diluted by the signal amplification reagent provided with the kit at room temperature in the dark for 15 min. The steps above were repeated, including the following: antigen retrieval and endogenous peroxidase blocking, the sections were incubated with primary antibodies against ADRB2 (AF6117, Affinity) at 4 °C overnight and incubated with secondary antibody labeled with HRP provided with the kit for 50 min, followed by GD‐570 fluorescent dye diluted by the signal amplification reagent provided with the kit at room temperature in the dark for 15 min. Finally, sections were counterstained with DAPI, sealed with glycerol and imaged using a ZEISS LSM 900 confocal microscope.

### RNA Extraction, Library Construction, and Sequencing

Total RNA was extracted using Trizol reagent (15596018, Thermo Fisher) following the manufacturer's procedure and the quantity and purity of RNA were detected by Bioanalyzer 2100 and RNA 6000 Nano LabChip Kit (5067‐1511, Agilent). After mRNA was purified by using Dynabeads Oligo (dT) (Thermo Fisher) and was fragmented into short fragments, and then the RNA fragments were reverse‐transcribed to create the cDNA by SuperScript II Reverse Transcriptase (18064014, Invitrogen), the products were amplified by PCR. Finally the high‐throughput sequencing (PE150) was performed on the Illumina Novaseq 6000 sequencer (LC‐Bio Technology Co., Ltd., Hangzhou, China).

### Cell‐Type Specificity Estimation

To evaluate the cell‐type specificity of TFs (or genes), an entropy‐based measure was used that quantifies the similarity between a TF activity and another predefined pattern, which represents the extreme state in which the TF is only activated in an indicated cell type. The specificity metric mostly relied on the Jensen–Shannon divergence^[^ ^137^ ^]^ (JSD), which was calculated as follows

(14) $$\mathrm{JSD}\left(g,g^{\prime }\right)=H\left(\frac{g+g^{\prime }}{2}\right)-\frac{H\left(g\right)+H\left(g^{\prime }\right)}{2}$$

where *g* and *g*′ represent two discrete probability vectors (such as the TF activity score) with equal length *n*, and *H* represent the entropy of discrete probability vectors as

(15) $$\begin{array}{c} H\left(p\right)=-\sum_{i=1}^{n}p_{i}\log\left(p_{i}\right),p=\left(p_{1},p_{2},\text{…},p_{n}\right),0\leq p_{i}\;\leq 1,\mathrm{and}\sum_{i=1}^{n}p_{i}=1 \end{array}$$

Then, the distance between two TF activity patterns, *r* ^1^ and *r* ^2^ was defined as

(16) $$\mathrm{JS}D_{\mathrm{dist}}\left(r^{1},r^{2}\right)=\sqrt{\mathrm{JSD}\left(r^{1},r^{2}\right)}$$

The CSS of a TF, $r=(r^{1},r^{2},\text{…},r^{m})$, in an indicated cell type was calculated as

(17) $$\mathrm{CSS}\left(r\right)=1-\mathrm{JS}D_{\mathrm{dist}}\left(r,r^{c}\right)$$

where *r* ^c^ represents a predefined pattern of the extreme case in which the TF is only activated in this cell type and was defined as

(18) $$r^{c}=\left(r_{1}^{c},r_{2}^{c},\text{…},r_{n}^{c}\right),s.t.r_{i}^{e}=\left\{\begin{array}{c} 1,i\in \;c\\ 0,i\notin \;c \end{array}\right.$$

In the context of TF activity data, the *i* represent each cell and the *n* represent the total number of cells. Meanwhile, for TF activity scores with negative values, and these were normalized into ranges from 0 to 1 as $\frac{x\;-\;x_{\min}}{x_{\max}\;-\;x_{\min}}$.

Next, to evaluate the significance of a TF that is specifically activated in an indicated cell type, the *P*‐value of TF was estimated using a bootstrap strategy. Specifically, for a TF in an indicated cell type, the cell labels were shuffled and the simulated CSS were calculated 10 000 times. Next, the *P*‐value was calculated as the percentage of the simulated CSSs that were greater than the actual CSS within all 10 000 simulated CSSs, and the Benjamini & Hochberg method was used to adjust the *P*‐values. Furthermore, the cell‐type‐specific methods were also suitable for gene expression.

### Statistical Analysis

To compare the means of three or more independent groups (Figure S4i,b, Supporting Information), a one‐way Analysis of Variance (ANOVA) was employed using the aov function from the stats package in R. If the overall ANOVA was significant (*P < 0.05), post‐hoc comparisons were performed using Tukey's Honestly Significant Difference (HSD) test to identify specific group differences. Thresholds for statistical significance are denoted by *P < 0.05, **P < 0.01, and ***P < 0.001.

## Conflict of Interest

The authors declare no conflict of interest.

## Author Contributions

Y.H., Y.Z., G.T., M.S., and P.T. contributed equally to this work. D.W., X.b.L., and Z.L. conceived the study and designed and supervised the study. D.W., X.b.L., Y.Z., and M.S. performed the experiments. Y.H., G.T., and P.T. performed bioinformatic analyses. D.W., X.b.L., Y.H., and Z.L. wrote the manuscript with input from all of the authors. Y.Y., X.Z., M.L., X.n.L., L.W., J.C., H.Z., Y.H., and Y.H. revised the manuscript.

## Supporting information



Supporting Information

Supplemental Table 1

Supplemental Table 2

Supplemental Table 3

Supplemental Table 4

Supplemental Table 5

Supplemental Table 6

Supplemental Table 7

Supplemental Table 8

Supplemental Table 9

Supplemental Table 10

Supplemental Table 11

Supplemental Table 12

Supplemental Table 13

Supplemental Table 14

Supplemental Table 15

Supplemental Table 16

Supplemental Table 17

Supplemental Table 18

Supplemental Table 19

Supplemental Table 20

## Data Availability

The data that support the findings of this study are openly available in Genome Sequence Archive in National Genomics Data Center, China National Center for Bioinformation / Beijing Institute of Genomics, Chinese Academy of Sciences at https://ngdc.cncb.ac.cn/gsa‐human, reference number 5590.
