# Supplementary material for: Accurate Transcription Factor Activity Inference to Decipher Cell Identity from Single‐Cell Transcriptomic Data with MetaTF
Source: Adv Sci (Weinh). 2025 May 21;12(23):e10745. doi: 10.1002/advs.202410745 (PMC12199337; doi:10.1002/advs.202410745)
Supplement: Supplementary file 1 — Supporting Information [file ADVS-12-e10745-s004.docx]

Supporting Information

**Accurate Transcription Factor Activity Inference to Decipher Cell Identity from Single Cell Transcriptomic Data with metaTF**

Yongfei Hu, Yuanyuan Zhu, Guangjue Tang, Ming Shan, Puwen Tan, Ying Yi, Xiyuan Zhang, Man Liu, Xinyu Li, Le Wu, Jia Chen, Hailong Zheng, Yan Huang, Zhuan Li ^*^, Xiaobo Li ^*^, Dong Wang ^*^

Yongfei Hu

Department of Bioinformatics, School of Basic Medical Sciences, Southern Medical University, Guangzhou 510515, China; Dermatology Hospital, Southern Medical University, Guangzhou 510091, China.

Yuanyuan Zhu, Xiyuan Zhang, Man Liu, Xiaobo Li

Department of Pathology, Harbin Medical University, Harbin 150000, China.

**Corresponding**: Xiaobo Li (Email: lixiaobo@ems.hrbmu.edu.cn)

Guangjue Tang, Puwen Tan, Xinyu Li, Le Wu, Jia Chen, Hailong Zheng

Department of Bioinformatics, School of Basic Medical Sciences, Southern Medical University, Guangzhou 510515, China.

Ming Shan

Department of Breast Surgery, Harbin Medical University Cancer Hospital, Harbin, China.

Ying Yi

Dermatology Hospital, Southern Medical University, Guangzhou 510091, China.

Yan Huang

Cancer Research Institute, School of Basic Medical Sciences, Southern Medical University, Guangzhou 510515, China.

Zhuan Li

Key Laboratory of Functional Proteomics of Guangdong Province, Department of Developmental Biology, School of Basic Medical Sciences, Southern Medical University, Guangzhou 510060, China.

**Corresponding**: Zhuan Li (Email: zhuanli2018@smu.edu.cn)

Dong Wang

Department of Bioinformatics, School of Basic Medical Sciences, Southern Medical University, Guangzhou 510515, China; Dermatology Hospital, Southern Medical University, Guangzhou 510091, China; Department of Bioinformatics, Fujian Key Laboratory of Medical Bioinformatics, School of Medical Technology and Engineering, Fujian Medical University, Fuzhou 350122, China.

**Corresponding**: Dong Wang (Email: wangdong79@smu.edu.cn)

**Extended Data Figures**

**
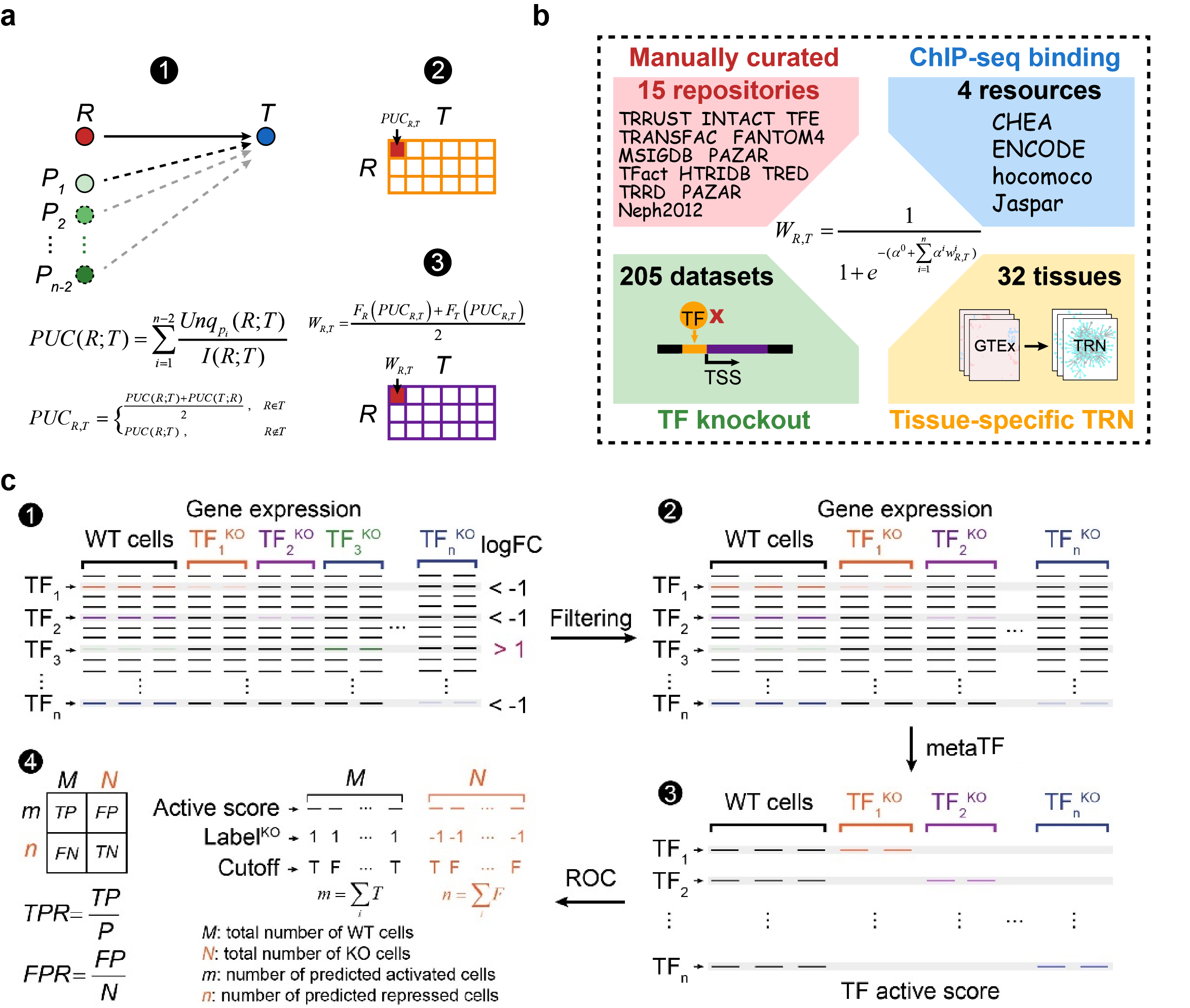
**

**Figure S1. Key steps in the metaTF framework. a** Schematic diagram showing the key steps of the PUIC algorithm. **b** Schematic illustration of four different resources used for prior-network construction. TSS, transcription start site. GTEx, genotype-tissue expression. TRN, transcriptional regulatory network. **c** Schematic diagram representing the performance evaluation steps using CRISPR screening data. Differential expression tests were first performed between WT and TF-targeted samples to assess the success of TF disruption. Then, we retained only samples with significant downregulation (logFC < -1) for the evaluation process. Next, we only estimated the activity of targeted-TFs in our single-cell RNA-seq data and compared their activity level between wild-type cells and TF-targeted samples. Ideally, target genes are not activated if their TF regulators are disrupted, so the activity of TFs in TF-targeted cells is theoretically lower than that in wild-type cells. Therefore, we labeled wild-type cells as the positive group and the TF-targeted cells as the negative group, respectively. For each TF, the TPR was estimated as the proportion of predicted positive cells in wild-type cells, while the FPR was estimated as the proportion of predicted negative cells in TF-targeted cells. Finally, we calculated hundreds of TPRs and FPRs by setting different activity score cutoffs. WT, wild-type; KO, knockout; TF, transcription factor; logFC, log-transformed fold change; ROC, receiver operating characteristic curve; P, positive; N, negative; TP, true positive; TN, true negative; FP, false positive; FN, false negative; TPR, true positive rate; FPR, false positive rate.


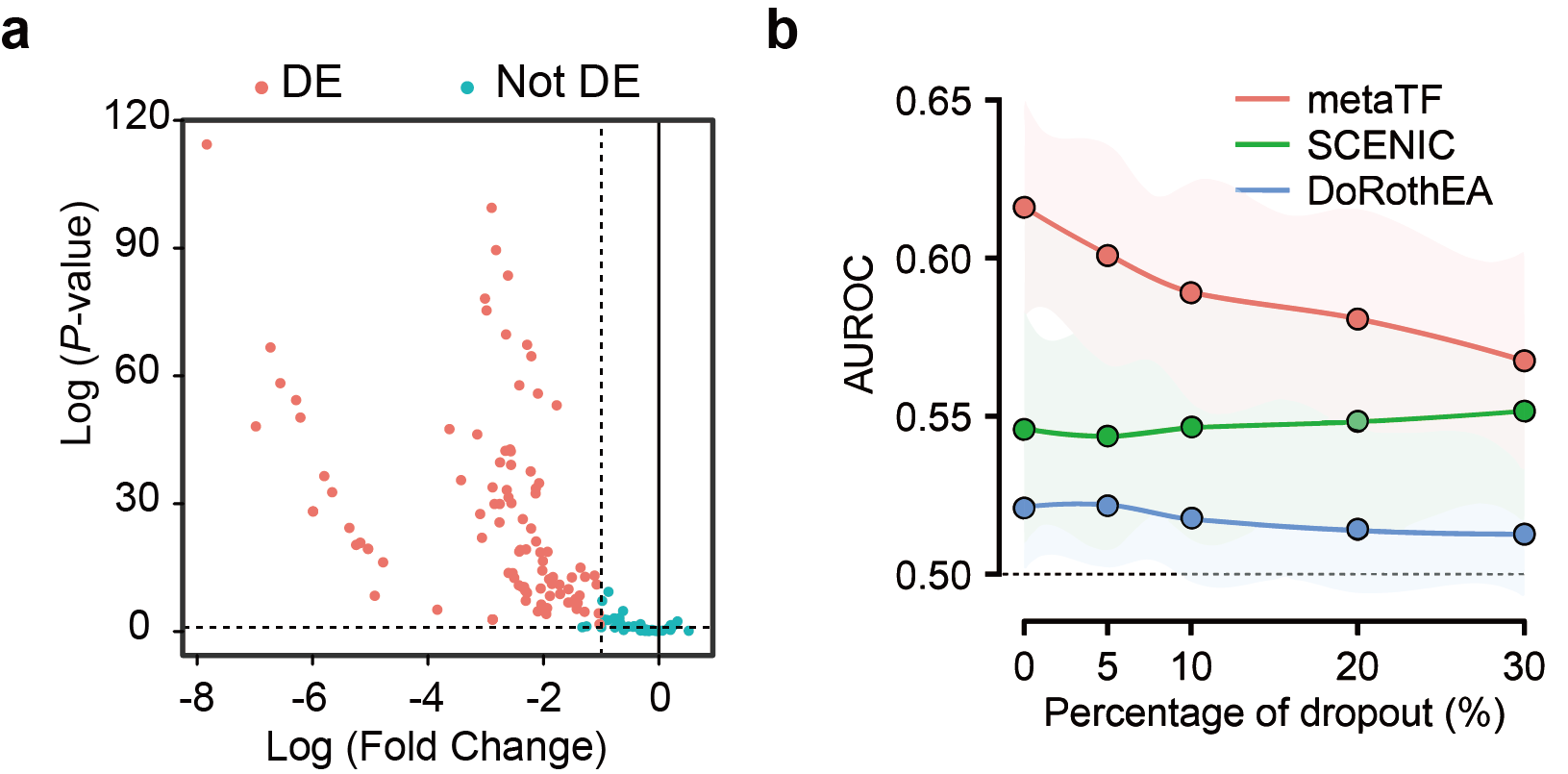


**Figure S2. Pre-processing of single-cell RNA-seq data with TF perturbations. a** Volcano plot representing the differentially expressed samples under TF perturbation in a CRISPRi dataset. DE, differentially expressed; logFC, log-transformed fold change. **b** Line plot showing the average AUROC values obtained using metaTF, SCENIC, and DoRothEA under different dropout rates.


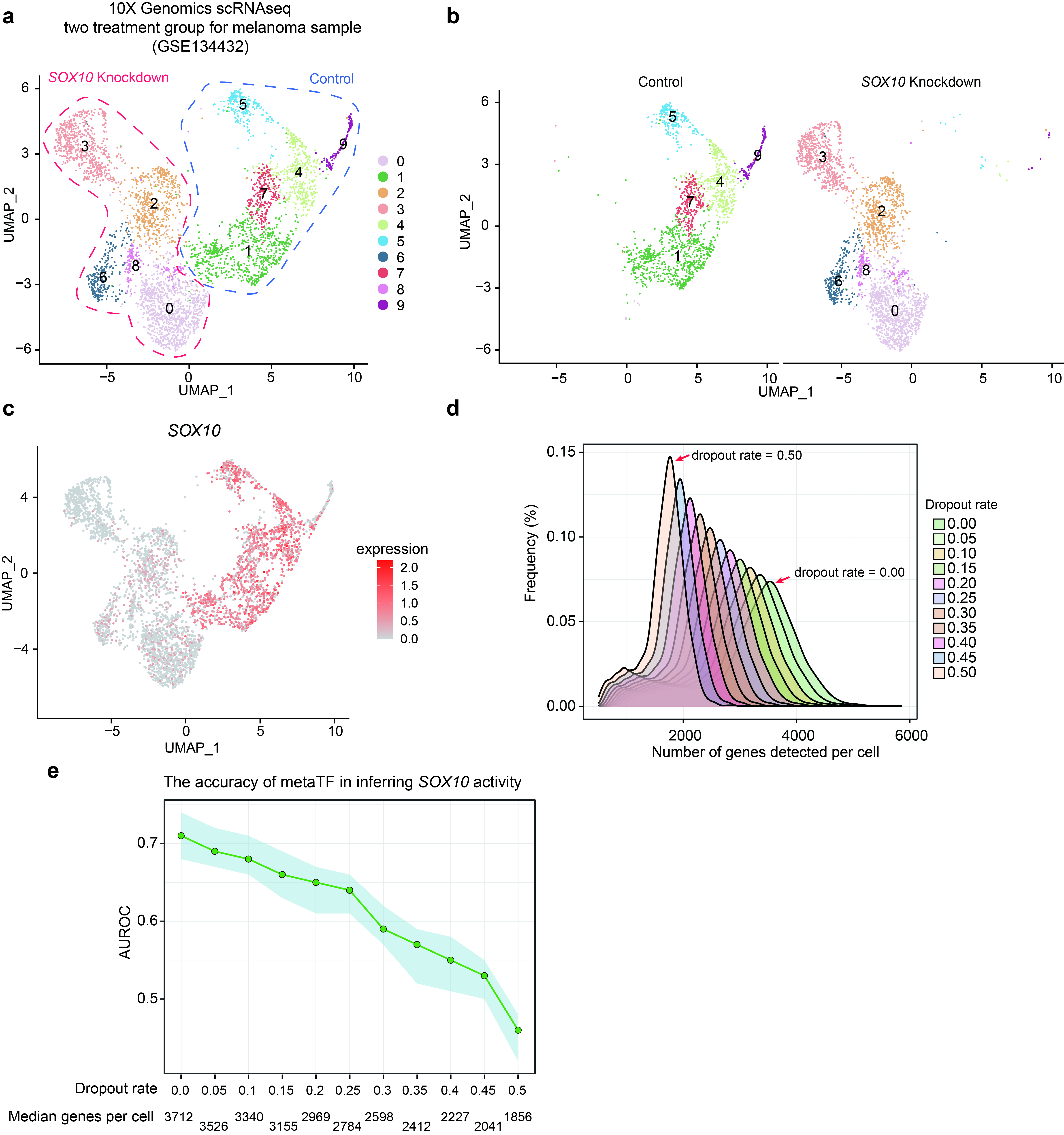


**Figure S3. The performance of metaTF was evaluated on low-coverage 10X Genomics scRNAseq data.** **a** The UMAP visualization illustrates melanoma cells collected from a single melanoma sample (GSE134432) that underwent two distinct treatments. One group of cells was subjected to SOX10 perturbation, resulting in SOX10 knockdown (treatment group name: MM074_SOX10_72h), while the other group was treated with non-targeting control short interfering RNA (treatment group name: MM074_NTC). In this visualization, the cells are distinctly colored according to their respective clusters. **b** UMAP plot of melanoma cells split by treatment. **c** UMAP visualization of melanoma cells colored by *SOX10* expression. **d** The frequency distribution plot illustrates the distribution of the number of genes detected per cell across all melanoma cells under varying dropout rates. As the dropout rates increase, the number of detected genes progressively decreases. **e** Line plot showing the average AUROC values obtained using metaTF under different dropout rates, with shaded color bands indicating the corresponding 95% confidence intervals. When the median number of detected genes per cell falls below 2500, there is a significant decline in the predictive performance of metaTF. Specifically, when the median number of detected genes drops below 2000, the accuracy of metaTF’s predictions is around 50%.


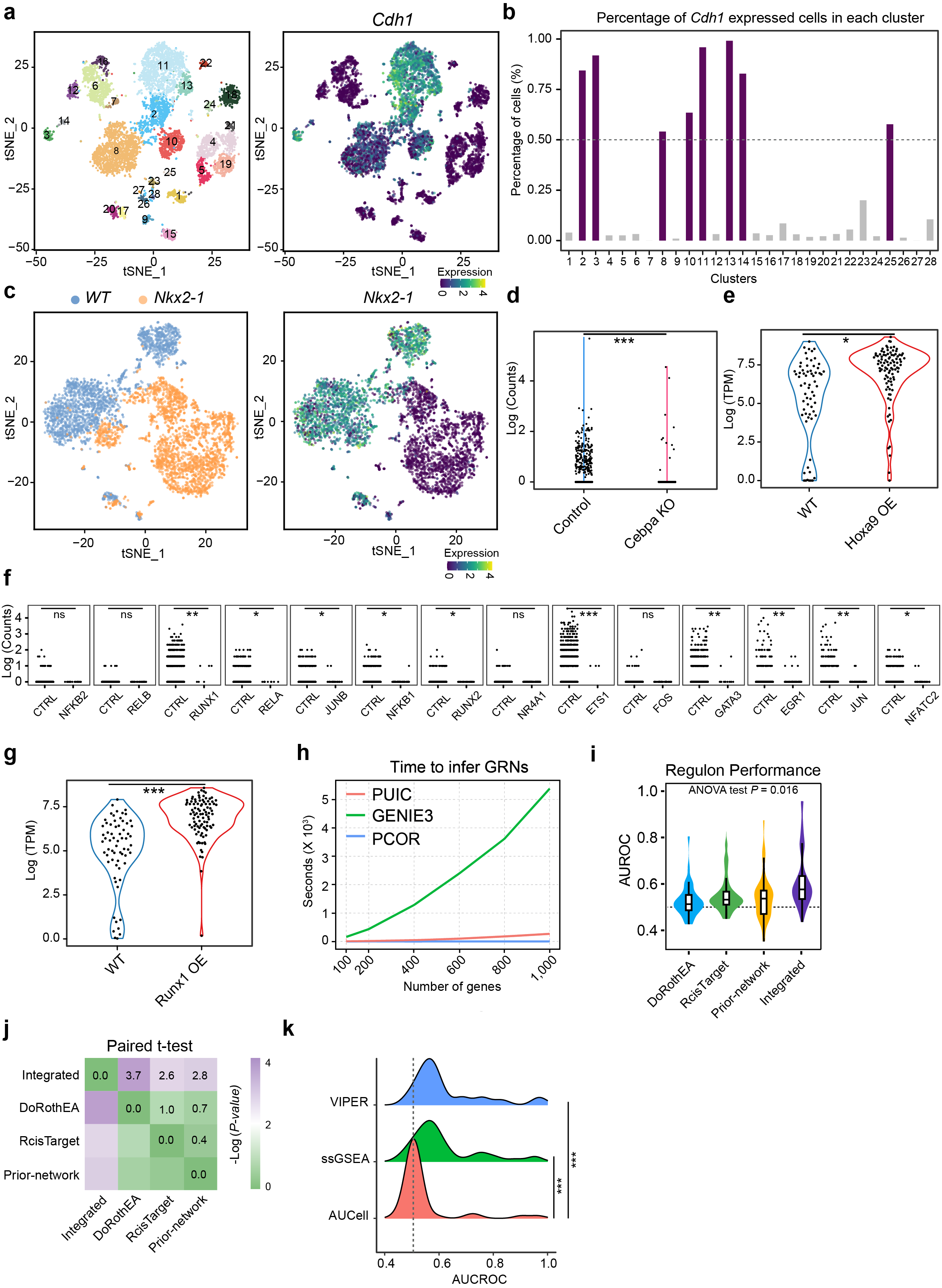


**Figure S4. Quality control of single-cell RNA-seq data with TF perturbations. a** t-SNE visualization of lung cells colored by clusters (left) and *Cdh1* expression (right) collected from *Little et al*. **b** Bar plot showing the percentage of *Cdh1* expression in each cluster. **c** t-SNE visualization of lung epithelial cells colored by sample (left) and *Nkx2-1* expression (right) collected from *Little et al*. KO, knockout. **d** Violin plot representing the expression of *Cebpa* in control and *Cebpa* cKO cells collected from *Paul et al*. A two-sided Wilcoxon rank-sum test was used to determine the difference (****P* < 0.001). **e** Violin plot representing the expression of *Hoxa9* in WT and *Hoxa9* overexpression cells collected from *Guo et al*. OE, overexpression. A two-sided Wilcoxon rank-sum test was used to determine the difference (**P* < 0.05). **f** Dot plot showing the expression of 14 perturbated TFs in a CROP-seq dataset. A two-sided Wilcoxon rank-sum test was used to determine the difference (**P* < 0.05). CTRL, control. ns, not significant. **g** Violin plot representing the expression of *Runx1* in WT and *Runx1* overexpression cells collected from *Guo et al*. A two-sided Wilcoxon rank-sum test was used to determine the difference (**P* < 0.05). OE, overexpression. **h** Line plot representing the running time of three GRN inference methods (including PUIC, GENIE3, and PCOR) with different numbers of genes. **i** Violin plot showing the area under the receiver operating characteristic (AUROC) of perturbated TFs calculated with five types of regulons. A one-way ANOVA was used to determine the difference. GRN, gene regulatory network. **j** Heatmap showing the significant differences in the AUROC values among the five types of regulons. A paired *T* test was used to estimate the significance. **k** Distribution of TF AUROC values calculated by three (VIPER, ssGSEA, and AUCell) different activity estimation methods. The Kolmogorov-Smirnov test was used to determine the differences (****P* < 0.001).


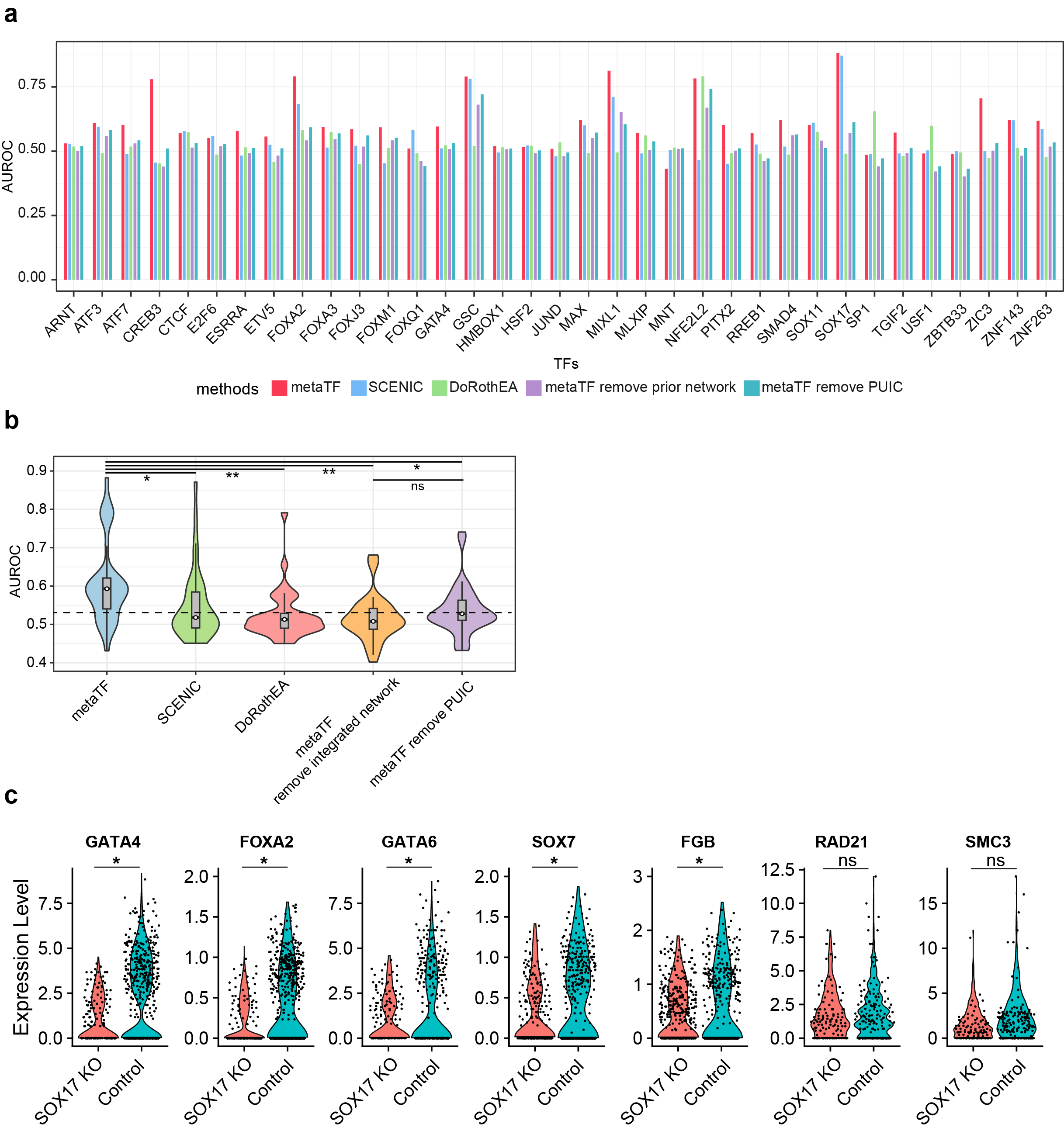


**Figure S5. The contribution of key steps in enhancing performance of metaTF. a** Bar plot illustrates the AUROC values of different methods for inferring the activity of 35 TFs, including recalculations after removing the steps involving PUIC for GRN calculation and omitting the prior network. **b** Violin plots depict the distribution of AUROC values obtained by different methods. A two-sided Wilcoxon rank-sum test was used to determine the difference (**P* < 0.05, ***P* < 0.01). ns, not significant. **c** The violin plot illustrates the expression levels of *SOX17* target genes in hESCs subjected to CRISPRi perturbation, comparing cells with and without *SOX17* KO. A two-sided Wilcoxon rank-sum test was used to determine the difference (**P* < 0.05). ns, not significant.


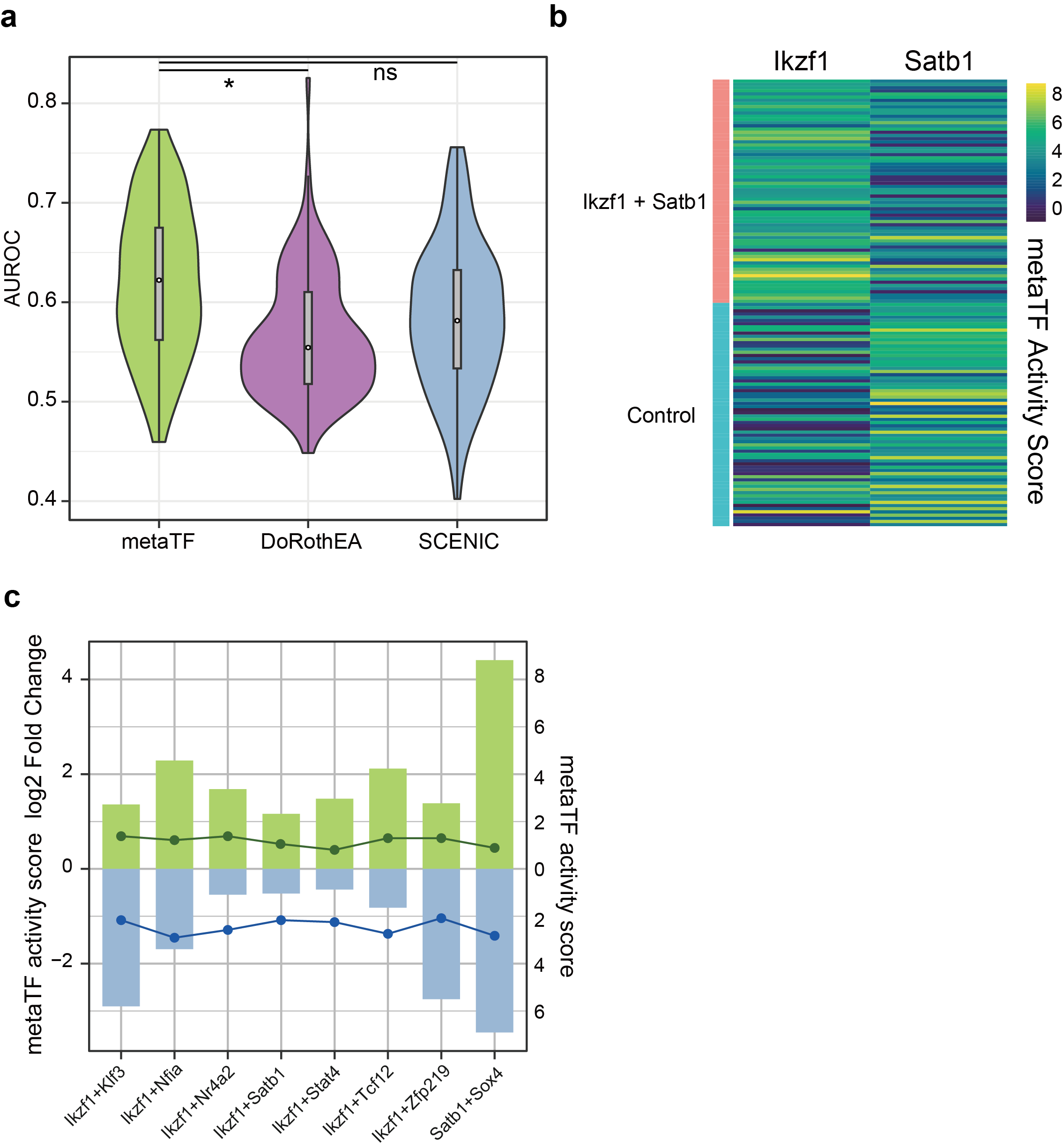


**Figure S6. metaTF’s performance for data of single-cell CRISPR screens from CD8^+^ cytotoxic T cell differentiation.** **a** Violin plot illustrates the comparison between the AUROC values obtained by metaTF, DoRothEA and SCENIC for 109 single targeted TFs. A two-sided Wilcoxon rank-sum test was used to determine the difference (**P* < 0.05). ns, not significant. **b** The heatmap illustrates the metaTF-predicted TF activity scores for Ikzf1 and Satb1 in both double-knockout cells and control cells. **c** The barplot displays the log2 fold change values of the metaTF-predicted TF activity scores in double-knockout cells compared to control cells. The green bar represents the log2 fold change value for the first TF, while the blue bar represents that for the second TF. In each pair, one TF showed elevated activity while the other decreased relative to the control. The horizontal dotted lines indicate the activity level of the TFs in control cells, with each dot corresponding to the average activity of a specific TF in the control group.


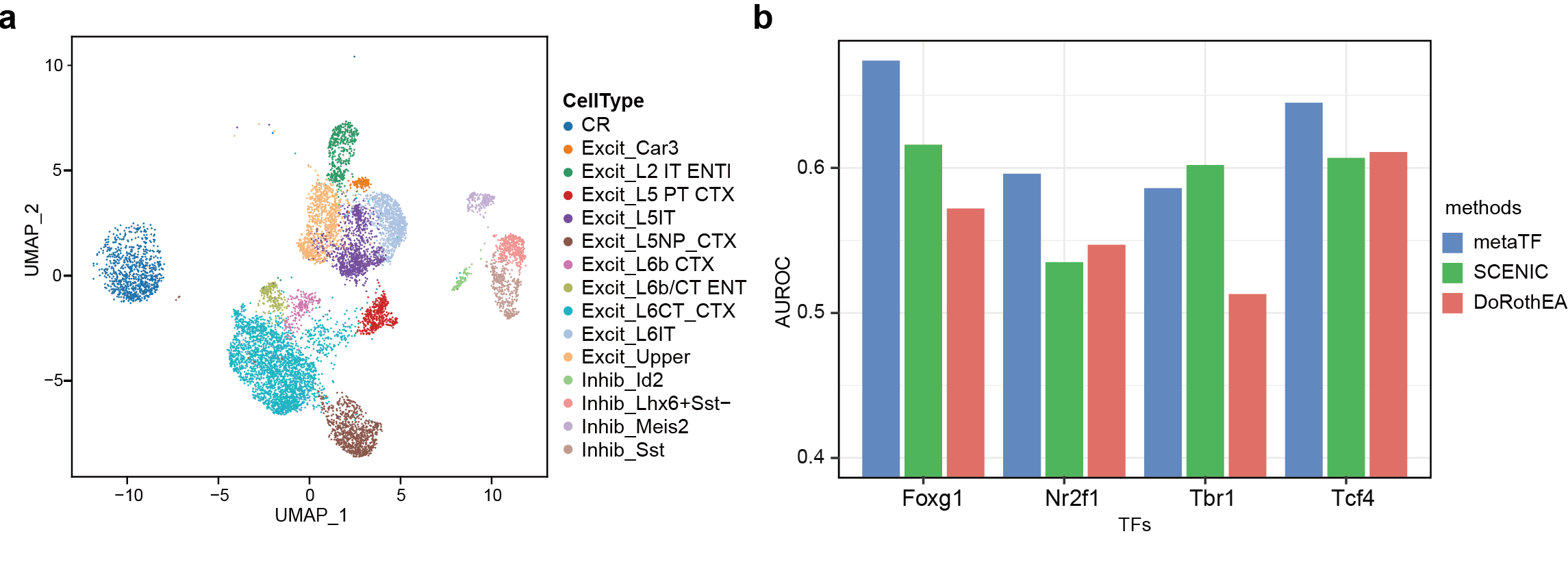


**Figure S7. Comparative analysis of three TF activity methods using single-cell CRISPR screening data from mouse brain cortical development. a** UMAP plot illustrates cell clustering results based on gene expression were obtained through re-analysis of single-cell CRISPR screening data from mouse brain cortical development. **b** Bar plot illustrates the comparison between the AUROC values obtained by metaTF, DoRothEA and SCENIC for 4 single targeted TFs.


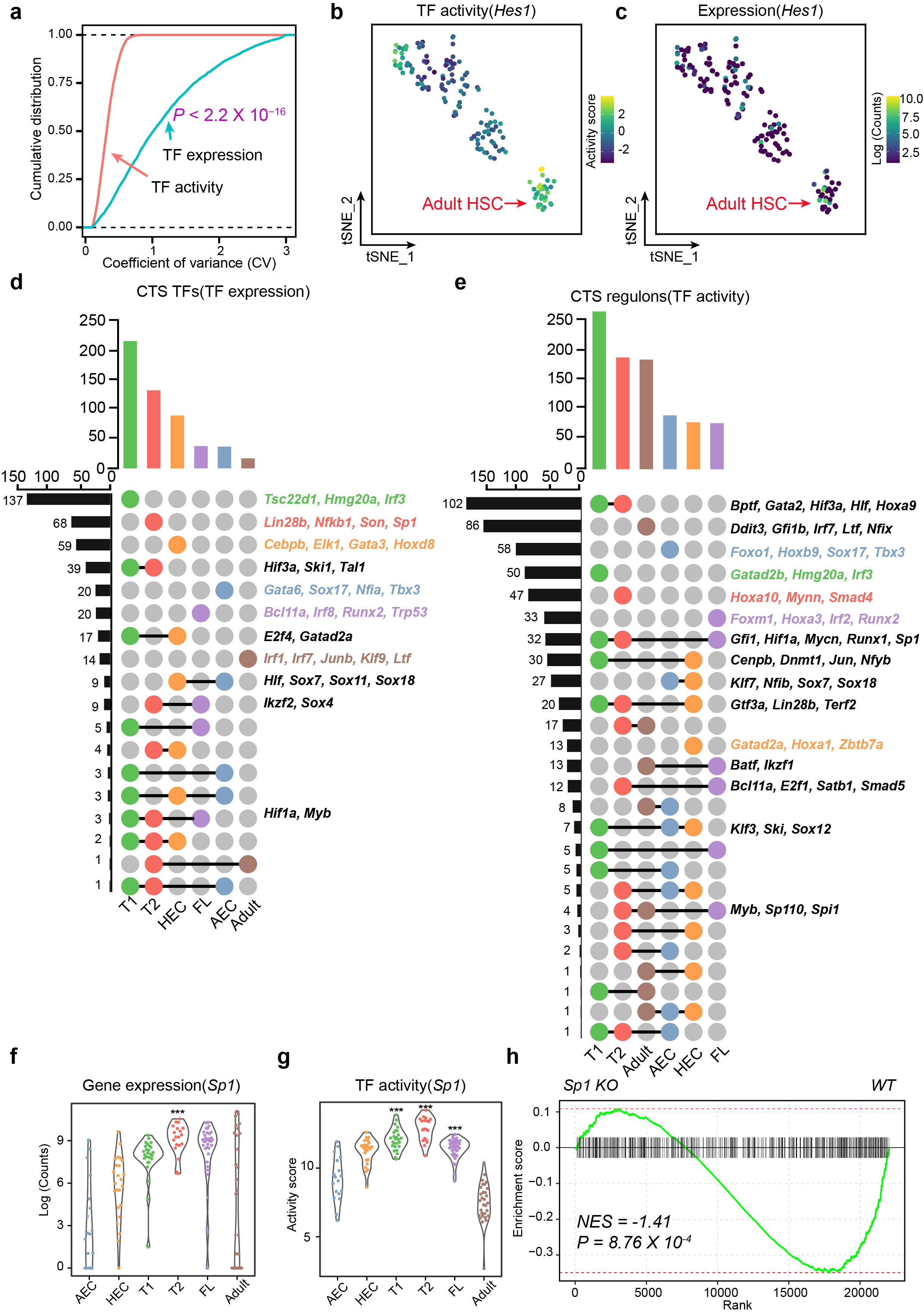


**Figure S8. Comparison between the TF expression and TF activity profiles. a** Cumulative distribution of cell type coefficient of variance (CV) between TF expression and TF activity profiles. The Kolmogorov-Smirnov test was used to determine the differences. **b** t-SNE plot showing the TF activity of *Hes1*. **c** t-SNE visualization of *Hes1* expression. **d** Venn diagram showing the distribution of CTS TFs of six cell types at the expression level. **e** Venn diagram showing the distribution of CTS TFs of six cell types at the activity level. **f** Violin plot representing the expression of *Sp1* in six sequential populations. A two-sided Wilcoxon rank-sum test was used to determine the difference (****P* < 0.001). **g** Violin plot representing the activity score of *Sp1* in six sequential populations. A two-sided Wilcoxon rank-sum test was used to determine the difference (****P* < 0.001). **h** GSEA showing the relative enrichment of *Sp1* target genes between WT and *Sp1* KO samples. NES, normalized enrichment score.


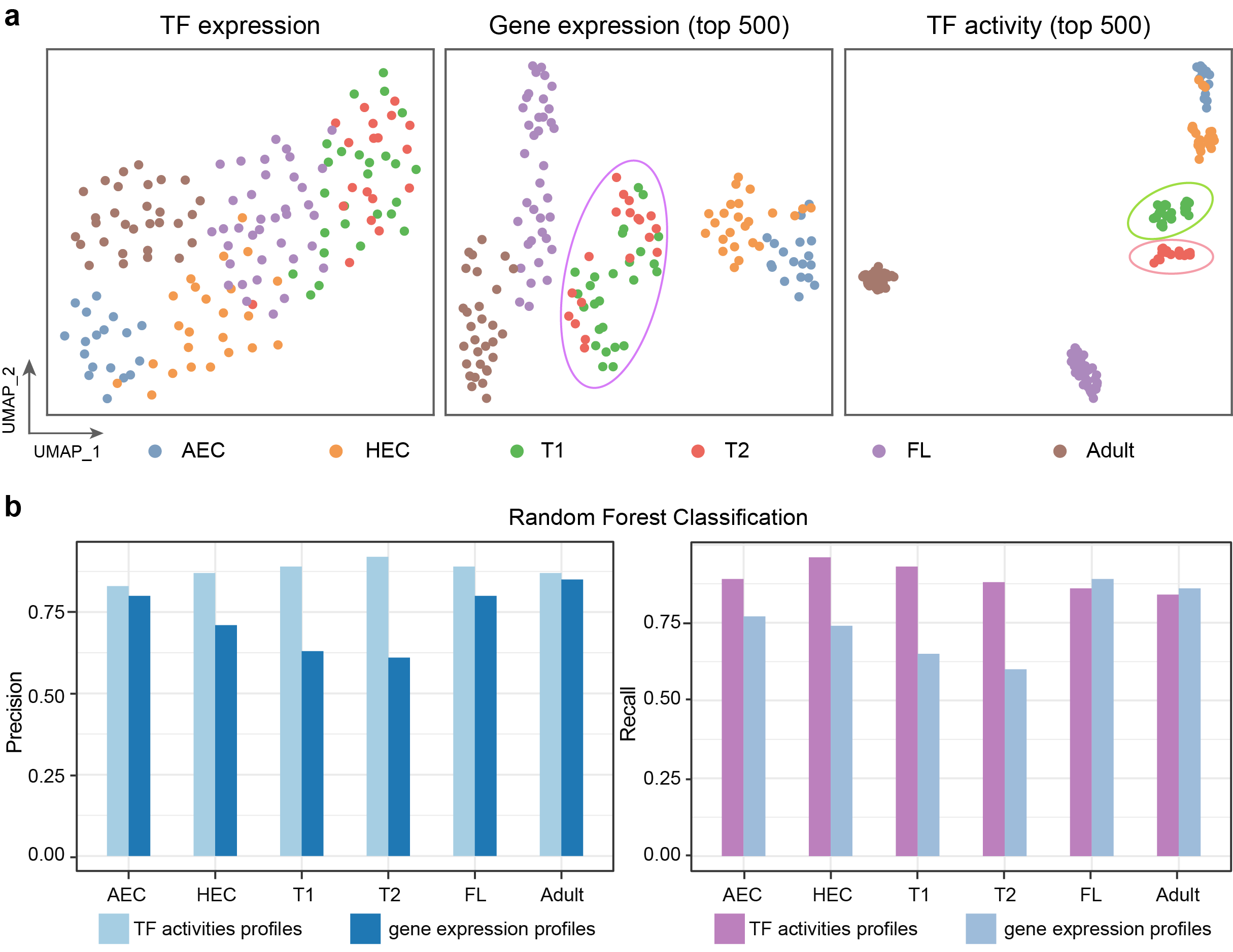


**Figure S9. Comparison of classification performance between TF activity profiles and gene expression profiles in distinguishing different cell types.** **a** UMAP plots visualizing the cluster assignments of mouse embryonic HSCs cell populations based on TF activity profile (right), TF expression profile (middle), highly variable genes expression profile (left). **b** In mouse embryonic HSCs cell populations, classic machine learning random forest classification models were employed to fit TF activity profiles and gene expression profiles, respectively. The right panel displays the precision of both models across different cell populations, while the left panel depicts the recall of both models across various cell populations.


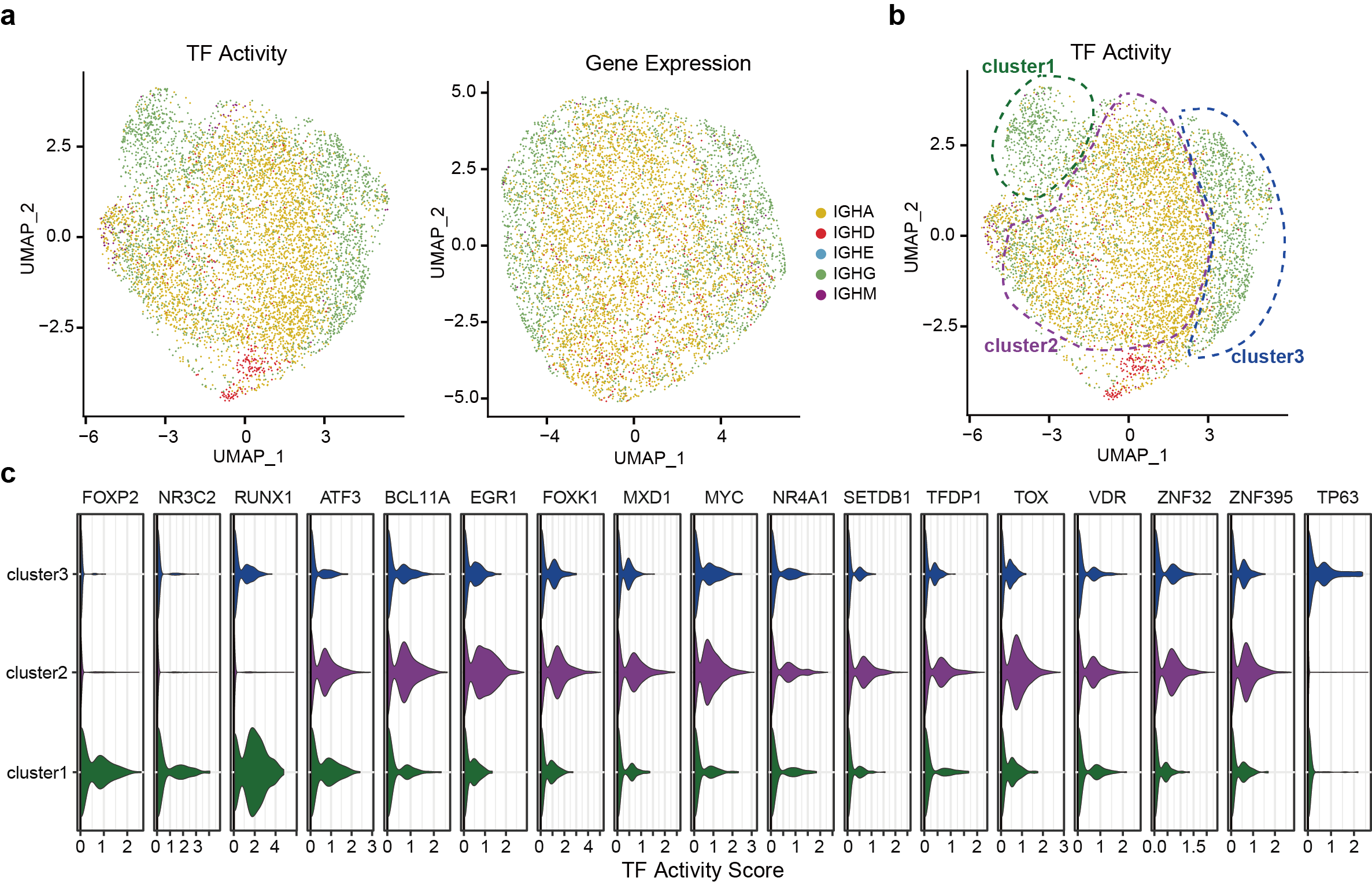


**Figure S10. The performance of metaTF in analyzing scRNA-seq data of human bone marrow plasma cells with human T2T-CHM13 genome alignment. a** Cell clustering based on gene expression or TF activity. **b** Formation of three clusters by IgA and IgG cells. **c** Identification of highly activated TFs in each cluster.

**
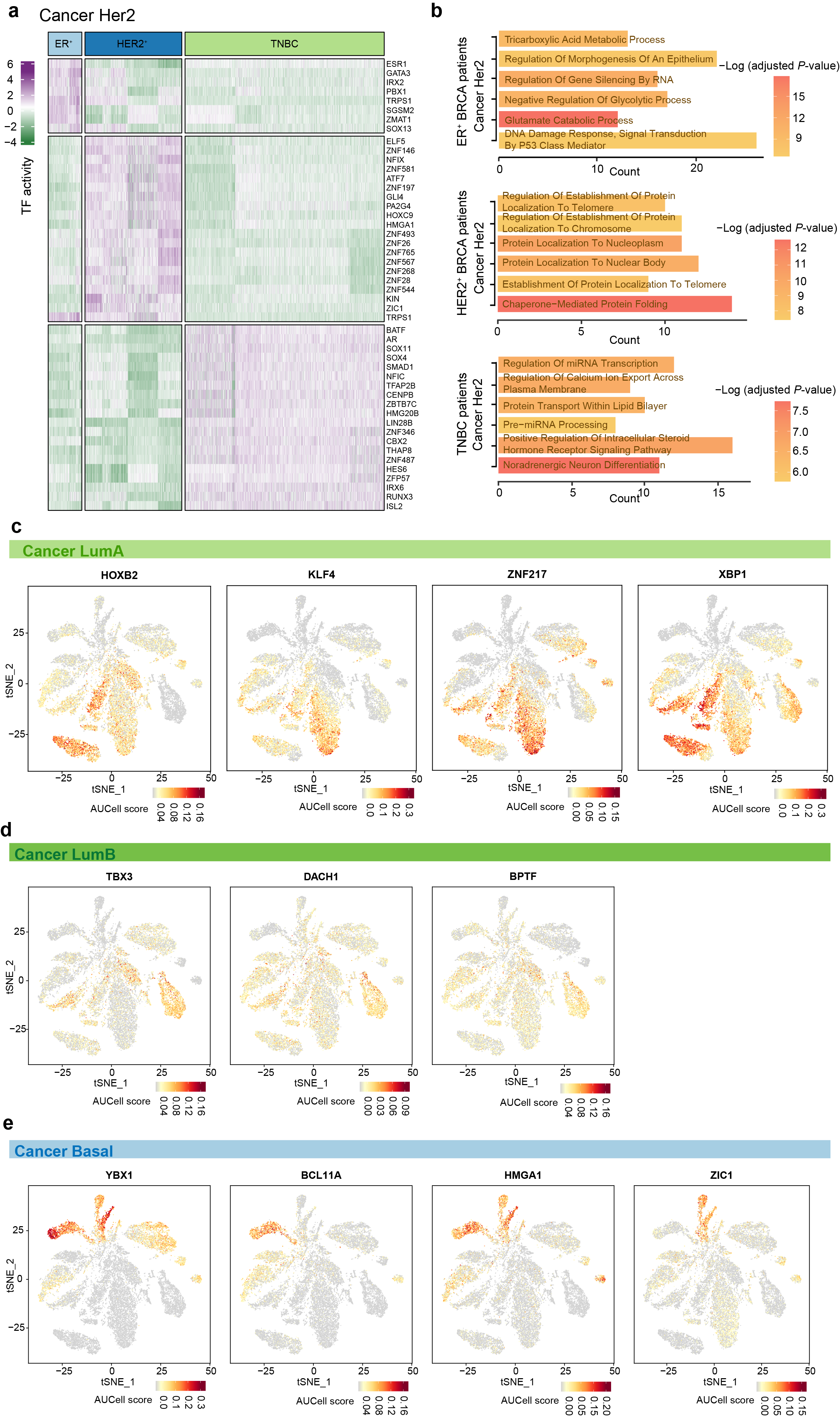
**

**Figure S11. metaTF reveals human breast cancer epithelial cell heterogeneity. a** Heatmap plot showing the activity score of specific activated TFs in Cancer Her2 epithelial cells across three BRCA clinical subtypes. **b** Bar plot showing the GO biological process enrichment results for specific activated TFs and their expressed targets in Cancer Her2 epithelial cells across three BRCA clinical subtypes. **c** Activity-based tSNEs (human breast cancer epithelial cell dataset by *Wu  et al.*) colored according to the AUCell scores of each cell for the given TFs and their expressed targets. As shown in Figure 4c, these TFs showed cancer epithelial cell type-specific activation characteristics in cancer epithelial cells Cancer LumA(**c**), Cancer LumB(**d**), and Cancer Basal(**e**).


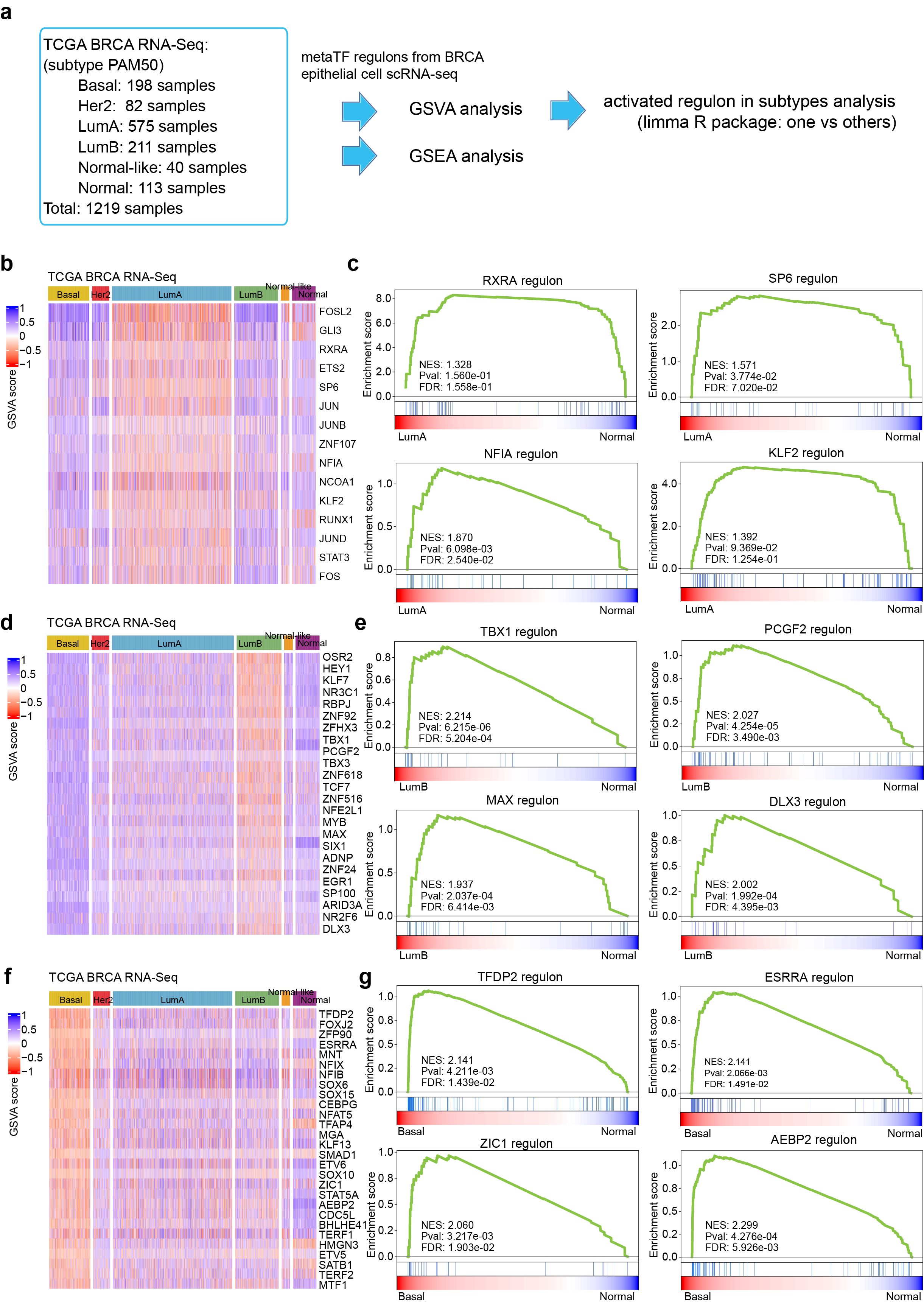


**Figure S12. Validation on TCGA BRCA RNA-Seq dataset.** **a** Schematic workflow for the validation on TCGA breast cancer RNA-Seq dataset. Briefly, the RNA-Seq data from breast cancer patients were downloaded, and subtyping was performed based on *Ashton*'s work. Subsequently, cancer cell-specific activated regulons which were identified by metaTF in BRCA epithelial cells were used as input for the GSVA. GSVA scores were calculated, and differential regulons were determined using the limma R package. **b** Heatmap plot showing 15 regulons differentially activated or enriched in the LumA subtype from GSVA analysis. **c** GSEA results of four representative regulons (*RXRA*, *SP6*, *NFIA*, *KLF2* in **b**) show differential gene set enrichment between the LumB subtype of breast cancer and normal tissue. **d** Heatmap plot showing 24 regulons differentially activated or enriched in the LumB subtype. **e** GSEA results of four representative regulons (*TBX1*, *PCGF2*, *MAX*, *DLX3* in **d**) show differential gene set enrichment between the LumB subtype of breast cancer and normal tissue. **f** Heatmap plot showing 28 regulons differentially activated or enriched in the basal subtype. **g** GSEA results of four representative regulons (*TFDP2*, *ESRRA*, *ZIC1*, *AEBP2* in **f**) show differential gene set enrichment between the basal subtype of breast cancer and normal tissue.


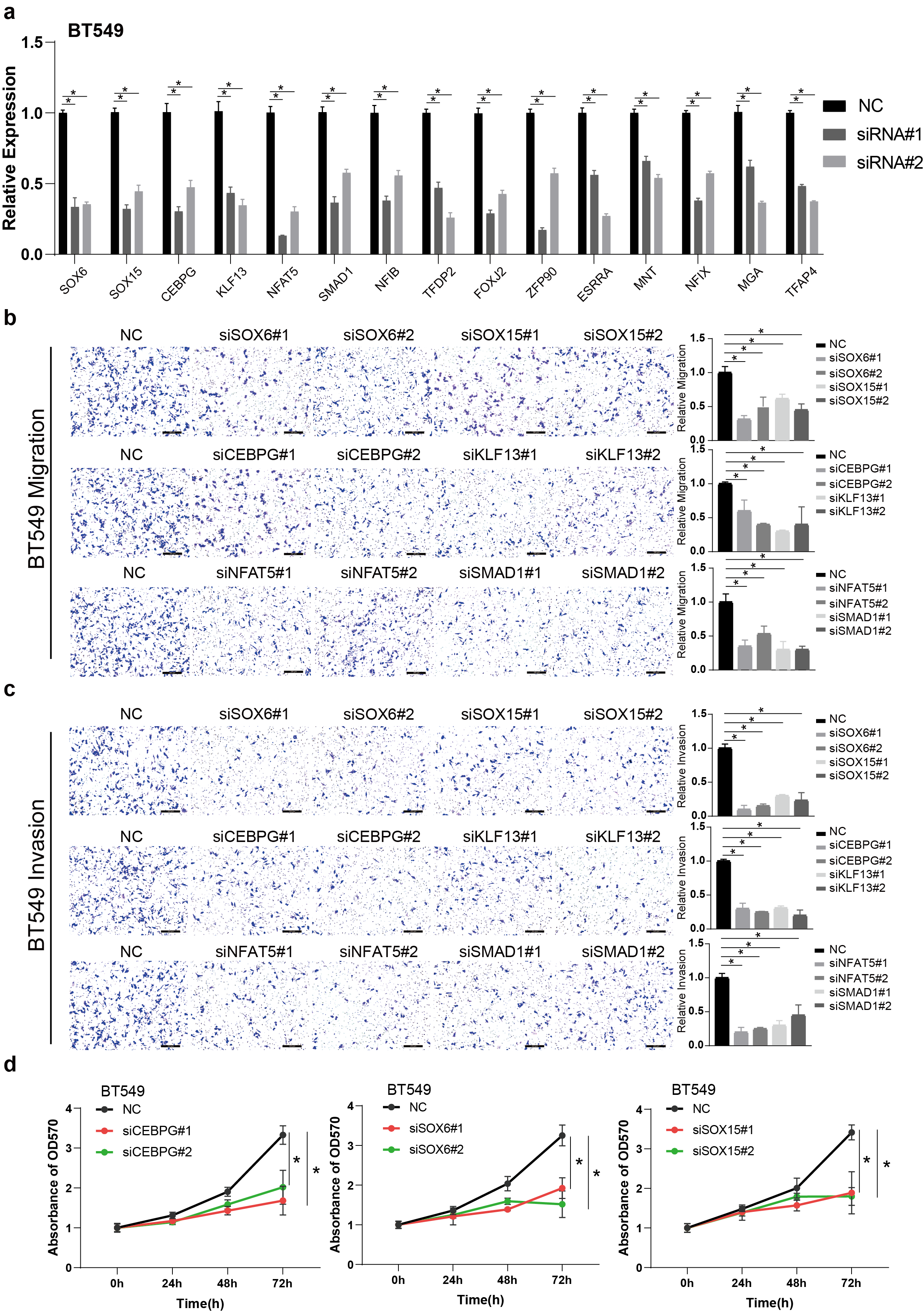


**Figure S13. The effect of basal subtype-specific activated TFs on breast cancer progression (BT549 cells). a** The mRNA expression of TFs by using RT-PCR to determine these genes knockdown by siRNA in BT549 cells. **b-c** Knockdown of TFs significantly inhibited the migration and invasion detected by transwell assay (magnification, 100×; Scale bars, 200 µm). **d** The effect of TFs on the proliferation of triple-negative breast cancer cells in vitro detected by growth curve assay. Statistical significance was represented by **P* < 0.05 (unpaired two-tailed Student’s t-test).


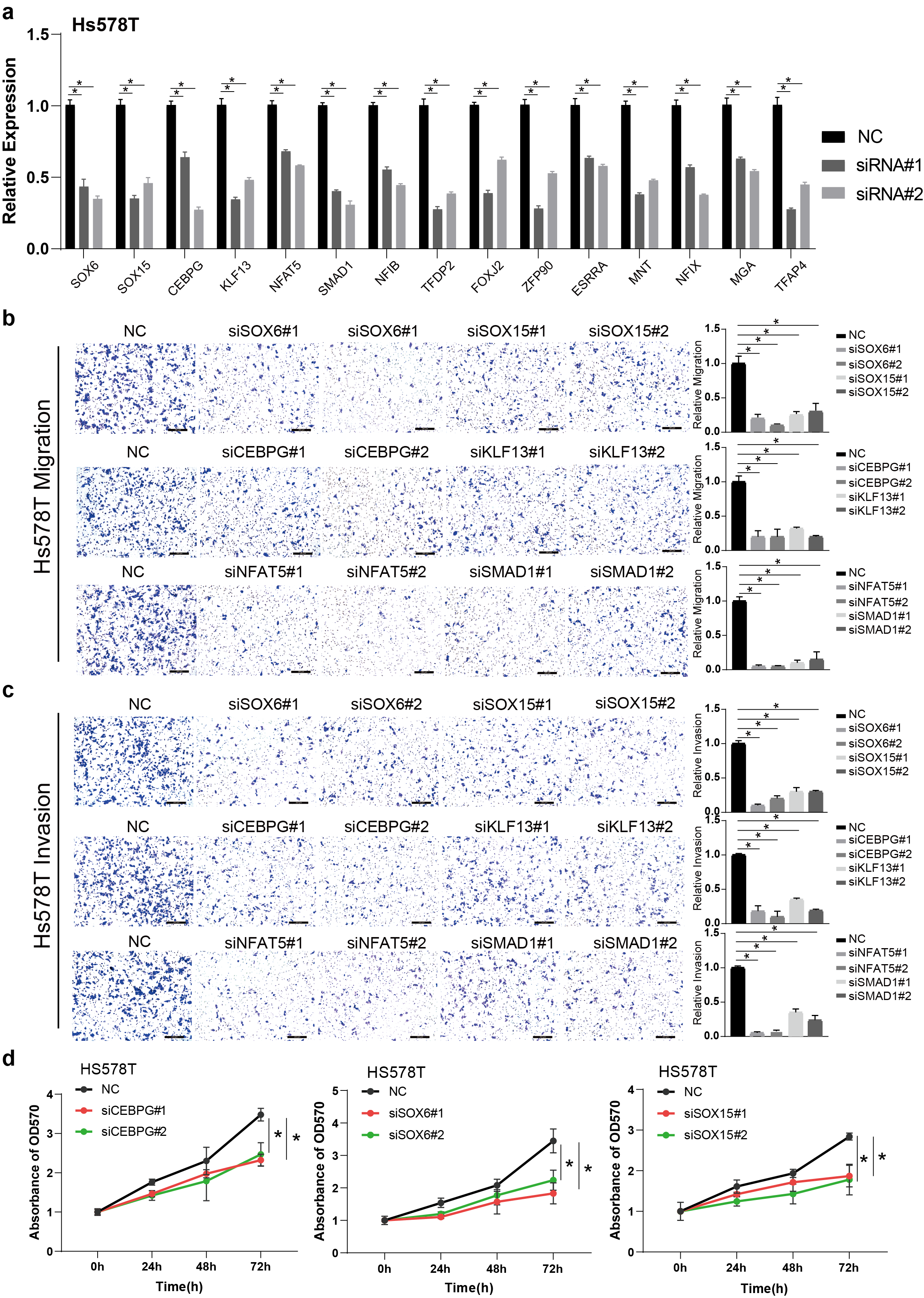


**Figure S14. The effect of basal subtype-specific activated TFs on breast cancer progression (Hs578T cells). a** The mRNA expression of TFs by using RT-PCR to determine these genes knockdown by siRNA in Hs578T cells. **b-c** Knockdown of TFs significantly inhibited the migration and invasion detected by transwell assay (magnification, 100×; Scale bars, 200 µm). **d** The effect of TFs on the proliferation of triple-negative breast cancer cells in vitro detected by growth curve assay. Statistical significance was represented by **P* < 0.05 (unpaired two-tailed Student’s t-test).


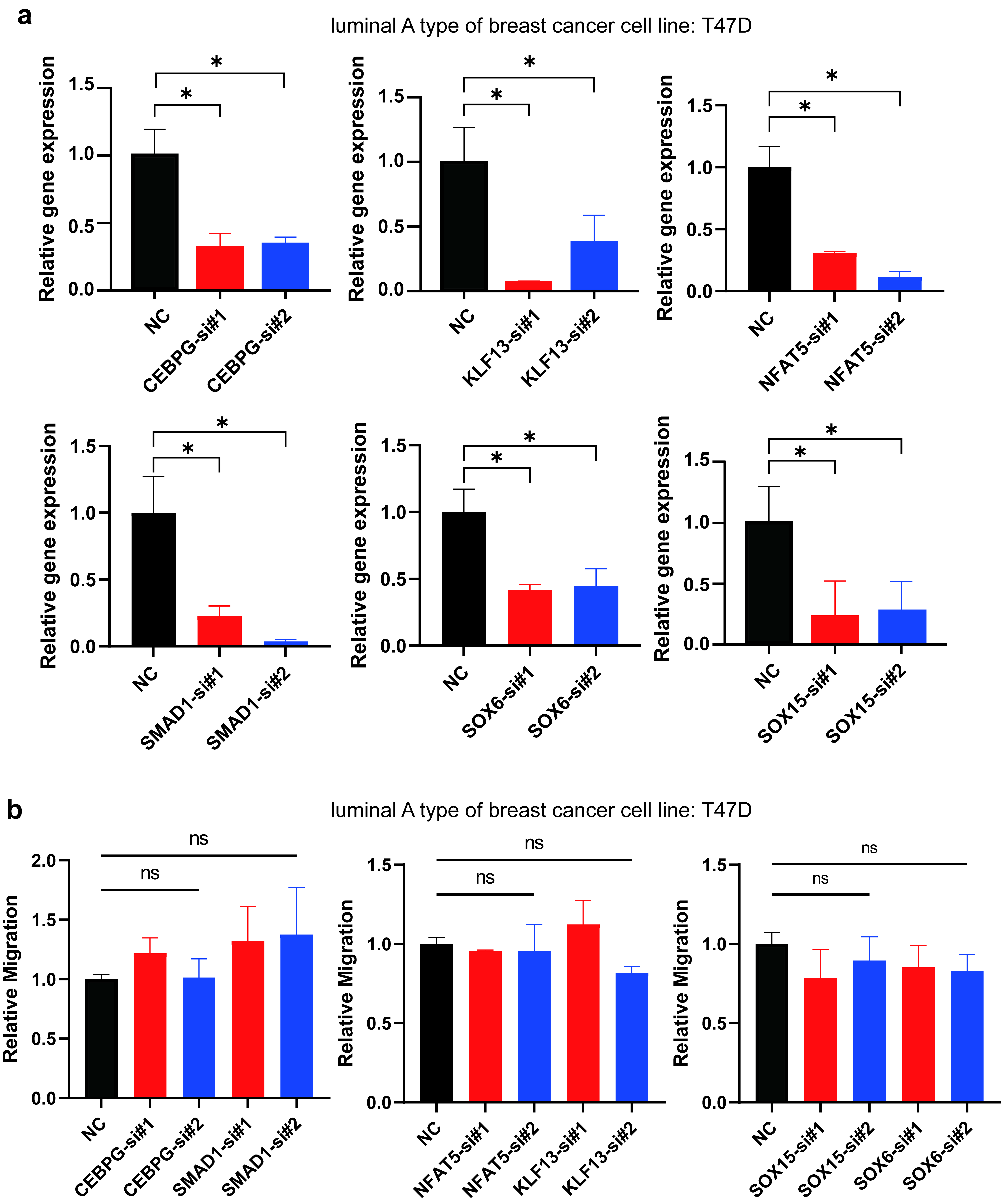


**Figure S15. The effect of basal subtype-specific activated TFs on luminal A breast cancer progression.** **a** The mRNA expression of TFs by using RT-PCR to determine these genes knockdown by siRNA in T47D cells (luminal A type of breast cancer cell line). **b** Knockdown of TFs significantly inhibited the migration detected by transwell assay. The data represent the means±SD from three independent experiments. Statistical significance was represented by **P* < 0.05 (unpaired two-tailed Student’s t-test).


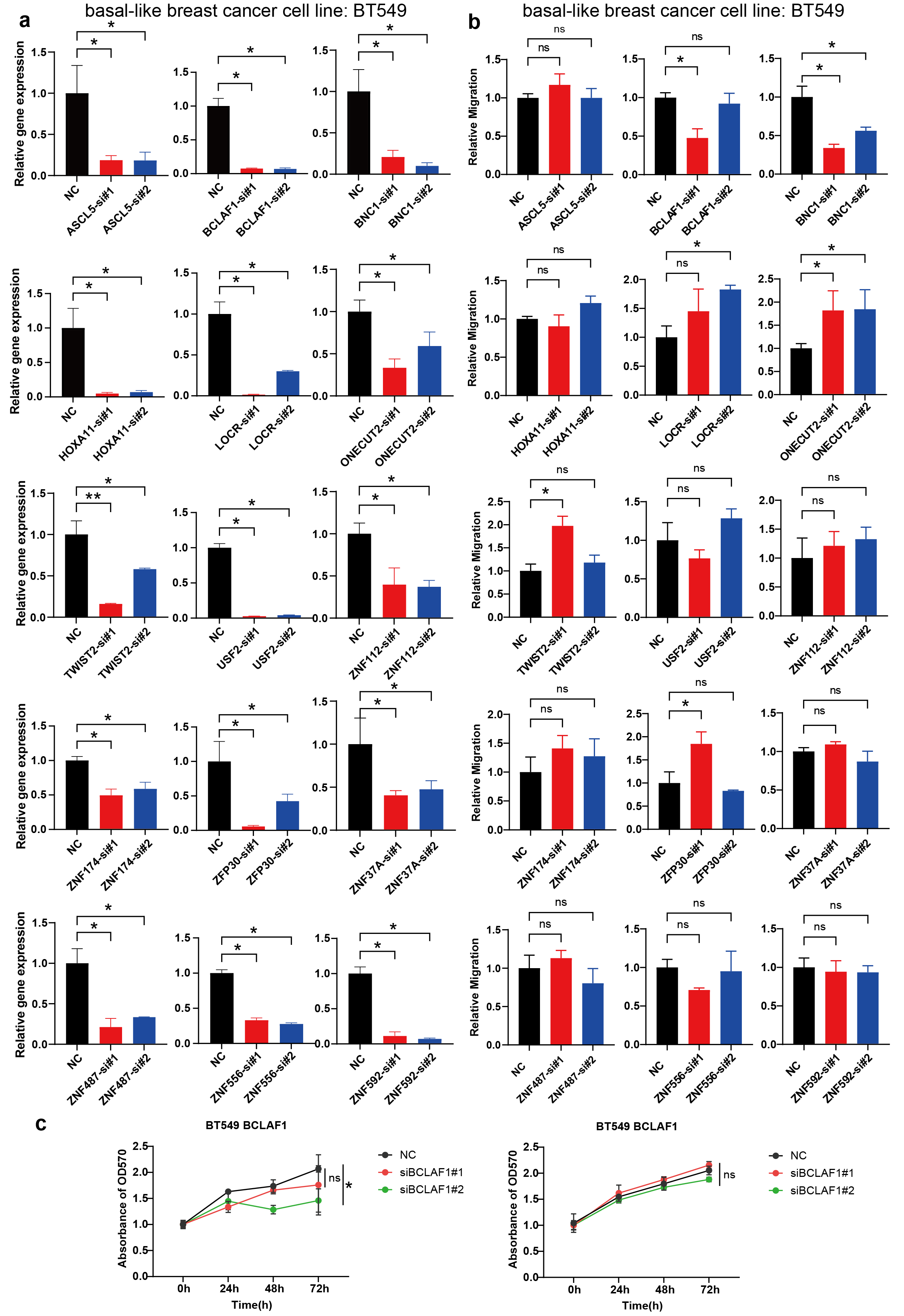


**Figure S16. The effect of first batch of 15 randomly selected TFs on breast cancer progression.** **a** The mRNA expression of TFs by using RT-PCR to determine these genes knockdown by siRNA in BT549 cells (base-like breast cancer cell line). **b** The barplot shows the impact of knockdown of 15 randomly selected TFs on the migration of BT549 cells. Only two TFs (BCN1 and BCLAF1) show statistical significance in inhibiting the migration of cancer cells. **c** The effect of two TFs (BCN1 and BCLAF1) on the proliferation of BT549 cells in vitro detected by growth curve assay. The data represent the means±SD from three independent experiments. Statistical significance was represented by **P* < 0.05 (unpaired two-tailed Student’s t-test).


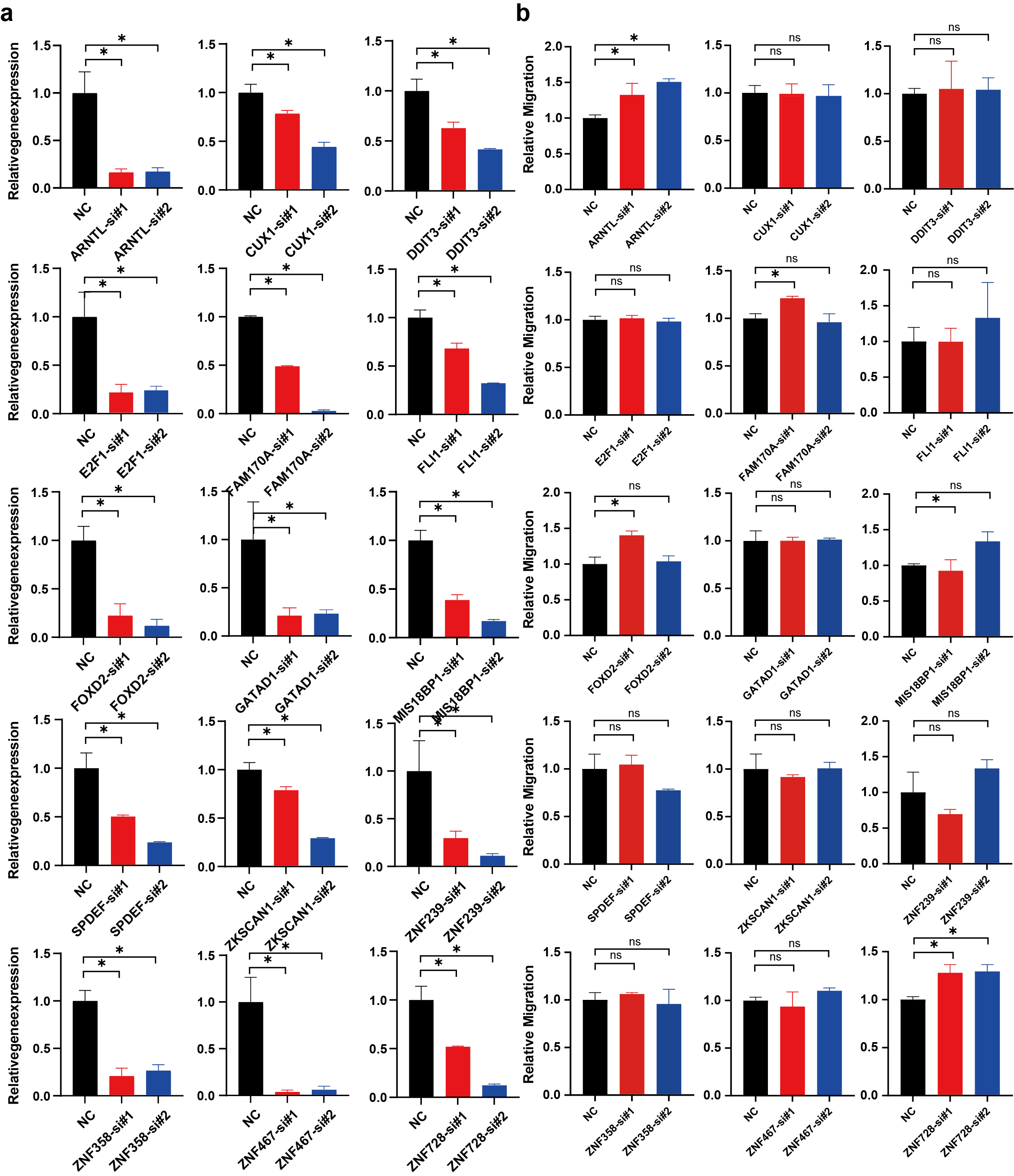


**Figure S17. The effect of second batch of 15 randomly selected TFs on breast cancer progression.** **a** The mRNA expression of TFs by using RT-PCR to determine these genes knockdown by siRNA in BT549 cells (base-like breast cancer cell line). **b** The barplot shows the impact of knockdown of 15 randomly selected TFs on the migration of BT549 cells. None of them show statistical significance in inhibiting the migration of cancer cells. The data represent the means±SD from three independent experiments. Statistical significance was represented by **P* < 0.05 (unpaired two-tailed Student’s t-test).


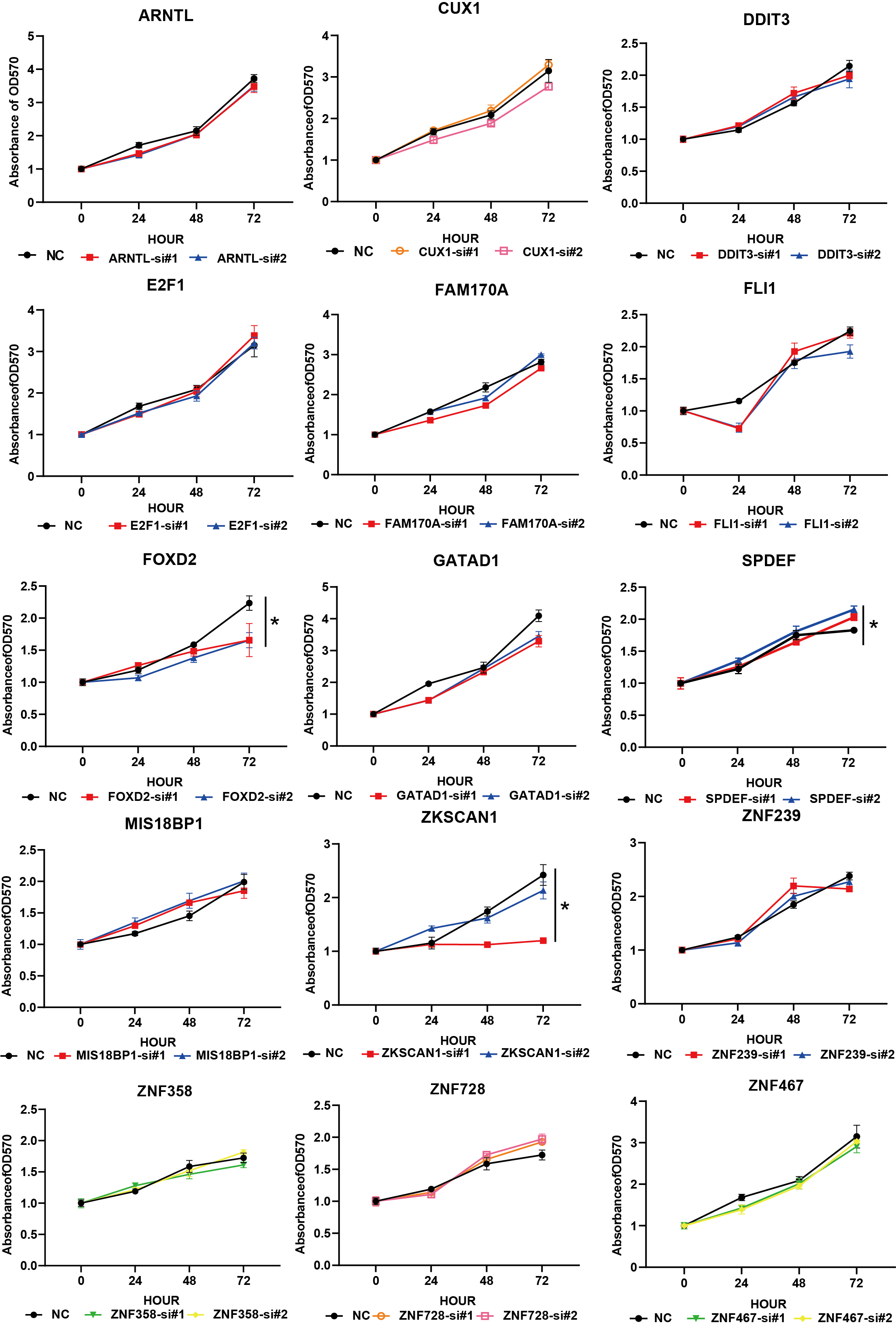


**Figure S18. The effect of second batch of 15 randomly selected TFs on breast cancer proliferation.** The effect of second batch of 15 randomly selected TFs on the proliferation of BT549 cells in vitro detected by growth curve assay. The data represent the means±SD from three independent experiments. Statistical significance was represented by **P* < 0.05 (unpaired two-tailed Student’s t-test).


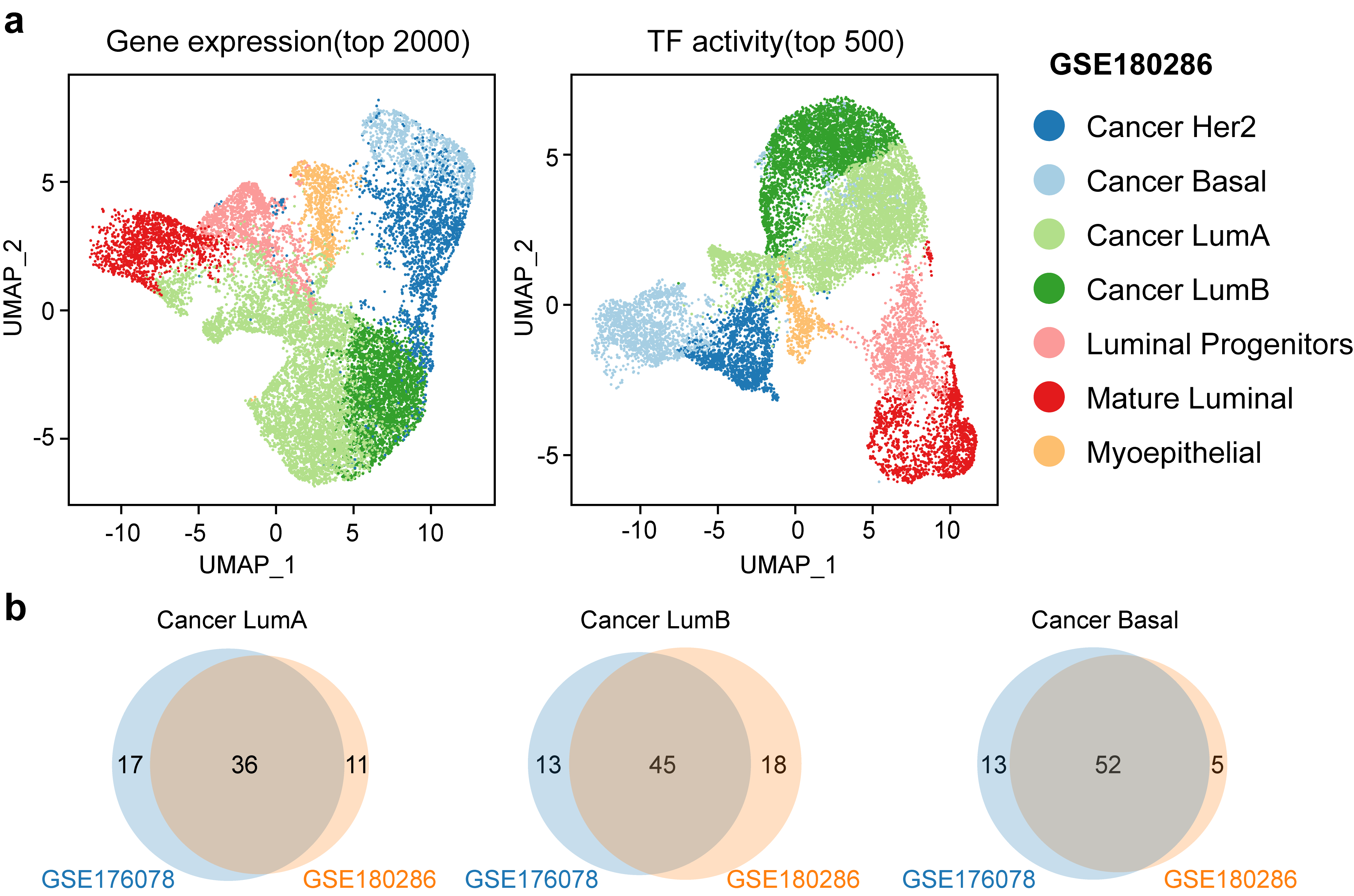


**Figure S19. metaTF results for the human breast cancer epithelial cells single-cell RNA-Seq dataset (GSE180286). a** UMAP plots visualizing the cluster assignments of human breast cancer epithelial cells based on TF activity profile (right) and highly variable genes expression profile (left). **b** The Venn diagram illustrates the repeatability of subtype-specific activated TFs analysis results across two sets of human breast cancer epithelial cell data.


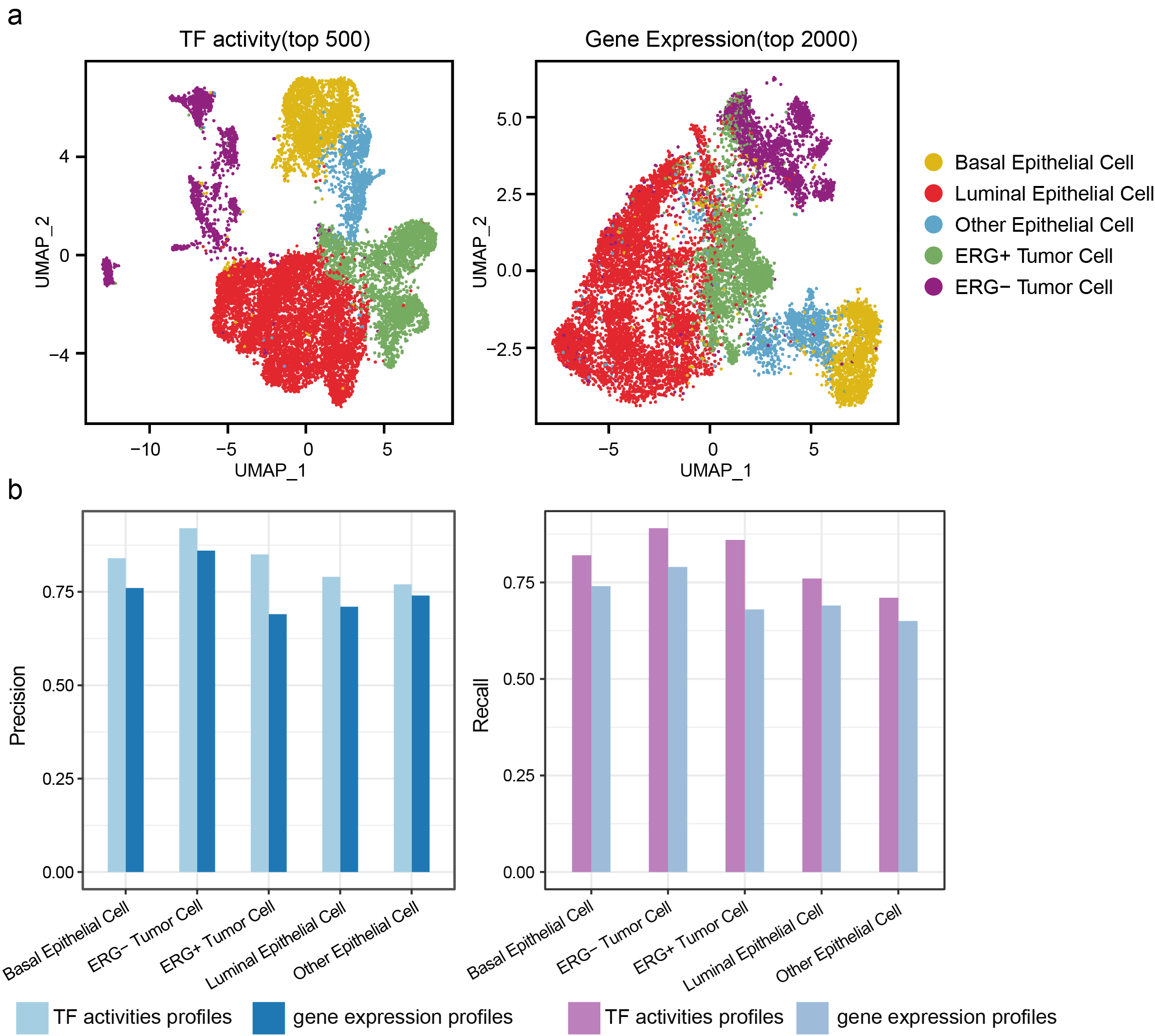


**Figure S20. metaTF results for the human prostate cancer epithelial cells single-cell RNA-Seq dataset (GSE176031).** **a** UMAP plots visualizing the cluster assignments of human prostate cancer epithelial cells based on TF activity profile (left) and highly variable genes expression profile (right). **b** classic machine learning random forest classification model were employed to fit TF activity profiles and gene expression profiles. The right panel displays the precision of both models across different cell populations, while the left panel depicts the recall of both models across various cell populations.


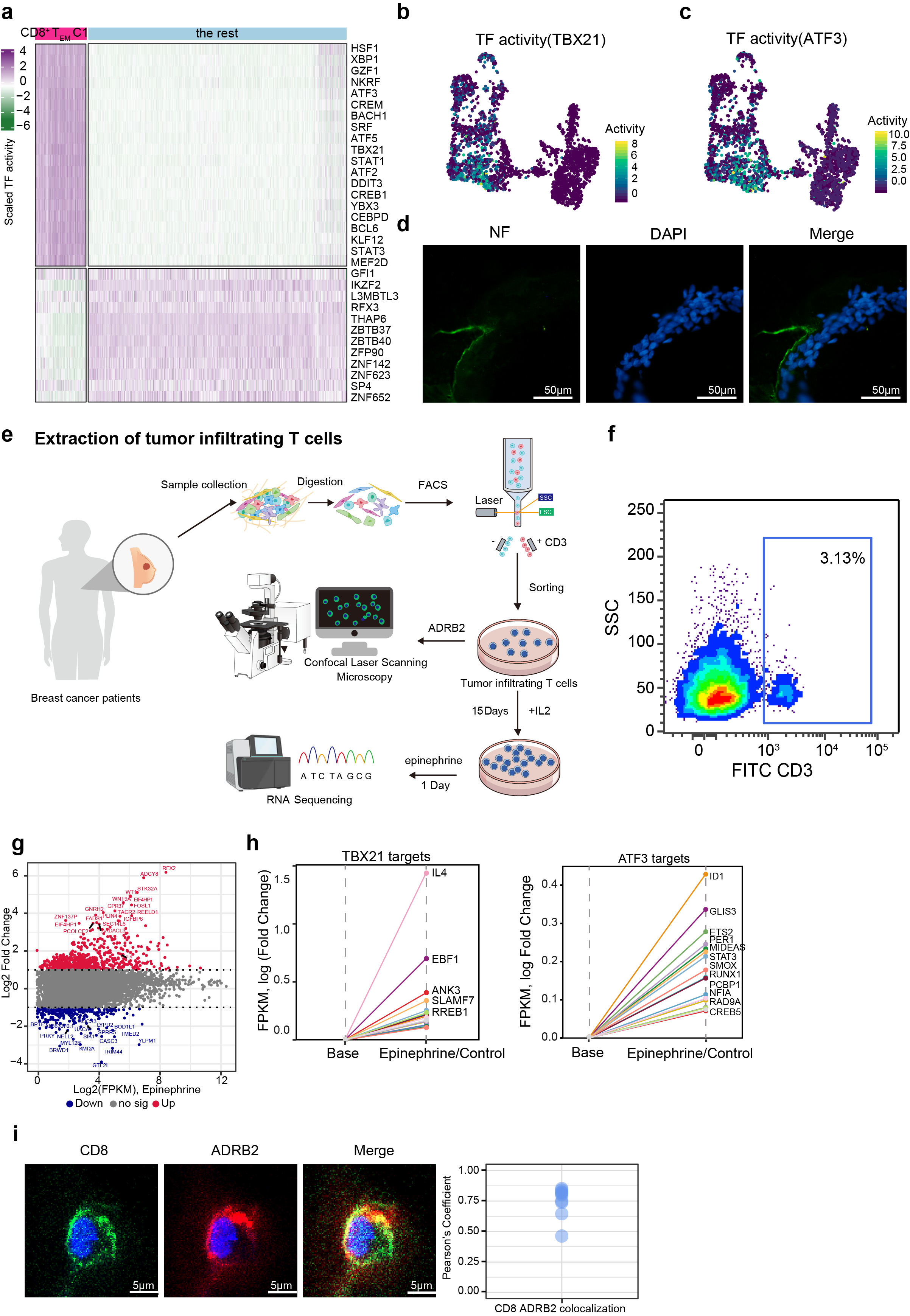


**Figure S21. Specific activated TFs in the CD8^+^ T_EM_ C1 cell population. a** Heatmap plot showing specific activated TFs in the CD8^+^ T_EM_ C1 cell population. **b,c** UMAP plot showing the TF activity of *TBX21* and *ATF3* according to the TF activity profile. **d** Representative immunostaining data showing neurofilaments maker (anti-neurofilament heavy, NF) expressed in breast cancer tissue. Green=NF, Blue=DAPI. Scale bar=50 μm. **e** Schematic overview of the experimental procedure in vitro cytological experiments. **f** Representative flow cytometry scatterplot illustrating the results of flow cytometry for assessing the proportion of T lymphocytes within breast cancer tissue. FITC, fluorescein isothiocyanate; SSC side scatter. **g** Quality control for epinephrine treatment and control RNA-Seq data. **h** Upregulated targets of *TBX21* and *ATF3* in epinephrine-treated RNA-seq data. **i** The expression of *CD8* and *ADRB2* in tumor-infiltrating lymphoid T cells from breast cancer patients, and the colocalization (Pearson’s coefficient) between CD8 and ADRB2 is shown, n = 10 cells.


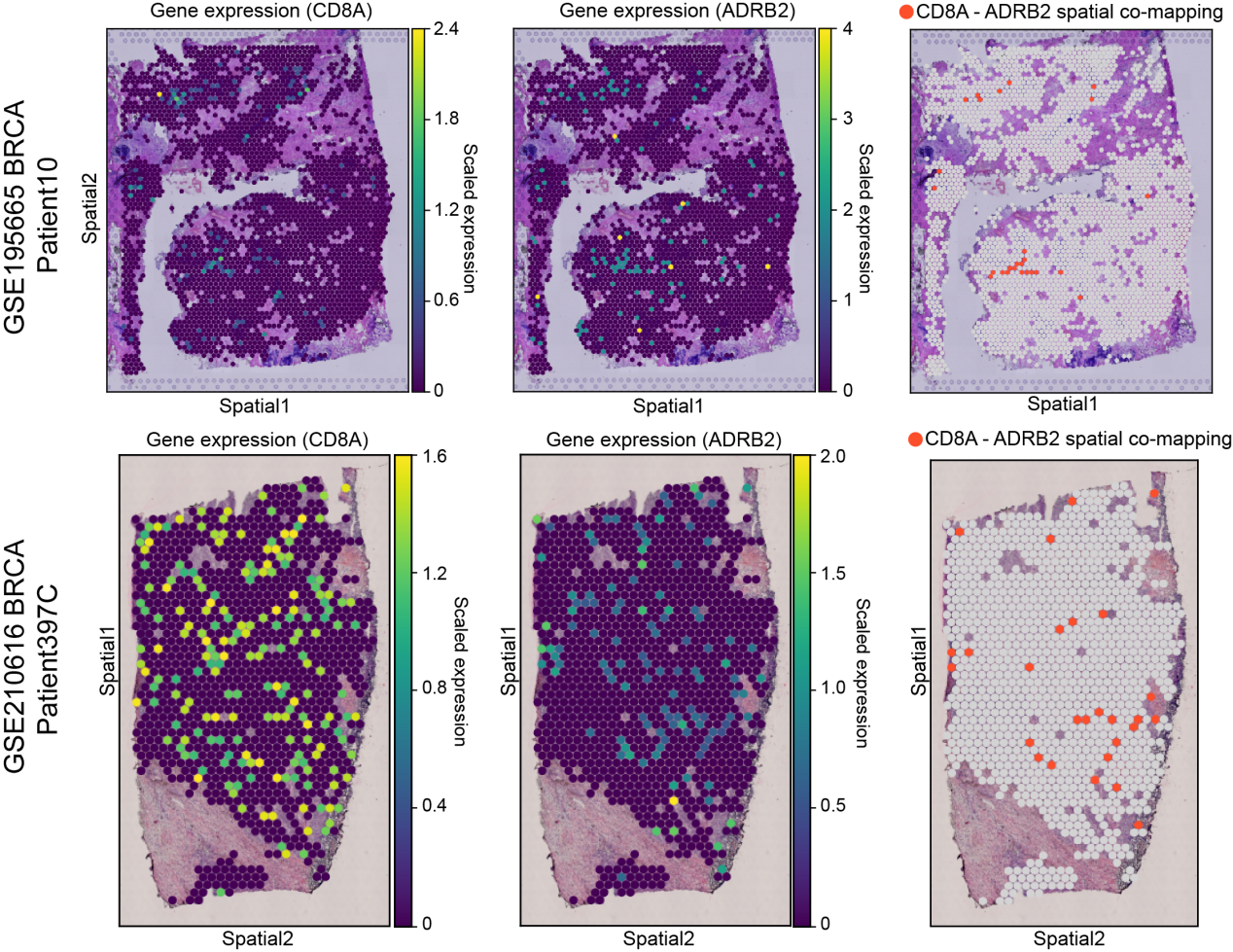


**Figure S22. Single-cell spatial transcriptome RNA-seq of two breast cancer patients.** Spatial co-mapping of *CD8* and *ADRB2* gene expression by spatial transcriptomics in two breast cancer patients with samples that are recorded in two public datasets.


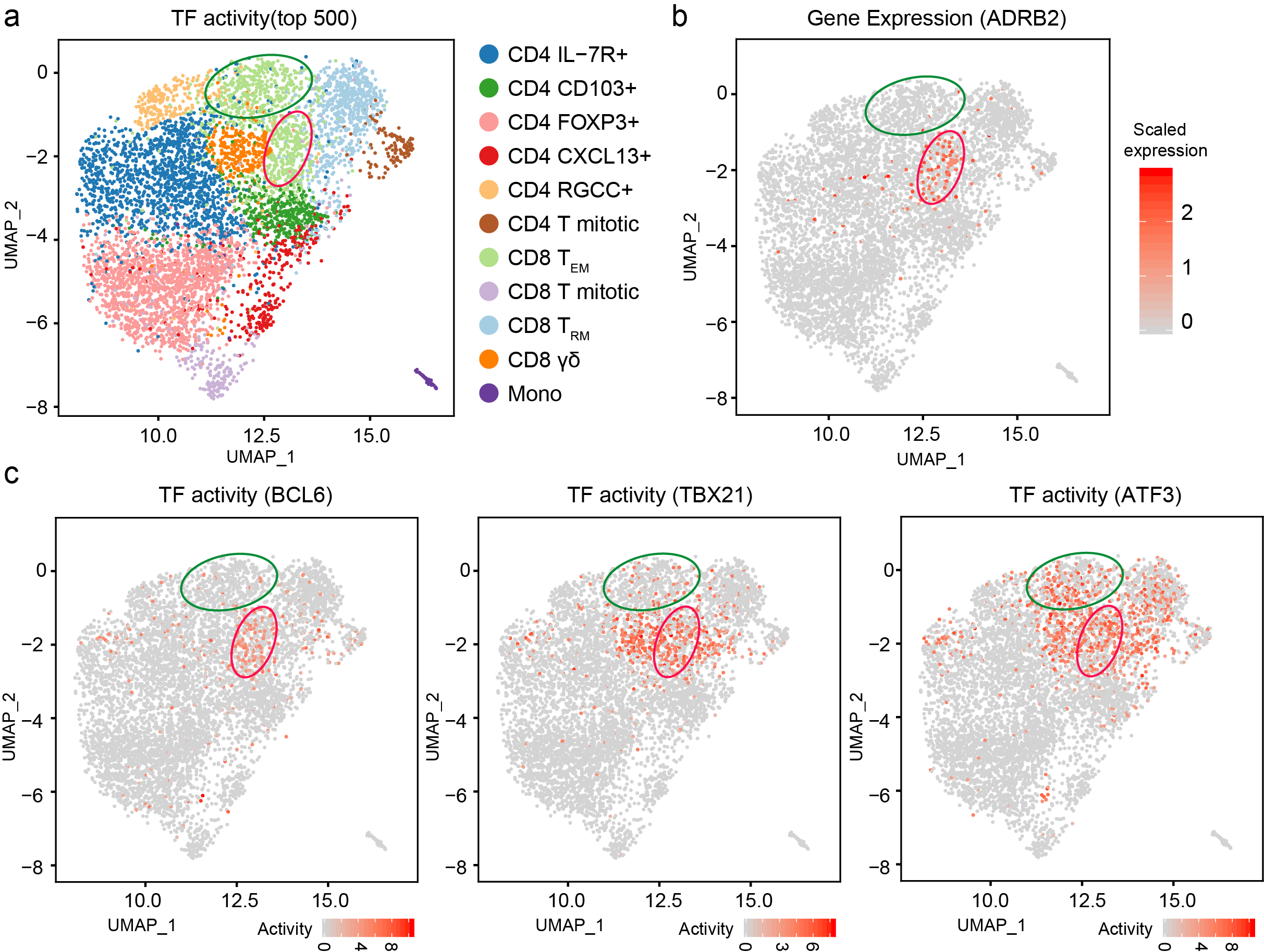


**Figure S23. The results of metaTF analysis on BRCA single-cell RNA sequencing data (GSE110686). a** UMAP plots visualizing cluster assignments of human breast cancer T cells based on TF activity profile. **b** Dot plot showing the significant expression of β2-adrenergic receptor (*ADRB2*) primarily in the one subset CD8^+^ T_EM_ cells. **c** UMAP plot showing the TF activity of *TBX21* and *ATF3* according to the TF activity profile.

**
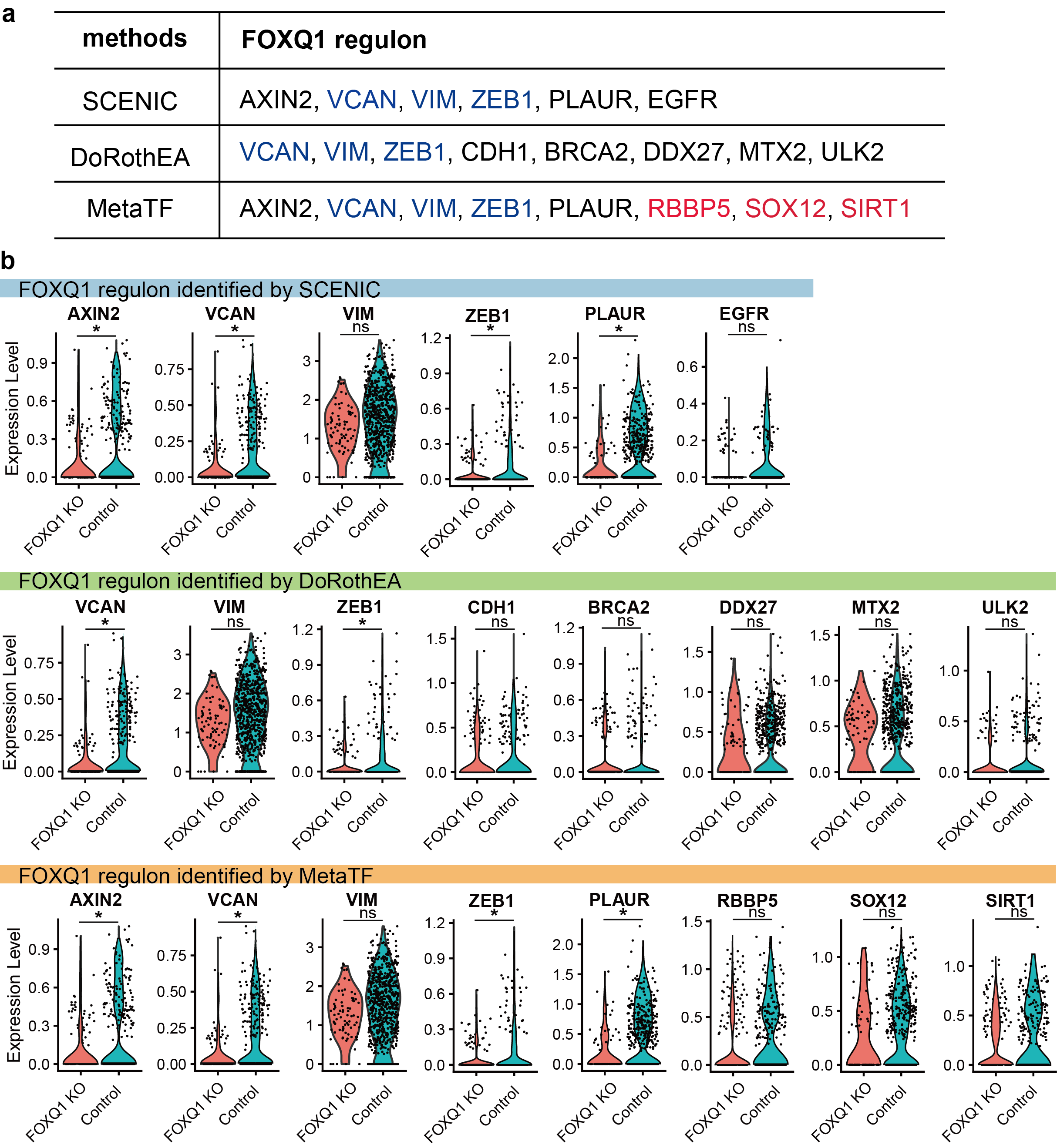
**

**Figure S24. The *FOXQ1* regulon identified by three methods. a** The target genes identified by the three methods constitute their respective regulons. Blue text indicates target genes identified by all three methods, while red text denotes target genes identified by metaTF that are expressed but show no significant difference when compared between knockout (KO) and control cells. **b** Expression of these target genes in cells with *FOXQ1* KO and control cells. A two-sided Wilcoxon rank-sum test was used to determine the difference (**P* < 0.05). ns, not significant.
